# Hardened Performance of 3D-Printed Geopolymer Mortars: A Review of Mechanical Properties, Durability, Sustainability, and Practical Implementation

**DOI:** 10.3390/polym18151843

**Published:** 2026-07-28

**Authors:** İbrahim Türkmen, Fatih Kantarcı, Enes Ekinci, Abdulrahman Ahmed Alymani, Mehmet Burhan Karakoç, Yaşar Ayaz, Ergun Ekinci, Ramazan Demirboğa

**Affiliations:** 1 Department of Architectural Engineering, College of Engineering and Advanced Computing, Alfaisal University, Riyadh 11564, Saudi Arabia; iturkmen@alfaisal.edu (İ.T.); abalymani@alfaisal.edu (A.A.A.);; 2 Department of Civil Engineering, Inonu University, Malatya 44280, Türkiye; enes.ekinci@inonu.edu.tr (E.E.); mehmet.karakoc@inonu.edu.tr (M.B.K.);; 3 Department of Chemistry, Inonu University, Malatya 44280, Türkiye

**Keywords:** 3D concrete printing, strength, durability, sustainability

## Abstract

3D-printed geopolymer mortars (3DPGPMs) are emerging as low-carbon construction materials that combine digital fabrication with alkali-activated binder technology. However, their hardened performance remains difficult to assess because it is controlled not only by geopolymer chemistry but also by printing parameters, rheological evolution, curing conditions, interlayer bonding, pore structure, and loading direction. This review critically examines the current literature on extrusion-based 3DPGPMs, with emphasis on mechanical properties, durability, sustainability, standardization, and practical implementation. The reviewed studies show that precursor type, activator system, aggregate/binder ratio, additives, printing conditions, and curing regime strongly influence compressive, tensile, flexural, interlayer bond, and anisotropic mechanical responses. Durability performance is also governed by the coupled effects of matrix chemistry and printing-induced features, including interlayer voids, directional pore networks, weak interfaces, and transport pathways that may affect shrinkage, water absorption, chloride penetration, carbonation, acid and sulphate resistance, freeze–thaw response, and elevated-temperature behavior. From a sustainability perspective, the environmental benefits of 3DPGPMs are conditional and depend on activator production, precursor availability, curing demand, transport distance, life-cycle assessment boundaries, and field-scale implementation conditions. The review identifies that the main knowledge gap is the limited availability of integrated datasets linking fresh-state rheology, interlayer quality, multi-scale porosity, mechanical anisotropy, durability indicators, and structural-scale validation. Future research should therefore prioritize standardized reporting, performance-based acceptance criteria, long-term exposure testing, field-scale validation, and predictive material–process–durability models. Overall, this review provides a hardened-performance-oriented synthesis to support the development of reliable, durable, and sustainable 3DPGPMs for construction applications.

## 1. Introduction

The construction industry is under increasing pressure to improve productivity, reduce labor dependency, minimize material waste, and lower the environmental burden associated with conventional construction practices [1]. In this context, extrusion-based 3D concrete printing (3DCP) has emerged as a promising additive manufacturing technology because it enables automated layer-by-layer fabrication from digitally defined toolpaths and reduces or eliminates the need for formwork [2]. In addition, 3DCP offers design flexibility for producing geometrically complex elements that are difficult to realize using conventional casting methods [1].

Unlike conventionally cast cementitious materials, 3D-printed mortars must simultaneously satisfy several fresh-state requirements, including pumpability, extrudability, shape retention, buildability, and sufficient interlayer bonding. Therefore, their hardened performance depends not only on mixture design but also on process-related parameters such as printing speed, nozzle geometry, layer height, time interval between successive layers, and environmental conditions during printing [1,3].

The search for low-carbon binders has intensified due to the high energy demand and CO_2_ emissions associated with ordinary Portland cement production [4]. Geopolymer binders have attracted considerable attention because they can incorporate aluminosilicate-rich precursors such as fly ash (FA), ground granulated blast-furnace slag (GGBFS), metakaolin (MK), and other waste-derived materials [4]. However, the environmental benefits of geopolymer systems are not automatic; in contrast, they depend strongly on sodium silicate demand, activator-related impacts, precursor allocation assumptions, curing requirements, and life-cycle assessment (LCA) boundaries [4].

**Figure 1:**
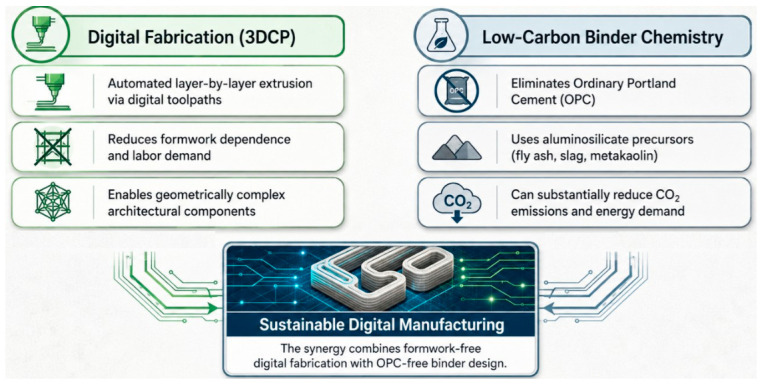
Dual sustainability pathway of 3D-printed geopolymer systems through digital fabrication and low-carbon binder chemistry. Conceptual illustration created by the authors based on the synthesis of Refs. [5,6,7,8].

The combination of 3D printing and geopolymer binders is particularly attractive because it links two sustainability-oriented strategies: digital, formwork-free manufacturing and ordinary Portland cement (OPC) reduced or OPC-free binder chemistry [1,3]. This dual mandate of combining digital fabrication with low-carbon binder chemistry is conceptually summarized in Figure 1. Nevertheless, 3D-printed geopolymer mortars (3DPGPMs) cannot be evaluated simply as printed versions of conventional cast geopolymers. Their performance is governed by the coupled effects of geopolymer reaction kinetics, rheological evolution, thixotropic build-up, setting response, curing sensitivity, moisture state, temperature–humidity conditions, interlayer bonding, and the anisotropic pore structure generated during layer-by-layer deposition [1,3].

**Figure 2:**
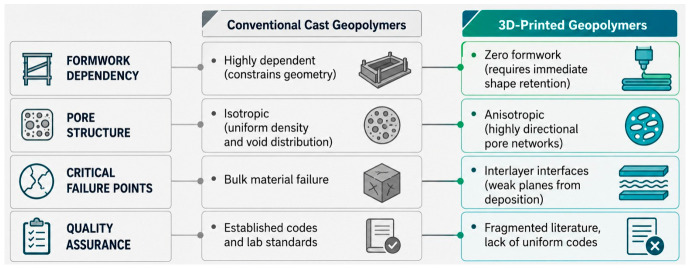
Material paradigm shift from cast to 3D-printed geopolymer systems. Conceptual synthesis based on [1,2,10].

A key feature distinguishing 3DPGPMs from cast geopolymer mortars is the presence of layer interfaces. When interlayer bonding is insufficient, these interfaces may act as preferential weak planes under mechanical loading and may contribute to direction-dependent tensile and flexural behavior [2]. Interlayer bond strength is affected by open time, surface moisture, rheological evolution, structuration rate, mechanical interlocking, and chemical bonding between successive layers [2,9]. In one-part geopolymer 3D printing, microwave-induced acceleration of polycondensation can improve interlayer bond strength, although excessive heating may increase surface moisture loss; therefore, reaction acceleration and moisture retention should be balanced during layer deposition [9]. The resulting material paradigm shift from cast to 3D-printed geopolymer systems is summarized in Figure 2.

Durability should also be interpreted by considering both the interface-related features of layer-by-layer deposition and the binder-specific degradation mechanisms of alkali-activated systems. In alkali-activated materials, carbonation and chloride penetration are governed by precursor chemistry, calcium and magnesium content, activator composition, silicate modulus, curing conditions, pore structure, and the type of reaction products formed, including N-A-S-H, C-A-S-H, and C-(A)-S-H type gels [11]. Acid exposure is also closely related to gel chemistry because hydrogen ions can attack N-A-S-H and C-A-S-H gels, leading to degradation of the binder matrix and reduced durability under acidic conditions [12]. In printed systems, these chemical mechanisms may be further influenced by interlayer voids, anisotropic pore networks, and direction-dependent transport pathways.

These durability-related mechanisms are ultimately governed by the fundamental reaction processes of geopolymer binders. Geopolymerization begins with the alkaline dissolution of reactive aluminosilicate phases in precursors such as FA, GGBFS, MK, or other waste-derived materials. Under highly alkaline conditions, Si–O–Si and Si–O–Al bonds are progressively broken, releasing silicate and aluminate species into the pore solution. These dissolved species then reorganize, form reaction gels, and undergo polycondensation to create three-dimensional aluminosilicate networks. In low-calcium systems, the dominant reaction product is generally N-A-S-H gel, whereas calcium-rich systems containing GGBFS may also form C-A-S-H and hybrid C-(A)-S-H type gels [11,12]. The relative formation of these gels depends on precursor chemistry, calcium content, activator composition, alkali concentration, silicate modulus, water availability, curing temperature, and reaction time [4,11]. Therefore, the reaction mechanism of geopolymer binders directly controls setting behavior, rheological build-up, open time, early-age stiffness development, pore refinement, interlayer bond formation, mechanical strength, and durability-related transport resistance in 3DPGPMs.

Despite the growing number of studies on 3D-printed cementitious materials and geopolymer-based printable mixtures, the literature on 3DPGPMs remains fragmented. Existing reviews and experimental studies often focus separately on printability, rheology, interlayer bonding, mechanical performance, durability, microstructure, or sustainability [13]. As a result, integrated evaluations connecting mixture design, printing parameters, interlayer quality, hardened mechanical performance, durability, and sustainability within the same 3D-printed geopolymer system remain limited. In particular, long-term durability data are still insufficient for extrusion-based 3D-printed geopolymer systems, especially under acid, sulphate, salt, moisture absorption, efflorescence, freeze–thaw, and corrosion-related exposure conditions [13].

The lack of uniform codes and guidelines is another major barrier to practical implementation because conventional concrete test methods and preparation procedures are not directly suitable for geopolymer concrete or 3D-printed geopolymer systems [13]. From a qualification perspective, additive construction also requires process-based quality assurance, including feasibility assessment, validation planning, process qualification, documentation, traceability, and quality control of process-relevant steps [14].

Accordingly, this review aims to synthesize the current knowledge on the hardened performance of extrusion-based 3D-printed geopolymer and alkali-activated mortars, with emphasis on mechanical properties, durability behavior, sustainability, standardization, and practical implementation. The review discusses how precursor type, activator system, curing regime, printing parameters, interlayer quality, and pore structure influence compressive, tensile, and flexural performance, transport-related durability, high-temperature response, and environmental impact. By identifying current contradictions, knowledge gaps, and future research needs, this study provides a performance-oriented framework for advancing 3DPGPMs from laboratory-scale mixtures toward reliable and sustainable construction applications.

## 2. Review Methodology and Scope

This review was designed as a structured narrative review informed by systematic search and screening principles, rather than as a PRISMA-compliant systematic review or meta-analysis. This approach was adopted to provide a transparent basis for identifying, screening, and synthesizing relevant studies while maintaining the flexibility required to evaluate a rapidly developing and interdisciplinary research field. This approach was adopted to provide a transparent basis for identifying, screening, and synthesizing relevant studies while maintaining the flexibility required to evaluate a rapidly developing and interdisciplinary research field.

The main objective of this review is to synthesize the current knowledge on the hardened performance of 3DPGPMs by linking mechanical properties, durability behavior, sustainability-related considerations, and practical implementation challenges. This integrated perspective is necessary because the performance of geopolymer-based printable materials is controlled not only by binder chemistry, but also by printability, rheology, structural build-up, interlayer bonding, curing regime, and printing-induced anisotropy.

The scope of the review was limited primarily to extrusion-based 3D-printed geopolymer and alkali-activated mortar systems. This limitation was adopted because extrusion-based printing is the dominant approach in 3D concrete printing research and creates specific hardened-performance issues related to layer interfaces, open time, shape retention, interlayer bond quality, anisotropy, and direction-dependent mechanical behavior.

Particular attention was given to studies reporting hardened-state performance, including compressive strength, tensile and flexural strength, elastic modulus, interlayer bond strength, anisotropy, shrinkage, water absorption, permeability, chloride penetration, carbonation, acid and sulphate resistance, freeze–thaw resistance, and elevated-temperature behavior. These parameters were selected because long-term durability and interlayer quality remain among the most critical limitations for the practical implementation of 3D-printed geopolymer systems.

Studies dealing with LCA, carbon footprint, alkali activator impacts, waste-derived precursors, one-part geopolymer systems, standardization, and quality-control challenges were also considered. Their inclusion is important because the environmental and practical benefits of geopolymer systems depend strongly on precursor selection, activator production, curing requirements, allocation assumptions, LCA boundaries, and reliable performance-based qualification procedures.

Although several recent reviews have addressed 3D concrete printing, geopolymer printability, rheological performance, and alkali-activated binders, the literature remains fragmented with respect to the integrated assessment of hardened mechanical properties, durability mechanisms, sustainability, and implementation requirements. Therefore, this review focuses specifically on the hardened performance of 3DPGPMs and evaluates how precursor type, activator system, printing parameters, interlayer quality, pore structure, and curing regime influence long-term performance.

### 2.1. Literature Search Strategy

A structured literature search was conducted using major scientific databases, including Scopus, Web of Science Core Collection, ScienceDirect, SpringerLink, Taylor & Francis Online, MDPI, and Google Scholar. Multiple databases were used to improve literature coverage, reduce database-dependent bias, and identify both experimental studies and review papers relevant to 3DPGPMs. The main search period was defined as 2015 onward, since research on extrusion-based 3D concrete printing and geopolymer-based printable binders has expanded rapidly during this period. Earlier foundational studies were also included when necessary to explain geopolymer chemistry, alkali-activated binder behavior, durability mechanisms, or LCA assumptions.

The keyword strategy was developed according to the main focus of this review, namely the hardened performance of 3DPGPMs. The first group of keywords targeted the material system and manufacturing process, using terms related to 3D-printed geopolymers, geopolymer 3D printing, 3D-printed alkali-activated mortars, extrusion-based geopolymer printing, additive manufacturing in geopolymer construction, and printable geopolymer binders. Additional keyword groups were used to capture studies dealing with printability-related parameters, mechanical performance, durability, sustainability, and standardization. These included terms related to pumpability, extrudability, buildability, shape retention, open time, setting time, thixotropy, rheology, compressive strength, flexural strength, tensile strength, elastic modulus, interlayer bond strength, anisotropy, layer orientation, printing direction, fiber reinforcement, water absorption, sorptivity, permeability, chloride penetration, carbonation, acid and sulphate attack, freeze–thaw resistance, elevated temperature, shrinkage, cracking, LCA, carbon footprint, alkali activator impacts, one-part geopolymers, waste-derived precursors, ISO/ASTM 52939 [14], RILEM 3D concrete printing, and quality control.

These keyword groups were selected because the hardened performance of 3DPGPMs is governed by the coupled effects of material composition, fresh-state behavior, printing parameters, interlayer quality, curing regime, pore structure, durability-related transport properties, and environmental performance. In particular, fresh-state parameters were included only when they were connected to hardened performance, since printability, rheology, and open-time evolution directly affect layer deposition, interlayer bonding, dimensional stability, and the resulting mechanical and durability behavior.

The search results were first screened by title and abstract. Potentially relevant studies were then examined in full text to determine whether they directly addressed 3D-printed geopolymer or alkali-activated systems, or whether they provided essential background for comparison with OPC-based 3D-printed materials. Reference lists of key review papers and highly relevant experimental studies were also examined to identify additional sources and improve literature coverage beyond database searches. Experimental studies were prioritized when extracting information on mixture composition, printing parameters, curing regime, mechanical performance, durability indicators, and sustainability-related outcomes, while review papers were used to map broader research trends, terminology, classification approaches, standardization issues, and knowledge gaps.

To improve methodological transparency, the literature retrieval and classification procedure was organized into sequential stages. First, records were identified from Scopus, Web of Science Core Collection, ScienceDirect, SpringerLink, Taylor & Francis Online, MDPI, and Google Scholar using the keyword groups described above. Second, obviously irrelevant records, duplicate items, non-English publications, and studies outside the scope of cementitious or geopolymer-based additive manufacturing were excluded during preliminary screening. Third, titles and abstracts were assessed to identify publications related to extrusion-based 3D printing, geopolymer or alkali-activated binders, hardened mechanical performance, durability, sustainability, standardization, reinforcement strategies, or digital quality control. Fourth, potentially relevant studies were evaluated in full text according to the inclusion and exclusion criteria described in Section 2.2. Finally, the selected publications were classified according to their main contribution to the hardened-performance-oriented review framework.

Because the present study was designed as a structured narrative review rather than a full bibliometric or meta-analytical study, complete numerical records of all initially retrieved, duplicate-removed, and excluded publications were not available for retrospective reporting. Therefore, a PRISMA-type quantitative screening diagram was not presented. Instead, to avoid unsupported record-count claims, the revised manuscript provides a transparent qualitative workflow and a descriptive bibliometric mapping of the final selected reference set used in the review.

### 2.2. Inclusion and Exclusion Criteria

The literature was selected according to predefined inclusion and exclusion criteria to maintain the focus of the review on the hardened performance of 3DPGPMs. Studies were included if they investigated extrusion-based 3D-printed geopolymer, alkali-activated, or geopolymer-like mortar/concrete systems and reported hardened-state properties such as compressive strength, tensile strength, flexural strength, elastic modulus, stress–strain response, interlayer bond strength, fracture behavior, or mechanical anisotropy. Studies dealing with durability-related properties, including water absorption, sorptivity, permeability, chloride penetration, carbonation, acid resistance, sulphate resistance, freeze–thaw resistance, shrinkage, cracking, and elevated-temperature behavior, were also included because long-term performance and environmental resistance remain key unresolved issues for the broader implementation of 3D-printed geopolymer systems.

Studies addressing sustainability-related aspects were also considered when they focused on LCA, carbon footprint, alkali activator impacts, one-part geopolymer systems, waste-derived precursors, local resource utilization, or practical implementation. This criterion was adopted because the environmental performance of geopolymer binders may vary significantly depending on sodium silicate use, alkali activator production, precursor source, curing requirements, allocation assumptions, and LCA boundaries. Review papers, perspective articles, and standardization documents were included when they provided relevant synthesis, terminology, classification, research gaps, or guidance on 3D concrete printing, geopolymer binders, additive construction, interlayer bond testing, process qualification, or quality control.

OPC-based 3D-printed concrete studies were included only when they provided transferable information on interlayer bonding, anisotropy, printing direction, durability testing, standardization, or process-related quality control, and only when such information was directly relevant to interpreting the performance of 3DPGPMs. Conversely, studies were excluded if they focused exclusively on ordinary Portland cement-based 3D-printed concrete without providing transferable information related to interlayer bonding, anisotropy, durability, or standardization. Studies dealing only with fresh-state rheology or printability were also excluded when they did not establish any connection with hardened mechanical performance, durability, interlayer quality, or microstructural development.

To maintain a clear distinction between different evidence types, the reviewed literature was interpreted using an evidence hierarchy. Direct evidence from extrusion-based 3D-printed geopolymer or alkali-activated mortar/concrete systems was prioritized when drawing conclusions about 3DPGPM performance. Evidence from cast geopolymer, alkali-activated, or hybrid geopolymer systems was used only as indirect background for interpreting binder chemistry, reaction products, pore refinement, and durability mechanisms. Evidence from OPC-based 3D-printed concrete or ECC-type printed composites was treated as transferable but non-geopolymer-specific evidence and was used only to discuss layer interfaces, anisotropy, printing-induced defects, process-related quality control, or durability-testing concepts. Such indirect evidence was not used to define direct quantitative performance thresholds for 3DPGPMs, but rather to support cautious interpretation where direct 3DPGPM data remain limited.

Studies on metal, polymer, ceramic, asphalt, soil, or other non-cementitious additive manufacturing systems were excluded unless they provided directly transferable methodology for quality control, digital monitoring, or layer-interface assessment. Conventional cast geopolymer concrete or mortar studies were generally excluded unless they provided essential background on geopolymer chemistry, durability mechanisms, gel formation, activator effects, or LCA assumptions. Publications without sufficient technical detail on material composition, printing method, curing regime, or testing conditions were not used for detailed comparison, although they were occasionally considered for general contextual discussion. Duplicate records, inaccessible full texts, and publications not written in English were excluded from the detailed synthesis.

### 2.3. Review Framework

The selected literature was organized into three interconnected review axes: mechanical performance, durability, and sustainability/standardization. This framework was adopted because the hardened performance of 3DPGPMs is controlled by the combined effects of geopolymer binder chemistry, mixture design, curing regime, printing parameters, and printing-induced structural features.

The first axis, mechanical performance, covers compressive strength, tensile strength, flexural strength, elastic modulus, stress–strain behavior, interlayer bond strength, fracture behavior, mechanical anisotropy, fiber reinforcement, fiber–matrix interaction, and continuous reinforcement strategies. Particular emphasis is placed on interlayer bonding, layer orientation, and loading direction because the layer-by-layer production process creates direction-dependent behavior and potential weak planes that are not typically observed in the same way in conventionally cast geopolymer mortars.

The second axis, durability, covers transport-related and exposure-related properties, including water absorption, sorptivity, permeability, chloride penetration, carbonation, acid attack, sulphate attack, freeze–thaw resistance, shrinkage, cracking, and elevated-temperature performance. These properties are interpreted in relation to both geopolymer chemistry and printing-induced features such as interlayer voids, anisotropic pore networks, surface moisture loss, weak interfaces, and direction-dependent transport pathways.

The third axis, sustainability and standardization, evaluates the environmental and practical implications of precursor selection, activator production, curing requirements, one-part geopolymer systems, waste-derived materials, local resource availability, LCA boundaries, and quality-control requirements. This axis also addresses standardization and implementation issues because reliable use of 3DPGPMs requires not only material-based performance criteria but also process-based qualification procedures for print quality, interlayer bonding, anisotropy, durability, and structural reliability.

In addition to thematic classification, the final selected reference set was descriptively examined in terms of publication year, source type, main research focus, and geographic origin where affiliation information was available. This descriptive mapping was used to identify the relative emphasis of the reviewed literature on mechanical performance, durability, sustainability, standardization, reinforcement strategies, and digital quality-control approaches. Regional distribution was considered as a contextual indicator rather than a comprehensive bibliometric ranking, because the practical relevance of 3DPGPM research may depend on local precursor availability, waste streams, activator supply chains, climatic exposure conditions, construction practices, and regulatory frameworks. Therefore, the literature classification adopted in this review provides not only a thematic structure for synthesis but also additional context for interpreting the transferability of reported findings.

The conceptual diagrams used in this review are intended to synthesize mechanisms, relationships, and performance pathways reported in the literature rather than to replace quantitative test data. They should therefore be interpreted as mechanism-oriented visual summaries, not as numerical performance charts or design limits. Wherever experimentally reported numerical data were available, representative values were incorporated into the corresponding mechanical, durability, sustainability, and implementation sections to support the conceptual interpretations. However, the diagrams were kept qualitative when the available studies did not provide directly comparable datasets, because reported values are strongly affected by binder chemistry, activator system, printing direction, specimen geometry, curing regime, exposure condition, and testing method.

**Figure 3:**
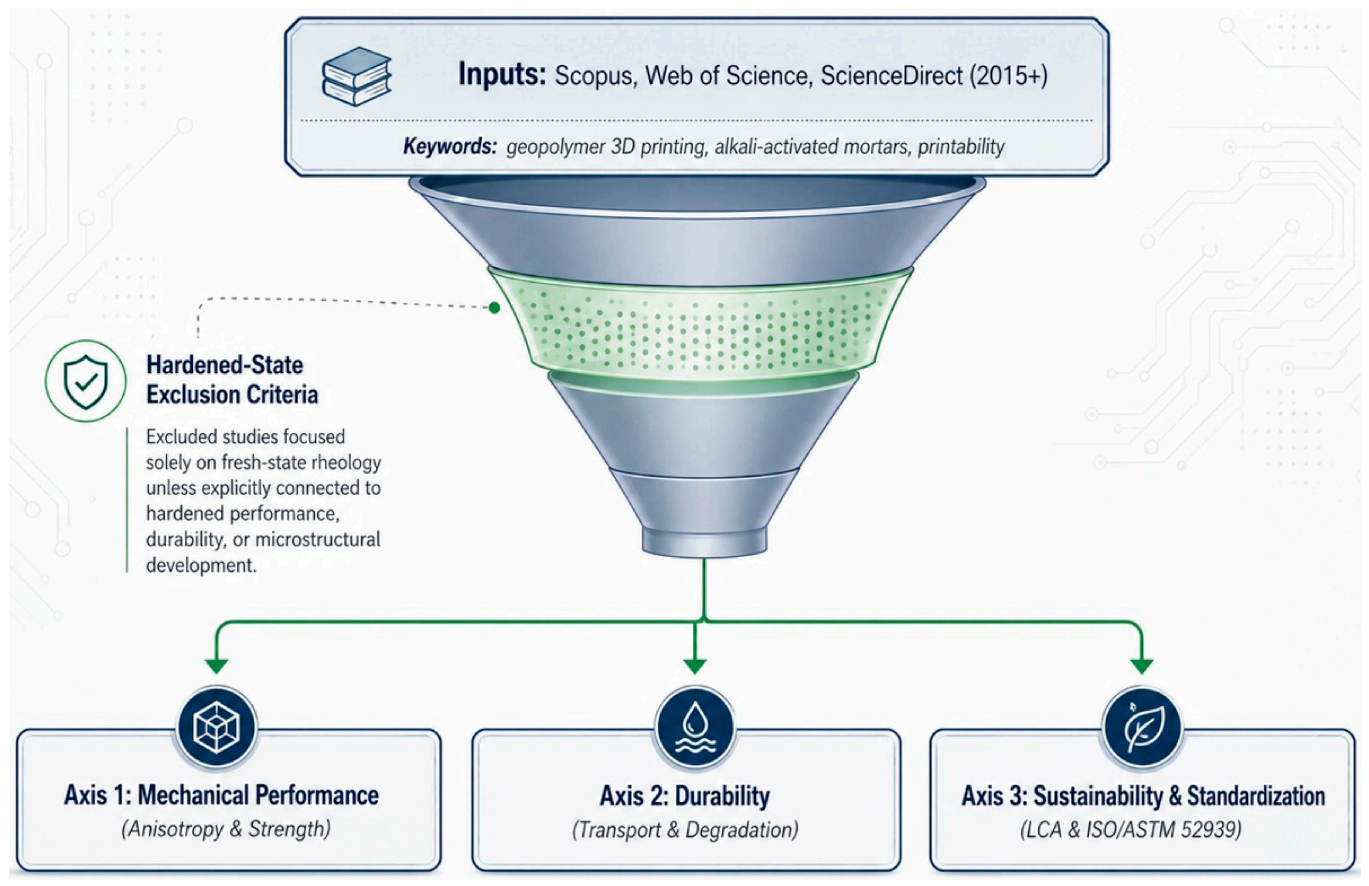
Data distillation funnel used to define the three review axes. Developed by the authors; standardization context informed by Roussel and Lowke [5] and ISO/ASTM 52939 [14].

**Figure 4:**
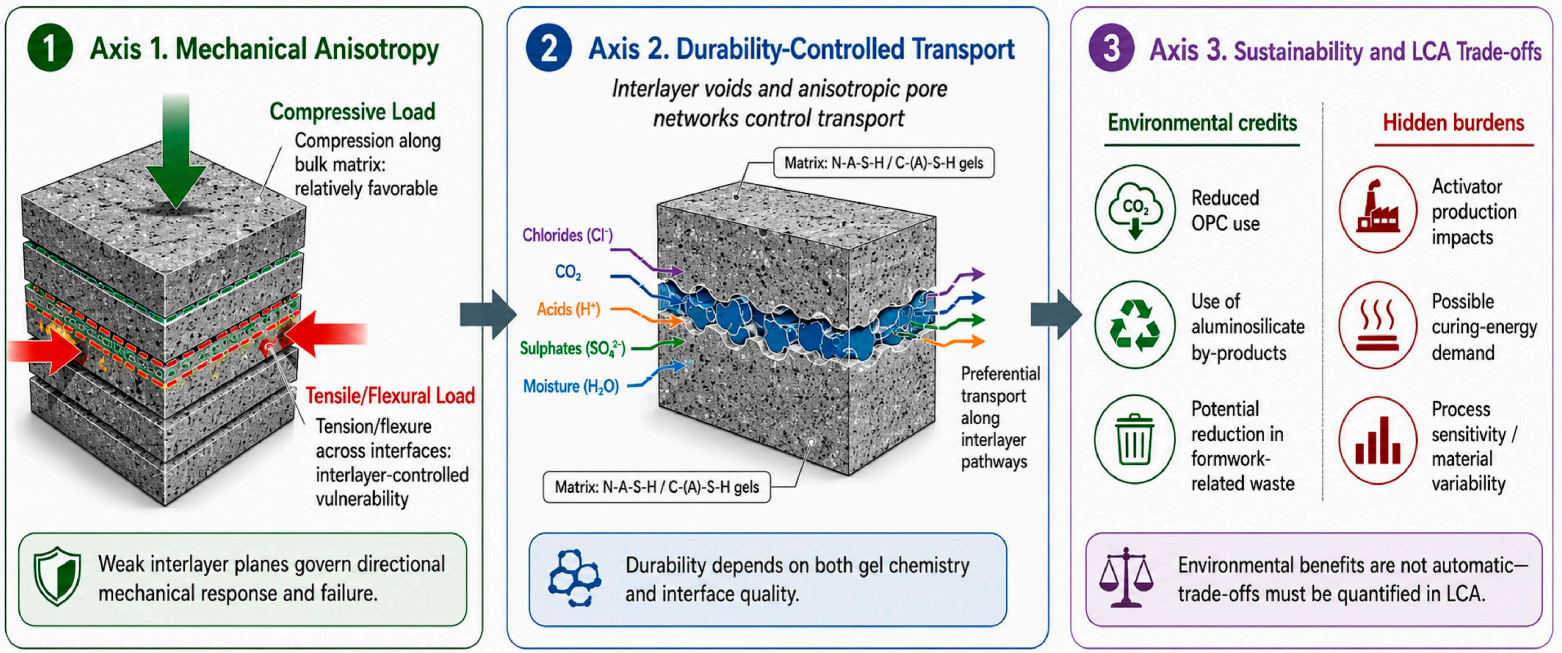
Integrated performance axes of 3DPGPMs. Conceptual synthesis based on selected literature [1,2,13,15,16,17,18,19,20,21].

Overall, this review framework connects material composition, rheology-related process parameters, printing conditions, mechanical performance, durability, environmental impact, and practical implementation within a single performance-oriented perspective. This structure enables the identification of the main scientific gaps and practical challenges that must be addressed before 3DPGPMs can be reliably used in durable and sustainable construction applications. The literature screening and synthesis workflow used to define the review axes is summarized in Figure 3, while the coupled performance logic among mechanical anisotropy, interface-controlled durability, and sustainability trade-offs is illustrated in Figure 4.

## 3. Materials, Mixture Design, and Printing-Related Parameters

### 3.1. Precursors and Activator Systems

The performance of 3DPGPMs depends strongly on precursor type, precursor reactivity, calcium content, particle characteristics, and activator compatibility. These parameters influence geopolymerization kinetics, setting behavior, pore structure, interlayer quality, and susceptibility to moisture or ion ingress [13]. Therefore, precursor and activator selection should not be evaluated only in terms of compressive strength, but also in relation to printability, open time, interlayer bonding, and long-term durability.

The slag/FA ratio is one of the key mixture-design parameters controlling the early-age behavior and hardened performance of printable geopolymer mixtures. GGBFS incorporation can increase calcium availability, accelerate geopolymerization, improve early-age structural build-up, and shorten setting time [22]. However, excessive slag content may reduce the available open time and narrow the printable time window. Therefore, the slag/FA ratio should be optimized to balance printability, setting behavior, interlayer bond development, and hardened mechanical performance [22,23].

The influence of precursor chemistry is also evident in MK-based 3D-printed geopolymer systems. Slag inclusion can promote the formation of C-S-H, N-A-S-H, and C-A-S-H type gels, leading to a denser matrix, refined pore structure, and improved mechanical performance [16]. These findings suggest that calcium-bearing precursors can enhance the hardened performance of 3DPGPMs when their effects on reaction rate, open time, printability, and interlayer bond quality are properly controlled.

### 3.2. Aggregate/Binder Ratio, Particle Packing, and Additives

Mixture proportioning in 3DPGPMs should be designed to balance printability, particle packing, matrix density, interlayer bonding, and hardened performance. Jaji et al. [16] showed that increasing the aggregate-to-binder ratio from 1.6 to 2.0 improved compressive, flexural, interlayer bond, and tensile performance, indicating that aggregate/binder ratio is a critical parameter in 3D-printed geopolymer mixture design. This suggests that optimized aggregate packing can contribute to a denser skeleton and improved stress transfer, provided that extrudability and layer continuity are not compromised.

Additives can further modify both fresh and hardened performance by influencing rheology, void structure, matrix densification, and crack development. For example, nano-graphite platelets have been reported to improve the flexural and compressive performance of 3D-printed geopolymer composites by increasing density, reducing voids, and limiting microcrack propagation, while also enhancing rheology, shape retention, and buildability [24]. These findings indicate that additive selection should be evaluated not only in terms of strength enhancement but also in relation to extrusion stability, layer continuity, interlayer bonding, and pore-structure refinement.

Pore-structure-modifying additives and fine mineral additions may contribute to hardened performance by improving matrix compactness and reducing connected porosity. However, their dosage should be optimized carefully because excessive modification may adversely affect rheology, extrudability, interlayer contact, or mechanical performance. Therefore, in 3DPGPMs, additives should be considered to be part of a coupled mixture–process design strategy rather than as isolated strength-enhancing components. Exposure-specific roles of additives, such as freeze–thaw resistance, acid resistance, and transport-related durability, are discussed in the relevant durability subsections.

### 3.3. Printing Parameters Affecting Hardened Performance

Printing parameters strongly influence the hardened performance of 3DPGPMs because they control filament geometry, interlayer contact, compaction, void formation, and direction-dependent mechanical behavior. Therefore, nozzle height, standoff distance, layer thickness, interlayer time interval, printing speed, and extrusion flow rate should be optimized together rather than treated as independent variables.

Among these parameters, the coordination between material flow rate and nozzle travel speed is particularly important for maintaining stable filament geometry. Archez et al. [25] showed that material flow and nozzle travel speed should be adjusted together to maintain a constant layer width, while lower printing speeds can reduce slump and improve layer stability. The same study also reported successful printing under controlled environmental conditions of 14–20 °C and 43–63% relative humidity, indicating that temperature and relative humidity can influence early-age stability and dimensional control in printable geopolymer systems.

Printable geopolymer mixtures may also be designed through process and formulation control without relying exclusively on conventional organic rheology-modifying agents. Archez et al. [25] reported mineral-only geopolymer formulations produced without organic viscosity modifiers, plasticizers, or setting agents, where printability was controlled through inorganic fillers, reinforcement phases, printing speed, and process optimization. This suggests that hardened performance should be interpreted as the result of coupled material–process interactions rather than mixture composition alone.

Compared with OPC-based printing systems, geopolymer systems may have a narrower process tolerance window because rheological evolution and early-age stiffening are highly sensitive to precursor chemistry, activator composition, environmental conditions, and process timing. If printing parameters are poorly controlled, variations in layer thickness, layer spacing, printing speed, extrusion flow, or nozzle geometry may increase cold-joint formation, interlayer porosity, reduced bond strength, and mechanical anisotropy. Therefore, printing parameters should be reported in detail and evaluated together with hardened mechanical and durability results.

### 3.4. Curing Regimes and Early-Age Development

Curing regime and early-age reaction development strongly influence the printability, buildability, interlayer bonding, and hardened performance of 3DPGPMs. This effect is particularly important for one-part or “just-add-water” geopolymer systems, where water addition activates the dry components and controls the development of setting, open time, structural build-up, and early mechanical performance. Bong et al. [26] showed that ambient-temperature cured one-part geopolymers can provide sufficient open time, buildability, shape retention, and mechanical performance for large-scale 3DCP applications, indicating the practical potential of ambient-curable geopolymer mixtures.

Thermal or accelerated curing can support early-age strength development, but its effect depends on precursor chemistry, activator composition, curing temperature, moisture condition, and printing method. Nematollahi et al. [27] used 24 h heat curing at 60 °C for 3D-printed fiber-reinforced geopolymer specimens, showing that accelerated thermal curing can be used to promote early-age performance in printable geopolymer systems. Microwave-assisted set-on-demand treatment can also accelerate early-age structural build-up and improve interlayer bond strength, although the heating duration must be optimized to avoid adverse effects such as excessive moisture loss or reduced interlayer quality [9].

Environmental curing conditions should also be controlled because temperature and relative humidity influence setting, strength development, moisture retention, dimensional stability, and interlayer bond formation. Inadequate control of temperature or humidity may promote premature drying, weak interlayer bonding, and reduced mechanical or durability performance; therefore, controlled printing and curing conditions are required for consistent buildability and hardened performance in geopolymer-based 3D printing systems [3,13,25]. Moisture-controlled curing may also help reduce porosity- and permeability-related durability risks, although this effect should be evaluated together with interlayer quality and chloride ingress behavior rather than treated as a matrix-only response [28].

The preceding subsections show that the hardened performance of 3DPGPMs is not governed by a single mixture parameter. Instead, it results from the combined effects of precursor reactivity, activator compatibility, early-age curing, fiber reinforcement, nano-additive modification, pore refinement, and interlayer bond development. Table 1 summarizes the key material-, curing-, and additive-related parameters that influence the printability and hardened performance of 3DPGPMs.

Overall, the reviewed studies indicate that the selection of raw materials and manufacturing parameters should be governed by a balance between printability, reaction kinetics, interlayer bond development, and hardened performance rather than by compressive strength alone. Calcium-rich precursors such as GGBFS can improve early-age structural build-up, setting, and matrix densification, but excessive slag content may shorten open time and reduce the printable time window. In contrast, FA- or MK-rich systems may offer longer workability but often require careful activator and curing control to achieve sufficient early-age stiffness and interlayer bonding. Quantitative evidence also shows that mixture proportioning is important; for example, increasing the aggregate-to-binder ratio from 1.6 to 2.0 improved compressive, flexural, tensile, and interlayer bond performance in a slag-modified MK-based 3D-printed geopolymer system [16]. From a process perspective, ambient curing is more suitable for field-scale and one-part geopolymer printing, whereas heat curing, such as 24 h at 60 °C, or microwave-assisted set-on-demand treatment may accelerate early strength and interlayer bond formation but should be controlled to avoid excessive moisture loss and interface weakening [9,26,27]. Therefore, the applicable scope of each raw material and manufacturing route should be defined by coupled criteria, including open time, extrudability, buildability, setting rate, interlayer bond strength, pore refinement, curing sensitivity, and long-term durability.

## 4. Mechanical Performance of 3DPGPMs

The mechanical performance of 3DPGPMs is governed by both material composition and the layer-induced structural characteristics formed during extrusion-based deposition [31]. For structural applications, particular attention should be given to interlayer bond strength because it is strongly affected by printing parameters and can control the mechanical reliability of printed layered elements [32]. Therefore, this section evaluates the hardened mechanical behavior of 3DPGPMs in terms of compressive strength, tensile and flexural performance, elastic modulus, interlayer bond strength, mechanical anisotropy, fiber reinforcement, and continuous reinforcement strategies [27,31,32]. In this section, direct 3DPGPM studies are prioritized; where OPC-based 3D-printed concrete or ECC-type systems are discussed, they are explicitly treated as transferable but indirect evidence for understanding printing-induced anisotropy, layer defects, and interface-controlled mechanical behavior.

### 4.1. Compressive Strength

Compressive strength is one of the fundamental mechanical properties of 3DPGPMs because it reflects the load-bearing capacity of the hardened matrix. In these systems, compressive strength is influenced by precursor composition, activator system, curing condition, pore structure, and printing-induced defects such as interlayer voids or weak layer contacts. Evidence from broader 3D-printable cementitious composites also shows that printed specimens may exhibit lower compressive strength than their mold-cast counterparts when small pores or discontinuities are introduced between printed layers; for example, Zhu et al. [30] reported that 3D-printed ECC specimens had compressive strength values 2–12% lower than mold-cast specimens. Therefore, compressive strength in 3DPGPMs should be interpreted together with layer quality, pore distribution, curing regime, and specimen orientation rather than as a matrix-only property.

### 4.2. Tensile and Flexural Strength

Geopolymer mortars generally exhibit brittle behavior under tensile and flexural loading, which limits their crack resistance and post-cracking load-carrying capacity. In 3D-printed geopolymer systems, this limitation becomes more critical because tensile and flexural performance may also be governed by weak interlayer interfaces, layer orientation, and printing-direction-dependent anisotropy. Short fiber reinforcement can improve tensile and flexural behavior by bridging cracks, enhancing post-cracking integrity, and reducing the severity of direction-dependent mechanical response. Panda et al. [29] showed that short fiber-reinforced 3DPGPMs exhibited direction-dependent tensile and flexural responses, indicating that fiber effectiveness should be evaluated together with loading direction, fiber distribution, and interlayer bond quality.

### 4.3. Elastic Modulus and Stress–Strain Behavior

The modulus of elasticity represents the stiffness of 3DPGPMs and their resistance to deformation under applied loading. In printed systems, stiffness and stress–strain response should not only be interpreted as matrix properties, because they may also be influenced by layer orientation, interlayer bond quality, pore structure, and printing-induced anisotropy. Studies on 3D-printed geopolymer composites have shown that loading direction and layer configuration can affect mechanical response, indicating the importance of evaluating stiffness together with anisotropy and failure mode [31]. Early-age treatment and curing strategy may further influence stiffness development by modifying geopolymerization, interlayer bonding, and microstructural densification [9,16]. Therefore, elastic modulus should be assessed together with compressive strength, interlayer bond strength, pore structure, and observed failure mechanisms to obtain a more reliable interpretation of the structural response of 3DPGPMs.

### 4.4. Interlayer Bond Strength and Failure Modes

**Figure 5:**
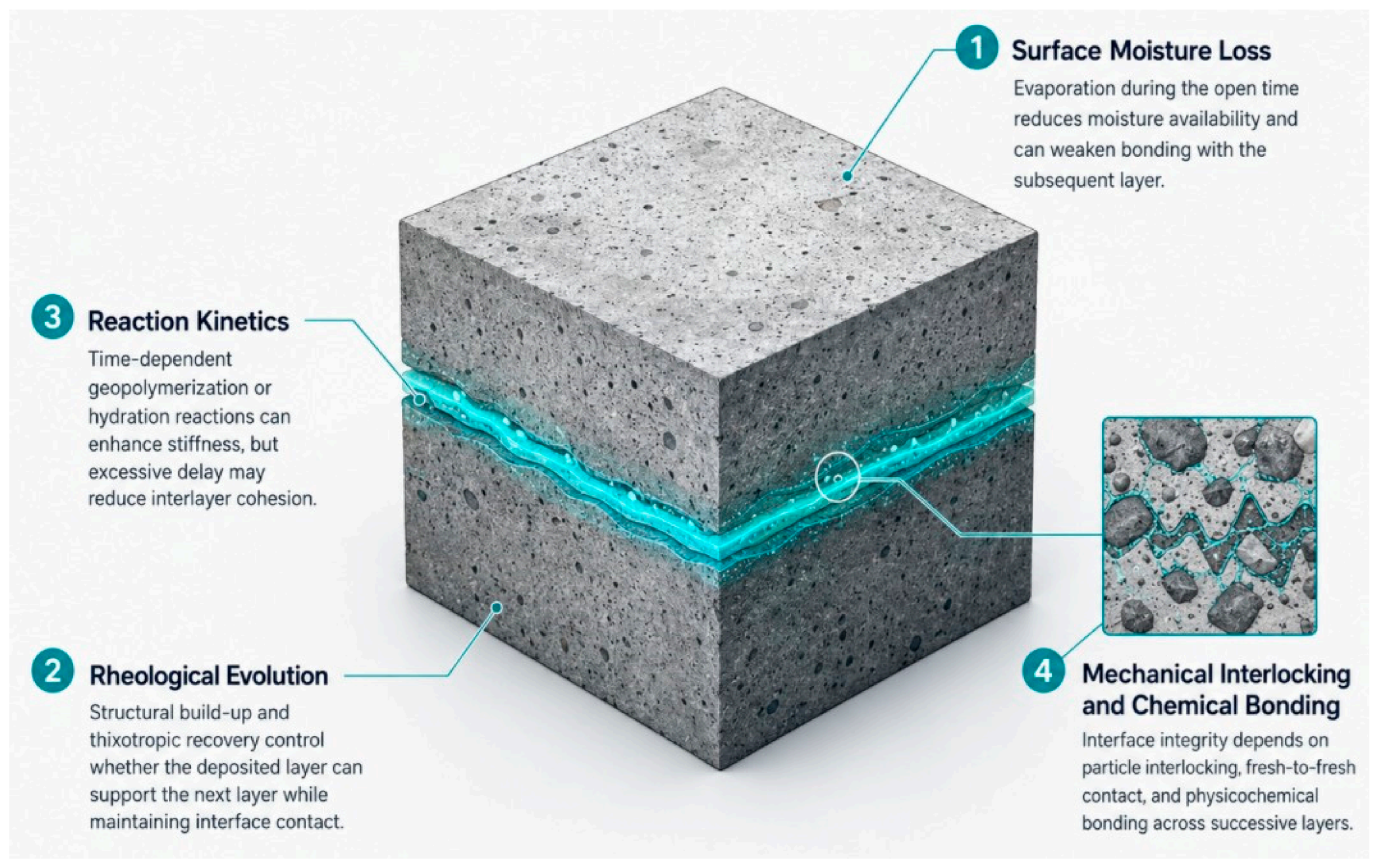
Anatomy of the layer interface in 3DPGPMs. Conceptual synthesis based on selected references [15,25,32].

Interlayer bond weakness is one of the main sources of anisotropic mechanical behavior in 3DPGPMs. As the time interval between successive layers increases, the previously deposited layer may undergo surface stiffening and moisture loss, reducing its ability to bond with the next layer [32]. This interface formation process is governed by the combined effects of surface moisture condition, rheological evolution, reaction kinetics, mechanical interlocking, and chemical bonding, as schematically illustrated in Figure 5. Weak interlayer regions can limit stress transfer between adjacent layers and reduce tensile or flexural strength, especially when loading is unfavorably oriented with respect to the printed layers [31].

Early-age curing and interlayer activation strategies may improve bond formation between successive layers when they enhance geopolymerization and mechanical interlocking at the interface. Muthukrishnan et al. [9] showed that microwave-assisted set-on-demand treatment can accelerate early-age structural build-up and improve interlayer bond strength; however, the activation intensity and duration should be optimized to avoid excessive moisture loss or unfavorable changes at the layer surface.

Interlayer bond strength values should be compared cautiously because they are strongly affected by testing configuration, printing time gap, nozzle speed, nozzle standoff distance, and the fresh-state condition of the interface material [32]. Since conventional compressive strength testing does not directly capture interlayer bond quality, direct tensile, shear, or fracture-based interlayer tests should be used together with failure-mode observations to evaluate the mechanical integrity of printed interfaces.

Excessive interlayer delay may affect not only mechanical bond strength but also durability-related transport behavior by increasing surface stiffening, moisture loss, and interfacial porosity. Therefore, open time should be considered a critical process parameter linking interlayer bond strength, failure mode, and the long-term performance of 3DPGPMs.

### 4.5. Mechanical Anisotropy Due to Layer Orientation

The layer-by-layer deposition process in extrusion-based 3D printing introduces direction-dependent mechanical behavior in 3DPGPMs. This anisotropy is mainly associated with layer configuration, interlayer quality, printing interval, and loading direction relative to the printed layers [1,31]. As a result, compressive, tensile, and flexural performance may vary depending on specimen orientation and loading direction, making anisotropy a critical design issue for the structural use of 3D-printed geopolymer systems [29].

**Figure 6:**
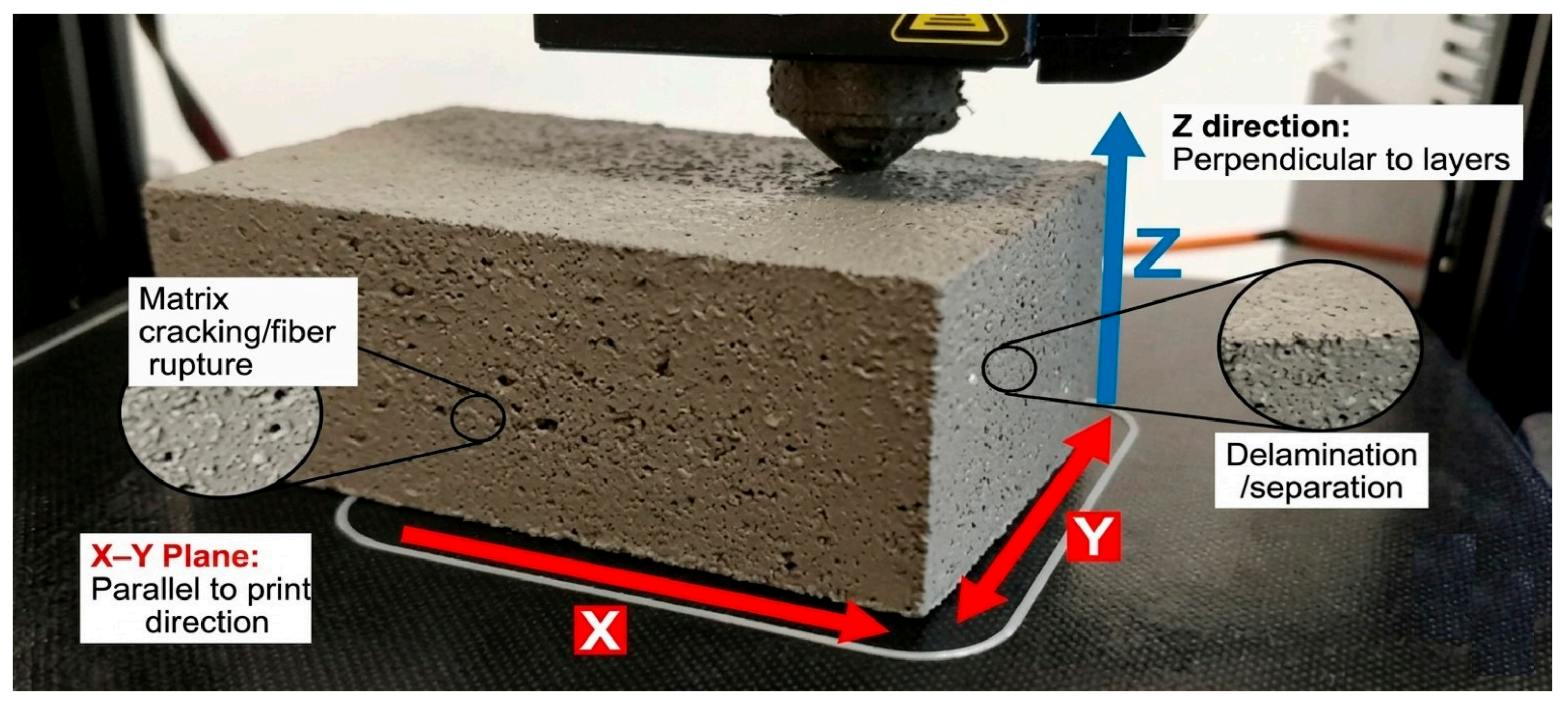
Schematic representation of layer orientation and direction-dependent failure in 3DPGPMs, showing the X–Y plane parallel to the printing direction, Z-direction loading perpendicular to the layers, and typical matrix cracking/fiber rupture and interlayer delamination mechanisms [1,31,33].

Mechanical anisotropy mainly originates from printing-induced interlayer interfaces and, in fiber-reinforced mixtures, from fiber distribution or orientation during extrusion. Weak interlayer regions can promote delamination or separation when stresses act perpendicular to the printed layers, whereas matrix cracking, fiber rupture, or fiber pull-out may dominate when loading is parallel to the printed layer plane [31]. In broader geopolymer 3D printing studies, compressive, flexural, and interlayer bond performance have also been shown to depend on printing direction, print interval, pore structure, and process parameters [1]. Figure 6 schematically illustrates the relationship between layer orientation, loading direction, and typical failure mechanisms in 3DPGPMs.

Table 2 summarizes the main direction-dependent failure mechanisms and typical mechanical responses of 3DPGPMs. This comparison highlights that specimens loaded parallel to the printed layer plane may benefit from filament continuity and effective fiber distribution, whereas specimens loaded perpendicular to the layers may be more vulnerable to weak interlayer bonding and delamination [1,31,33].

For this reason, reporting printing direction, loading direction, specimen extraction orientation, and interlayer time interval is essential for comparing mechanical test results of 3DPGPMs. Cube or cylinder compressive strength alone may not adequately represent the anisotropic behavior of printed materials, particularly when tensile, flexural, or interlayer failure modes are involved.

Mechanical anisotropy can be reduced by optimizing both material composition and printing parameters. Open time and printing interval should be adjusted according to setting behavior and structural build-up because they strongly affect layer adhesion and interlayer bond quality [1]. Experimental evidence also indicates that shorter time gaps and suitable layer configurations can improve flexural performance and reduce interlayer separation in layered geopolymer specimens [31]. Material-based strategies may also help by improving matrix cohesion, structural build-up, and interlayer quality. For example, nano-graphite platelets can improve shape retention, buildability, and mechanical performance in 3D-printed geopolymer composites [24], while the structuration rate in nano-clay-modified geopolymer mixtures should be optimized to avoid excessive build-up before subsequent layer deposition [34]. Ambient-curable “just-add-water” geopolymers may further support practical 3DCP by providing longer open time, good buildability, shape retention, and adequate mechanical performance [26].

Although mechanical anisotropy is repeatedly identified as a key feature of 3DPGPMs, directly comparable anisotropy ratios are not consistently available across the reviewed studies. This is mainly because different studies use different specimen geometries, loading directions, printing paths, layer orientations, interlayer intervals, curing regimes, and reinforcement configurations. Therefore, anisotropy should not be interpreted only from strength values, but also from the testing setup, specimen extraction direction, loading direction, interlayer condition, pore-defect distribution, and observed failure mode. Future studies should explicitly report the anisotropy definition used, such as the ratio between parallel-to-layer and perpendicular-to-layer strength, together with the corresponding test configuration, so that more reliable cross-study comparisons can be made.

Overall, mechanical anisotropy in 3DPGPMs should be evaluated as a combined function of layer orientation, interlayer bond quality, print interval, pore structure, fiber distribution, and loading direction. The absence of standardized bond and direction-dependent testing protocols still complicates the interpretation of reported anisotropic strength values, suggesting that future studies should clearly define specimen orientation, loading configuration, and failure mode when reporting mechanical performance.

### 4.6. Fiber Reinforcement and Fiber–Matrix Interface

Geopolymer mortars generally exhibit brittle behavior and limited tensile/flexural resistance; therefore, improving ductility, toughness, crack control, and post-cracking load-carrying capacity is important for structural and semi-structural applications [35]. Short fiber reinforcement is commonly used in 3DPGPMs to improve tensile and flexural performance, crack resistance, damage tolerance, and anisotropy-related mechanical response [29,35]. Fibers can bridge cracks and delay crack propagation, but their effectiveness depends on fiber type, dosage, dispersion, orientation, and compatibility with the geopolymer matrix [29,36].

In fiber-reinforced 3D-printed geopolymer composites, extrusion and layer-wise deposition can produce direction-dependent mechanical behavior. Panda et al. [29] showed that short glass fiber-reinforced 3DPGPMs exhibited anisotropic tensile, flexural, and compressive responses under different loading directions. In tensile loading, fiber distribution was more influential when loading was parallel to the printed path, whereas interlayer bond strength became more critical when loading was perpendicular to the printed layers [29]. Therefore, the tensile and flexural performance of fiber-reinforced printed geopolymers should be interpreted together with fiber orientation, loading direction, interlayer bond quality, and failure mode.

Fiber type, dosage, and geometry strongly influence the mechanical response of geopolymer composites. Korniejenko et al. [37] showed that 0.5–2.0% long fiber reinforcement can affect flexural strength, fracture behavior, crack propagation, and ductility in geopolymer composites intended for additive manufacturing. In 3DPGPMs, glass fibers have been directly used to improve tensile and flexural performance, although their efficiency depends on fiber content, fiber length, dispersion, fiber–matrix bond, and loading direction [29,35]. Synthetic fibers such as PP, PVA, and PE may improve crack control, toughness, shrinkage control, and post-cracking behavior, but evidence from broader ECC or cementitious systems should be interpreted cautiously when transferred to geopolymer matrices [30,35]. Basalt and other mineral or natural fibers may also offer potential benefits in terms of crack control, toughness, sustainability, and thermal stability, although their long-term durability, alkali resistance, dispersion, and matrix compatibility should be verified for each geopolymer system [35].

Table 3 summarizes the main short fiber types used or proposed for 3DPGPMs and their expected mechanical contributions. The table emphasizes that fiber selection should not be based only on tensile or flexural strength improvement, but also on printability, extrusion stability, fiber dispersion, fiber–matrix compatibility, and durability under alkaline exposure conditions.

The effectiveness of fiber reinforcement depends strongly on the fiber–matrix interface. A strong interface improves stress transfer, crack bridging, pull-out resistance, toughness, and energy absorption, whereas a weak interface may lead to premature debonding or fiber pull-out [29]. The quality of this interface is influenced by matrix porosity, fiber distribution, surface characteristics of the fiber, and chemical or mechanical compatibility between the fiber and the alkaline geopolymer matrix [35].

Fiber-related performance can also be supported by mixture and process strategies that improve matrix cohesion, interlayer quality, and early-age structural build-up. Nano-graphite platelets have been shown to improve mechanical performance, shape retention, and buildability in 3D-printed geopolymers [24]. For nano-clay-modified geopolymer mixtures, the structuration rate should remain within an optimum printing window because excessive build-up before subsequent layer deposition may weaken interlayer bonding [34]. Microwave-assisted set-on-demand treatment can also enhance interlayer bond strength and buildability by accelerating geopolymerization and improving mechanical interlocking between printed layers [9]. These strategies suggest that fiber reinforcement should be optimized together with mixture rheology, interlayer timing, and early-age bond development rather than treated as an isolated strengthening mechanism.

### 4.7. Continuous Reinforcement and Structural Element Performance

Short fiber reinforcement can improve ductility, crack control, and post-cracking behavior in 3D-printed geopolymer composites; however, it may not be sufficient to overcome the load-bearing limitations associated with weak interlayer bonding, mechanical anisotropy, and scale effects in structural elements [1]. Therefore, continuous or in-process reinforcement systems have been explored to enhance tensile resistance, flexural capacity, ductility, and structural reliability in extrusion-based geopolymer printing [15].

In-process reinforcement strategies allow steel cables or micro-cables to be integrated into the geopolymer filament during extrusion, enabling reinforcement placement to occur simultaneously with printing [38,39]. Such systems can improve load transfer across the printed matrix and provide post-cracking resistance beyond the contribution of short fibers alone. Lim et al. [38] and Li et al. [40] showed that cable- or micro-cable-reinforced geopolymer composites can exhibit improved flexural strength, toughness, load-bearing capacity, deformation capacity, and post-cracking behavior compared with unreinforced printed specimens.

The effectiveness of continuous reinforcement depends strongly on print path, reinforcement configuration, anchorage, embedment quality, and matrix–reinforcement compatibility. Li et al. [40] reported that steel micro-cable-reinforced geopolymer specimens showed more ductile behavior under different loading conditions and retained load-carrying capacity after cracking through stress transfer from the cracked geopolymer matrix to the micro-cables. However, reinforcement integration may also introduce practical challenges, including cable slippage, poor embedment, nozzle-integration difficulties, and discontinuous load transfer if the reinforcement is not properly positioned within the printed filament.

Post-process reinforcement is another possible strategy, in which bars, cables, or other tensile elements are placed into preformed voids, channels, or printed formwork after printing and then bonded using grout, concrete, or compatible filling materials. This concept has been discussed in 3D concrete printing through printed permanent formwork and post-placement reinforcement strategies [38,40]. However, its structural efficiency should be evaluated cautiously because it depends on reinforcement anchorage, grout compatibility, interface quality, and the continuity of load transfer between the printed geopolymer matrix and the reinforcement system.

Although continuous reinforcement can improve the mechanical performance of 3D-printed geopolymer elements, it does not eliminate the anisotropic effects associated with layer orientation, print path, void distribution, and weak interfaces. Therefore, structural design and performance assessment should explicitly consider loading direction, interlayer bond quality, reinforcement placement, anchorage, matrix–reinforcement compatibility, and failure mode [1]. In addition, long-term performance should not be assessed only through flexural capacity and post-cracking response; reinforcement durability under carbonation, chloride exposure, moisture transport, and possible interface degradation should also be investigated in future structural-scale studies.

To provide a clearer quantitative perspective, selected numerical findings were added to support the above comparison. Reported evidence from broader 3D-printable cementitious composites indicates that printed specimens may show approximately 2–12% lower compressive strength than mold-cast specimens when interlayer pores or discontinuities are introduced [30]. For geopolymer-based printed systems, available quantitative results also show that performance improvements are strongly linked to pore refinement and optimized additive dosage. For example, 0.5% nano-ZnO in MK–FA-based 3DPGPMs produced 33.64% higher residual compressive strength and 42.86% higher residual flexural strength than the acid-exposed reference mixture after 5% HCl exposure [41], while another MK–FA-based 3DPGPM containing 0.5% nano-ZnO showed 95.72% compressive strength retention after freeze–thaw exposure [42]. In hybrid concrete–geopolymer systems, compressive strength reduction after 150 freeze–thaw cycles reached up to 27% in molded specimens, whereas the decrease in 3D-printed specimens did not exceed 12% [43]. These values indicate that the most meaningful improvements are often associated with interlayer quality, pore refinement, additive optimization, and exposure-dependent residual performance rather than compressive strength alone; however, they should be interpreted as study-specific findings rather than universal performance limits.

In cross-comparison, the macroscopic mechanical performance of 3DPGPMs is controlled by the interaction between matrix strength, pore structure, interlayer bond quality, loading direction, and reinforcement strategy. Compressive strength is mainly influenced by matrix density and printing-induced voids, whereas tensile and flexural behavior are more sensitive to weak layer interfaces, fiber orientation, and loading direction. Evidence from broader 3D-printable cementitious composites shows that printed specimens can exhibit compressive strength values approximately 2–12% lower than mold-cast specimens when interlayer pores or discontinuities are introduced [30]. For 3DPGPMs, interlayer bond strength is particularly governed by printing time gap, nozzle speed, nozzle standoff distance, surface moisture condition, and rheological build-up [32]. Short fibers are effective for crack bridging, toughness, and post-cracking resistance, whereas continuous steel cable or micro-cable reinforcement is more relevant when higher flexural capacity, deformation capacity, and structural load transfer are required [38,39,40]. These findings indicate that mechanical regulation rules for 3DPGPMs should not rely only on matrix compressive strength; instead, they should include direction-dependent testing, interlayer bond evaluation, pore-defect assessment, reinforcement continuity, and failure-mode identification.

These representative numerical findings were included to support the conceptual interpretation of mechanical and durability-related mechanisms, while avoiding unsupported quantitative generalization across non-identical material systems and testing configurations.

## 5. Durability Performance

The durability performance of 3DPGPMs is controlled by the combined effects of geopolymer binder chemistry and printing-induced structural features. While precursor composition, activator chemistry, geopolymerization products, and pore structure govern the intrinsic resistance of the matrix, extrusion-based layer deposition introduces additional durability-sensitive features such as interlayer interfaces, anisotropic pore networks, weak layer contacts, and direction-dependent transport pathways. Printing configuration and process parameters, including nozzle design, printing speed, and print interval, can influence porosity, layer geometry, interlayer bond quality, and structural integrity in 3D-printed geopolymers [1,15]. Similarly, durability findings from OPC-based printed cementitious systems are used only as transferable evidence for interpreting layer-induced transport pathways, interlayer defects, and testing-direction effects, whereas conclusions on 3DPGPM durability are based primarily on geopolymer-specific or alkali-activated evidence when available.

**Figure 7:**
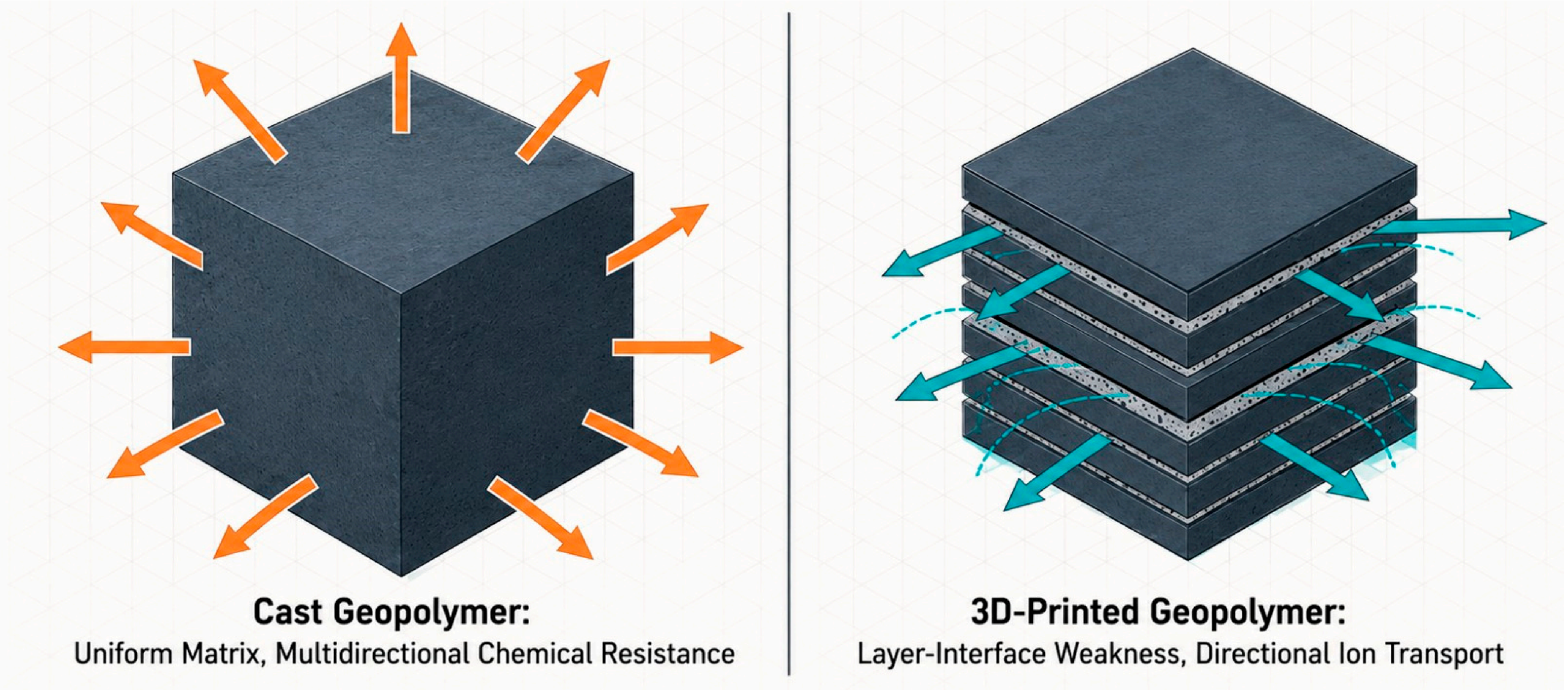
Durability paradigm shift from cast to 3D-printed geopolymer systems. The orange arrows indicate multidirectional chemical resistance in the cast geopolymer, whereas the solid turquoise arrows represent directional ion transport in the 3D-printed geopolymer. The dashed turquoise lines indicate preferential transport pathways along the interlayer interfaces. Conceptual synthesis based on Zhang et al. [11], Yang et al. [12], Zhong and Zhang [15], and Barve et al. [1].

For this reason, durability should not be interpreted only as a matrix-level material property. In 3DPGPMs, anisotropy, interlayer voids, interlayer bond quality, pore connectivity, and printing direction may affect the ingress and transport of water, gases, and aggressive ions. These factors can influence shrinkage-induced cracking, water absorption, permeability, chloride penetration, carbonation, acid and sulphate attack, freeze–thaw damage, and elevated-temperature performance. Therefore, durability assessment should consider both the chemical resistance of the geopolymer binder and the process-dependent microstructural features created by layer-by-layer deposition. This distinction between cast and 3D-printed geopolymer durability is conceptually illustrated in Figure 7.

The practical implementation of 3DPGPMs depends not only on printability and mechanical strength but also on their long-term performance under realistic service environments. Although geopolymer binders may offer potential advantages under certain chemical or thermal exposures, this potential should be evaluated cautiously in printed systems because interlayer interfaces and anisotropic pore structures may become preferential pathways for moisture and aggressive agents. Therefore, long-term durability testing is essential for advancing 3DPGPMs from laboratory-scale mixtures toward reliable construction materials.

Representative studies reporting the influence of mixture composition, printing method, and additives on durability-related performance are summarized in Table 4. These studies indicate that durability may be improved through pore refinement, matrix densification, optimized slag or nano-additive incorporation, and improved interlayer quality; however, the reported benefits remain strongly dependent on binder system, printing parameters, curing condition, and exposure type.

### 5.1. Shrinkage and Cracking

Shrinkage and cracking are important durability-related concerns in 3D-printed cementitious materials, including geopolymer mortars. The absence of formwork exposes printed layers directly to evaporation, which can increase the risk of plastic shrinkage cracking, surface drying, and interlayer slippage [28]. In geopolymer printing, interlayer moisture transfer, drying, dehydration, and shrinkage may also influence interlayer bond development and mechanical integrity [44]. Therefore, shrinkage and cracking should be evaluated not only as matrix-level phenomena but also as process-dependent responses affected by layer exposure, moisture condition, curing regime, and interlayer quality.

Drying shrinkage in 3DPGPMs is strongly affected by precursor selection, activator content, and curing conditions. Mishra et al. [13] noted that high-volume FA or FA–GGBS systems may be more favorable in terms of drying shrinkage, whereas high-GGBS and MK-based mixtures may show greater shrinkage sensitivity. Since inadequate control of temperature and relative humidity during and after printing can promote premature drying, weak interlayer bonding, and reduced strength or durability, shrinkage mitigation should rely on optimized mix design, controlled curing, suitable precursor reactivity, and carefully selected admixtures or fiber reinforcement.

Additives with moisture-retention or pore-refining potential may help improve the cracking resistance of printed geopolymer systems, but their role should be interpreted cautiously when direct shrinkage data are limited. Nan et al. [45] reviewed that 1–2 wt% halloysite nanotubes improved rheology and buildability in 3DPGPMs, while 3% halloysite nanotube reduced internal porosity and increased Z-direction compressive and flexural strength by more than 39%, together with a 19% improvement in Y-direction interlayer bond strength. These findings suggest that halloysite nanotubes may indirectly contribute to shrinkage-related performance by improving moisture-related behavior, porosity, and interlayer quality; however, their internal-curing or shrinkage-mitigation effect still requires direct validation.

Fiber reinforcement can also contribute to crack control, but its effect should be distinguished from direct shrinkage reduction. Nematollahi et al. [27] showed that polypropylene fiber addition improved shape-retention ability, ductility, fracture energy, and crack-bridging behavior under flexural loading in 3DPGPMs. However, this evidence should not be used to claim that PP fibers directly eliminated shrinkage cracks or reduced cracking at layer interfaces, because shrinkage cracking was not the main focus of that study and higher fiber dosages reduced interlayer bond strength. Therefore, fiber-based crack-control strategies should be optimized together with printability, interlayer bond quality, and curing conditions.

### 5.2. Water Absorption, Sorptivity, and Permeability

Water absorption, sorptivity, oxygen permeability, and related transport indicators are critical for evaluating the durability of 3DPGPMs because they control the movement of water, oxygen, CO_2_, and aggressive ions through the hardened matrix. In these systems, transport-related durability depends not only on precursor chemistry and matrix density but also on printing-induced pore structure, interlayer voids, and direction-dependent transport pathways.

The influence of precursor chemistry on permeability-related behavior has been reported in MK-based 3D-printed geopolymer systems. As summarized by Jaji et al. [16], previous work showed that 5% slag inclusion improved anisotropic permeability behavior, evaluated through water absorption and oxygen permeability index, in MK-based 3DPGPC. In the same research line, Jaji et al. [16] further showed that increasing slag content up to 30% promoted the formation of C-S-H, N-A-S-H, and C-A-S-H gels, leading to pore refinement, matrix densification, and improved mechanical performance. These findings indicate that calcium incorporation can improve matrix-level transport resistance when it contributes to denser gel formation and reduced pore connectivity.

However, transport in 3DPGPMs should not be interpreted from matrix densification alone. Even when an optimized mixture produces a compact matrix, localized interlayer voids, weak layer bonding, and spatially heterogeneous pore distribution may create preferential pathways for water, gases, or aggressive ions along layer boundaries. Mishra et al. [13] emphasized that pores in interlayer regions can facilitate chemical ion ingress and increase chloride-related durability risks. Therefore, permeability and water absorption tests should consider specimen orientation, pore connectivity, spatial pore distribution, and layer-interface quality, rather than relying only on total porosity or bulk matrix density.

From a durability-design perspective, low connected porosity is also important for limiting the ingress of sulphate-bearing or other aggressive solutions. However, in 3DPGPMs, this resistance may be controlled not only by binder chemistry but also by interlayer transport pathways. Therefore, water absorption, sorptivity, and permeability should be evaluated together with interlayer quality and direction-dependent exposure conditions.

### 5.3. Chloride Penetration

Chloride penetration is a critical durability concern for 3D-printed cementitious and geopolymer-based systems, particularly when printed elements are intended for reinforced or marine-exposed applications. In reinforced systems, chloride ingress may initiate corrosion once chloride ions reach embedded reinforcement. In 3D-printed materials, this risk should not be evaluated only through matrix density, because interlayer voids, weak interfaces, discontinuous pore networks, and direction-dependent transport pathways may increase chloride ingress compared with well-consolidated cast materials.

**Figure 8:**
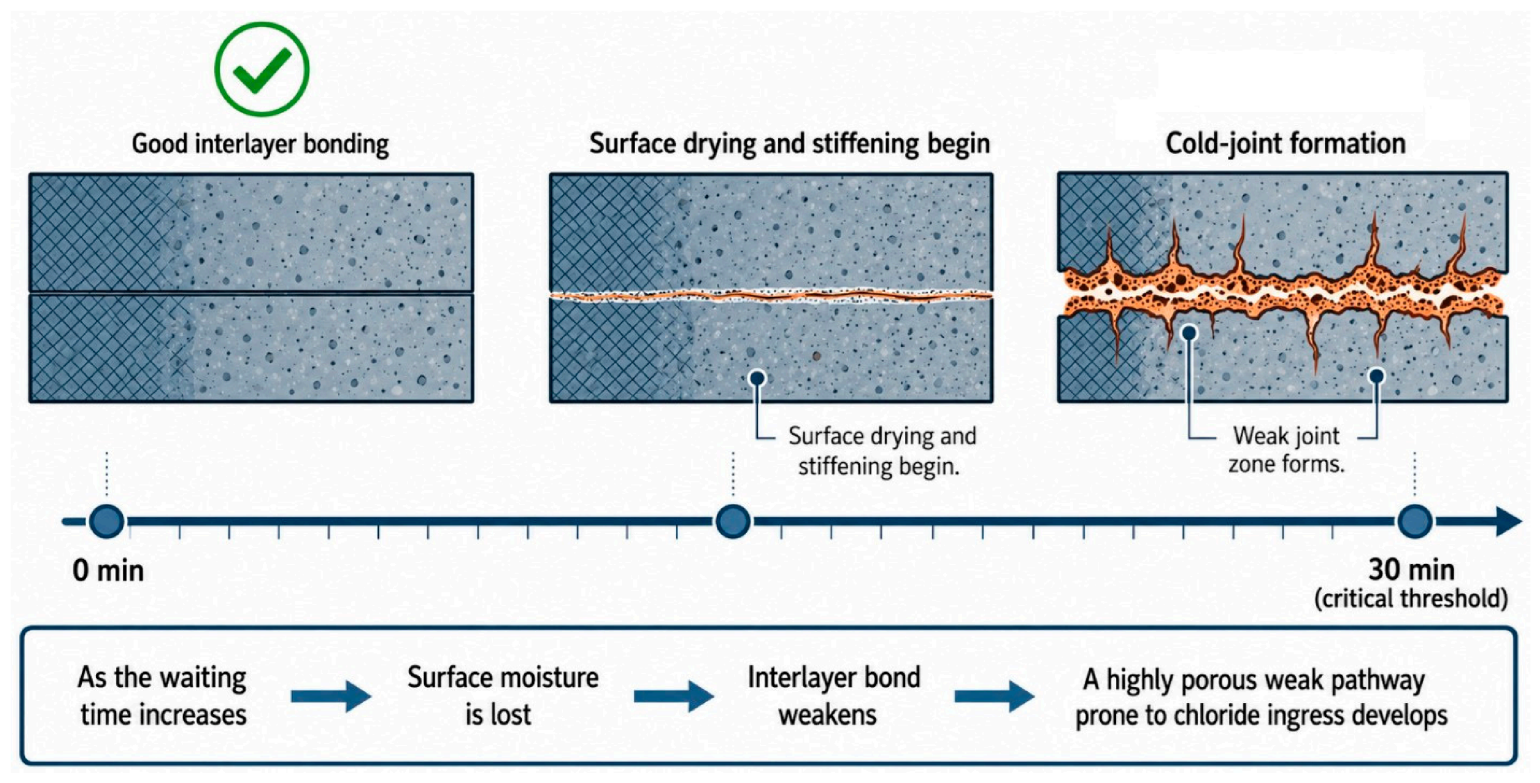
Effect of interlayer waiting time on cold-joint formation and chloride-ingress pathways. Conceptual synthesis based on Kruger et al. [46], Van der Putten et al. [47], and Mishra et al. [13].

Printing-induced pore structure provides an important basis for understanding chloride transport in layered systems. Kruger et al. [46] showed that extrusion-based 3D-printed concrete can exhibit spatially heterogeneous porosity with distinct intra- and interlayer void characteristics, which may create preferential transport paths along layer regions. Van der Putten et al. [47] further demonstrated that increasing the time gap between successive layers increased chloride penetration, with more pronounced ingress along interlayer regions. This time-dependent transition from good interlayer bonding to cold-joint formation and chloride-prone weak pathways is schematically illustrated in Figure 8. Therefore, chloride durability assessment of 3DPGPMs should explicitly consider interlayer quality, printing interval, specimen orientation, and transport direction.

For 3D-printed geopolymer systems, precursor chemistry and matrix densification should be evaluated together with interlayer quality. Mishra et al. [13] noted that printed geopolymer specimens may be sensitive to chloride penetration because pores and weak regions can exist along interlayer zones, and increasing the print time gap can reduce interlayer bond quality. At the matrix level, Jaji et al. [16] showed that slag-modified MK-based 3DPGPC developed denser microstructure, refined pore structure, and improved interlayer bond strength, while also noting earlier evidence on anisotropic permeability behavior in MK-based 3DPGPC. These findings suggest that mixture optimization may reduce transport-related vulnerability, but chloride resistance should still be interpreted in relation to spatial pore distribution, interlayer continuity, and direction-dependent transport behavior.

Direct standardized chloride diffusion or migration data for 3DPGPMs remain limited. Therefore, current interpretations are often supported by indirect transport indicators such as water absorption, sorptivity, oxygen permeability, and pore-structure measurements. However, these indicators should not be treated as direct substitutes for chloride diffusion or migration testing, because chloride transport also depends on pore solution chemistry, chloride binding capacity, gel structure, calcium content, pore connectivity, and interlayer continuity. Bradshaw et al. [28] reviewed that chloride resistance in printed cementitious systems is strongly affected by material composition and curing regime, and that optimized mixes containing SCMs such as GGBFS, MK, and nano-silica can reduce chloride permeability. The same review also indicated that high-relative-humidity curing may improve chloride resistance, supporting the importance of moisture-controlled curing and pore-refining strategies for limiting transport-related durability risks.

**Figure 9:**
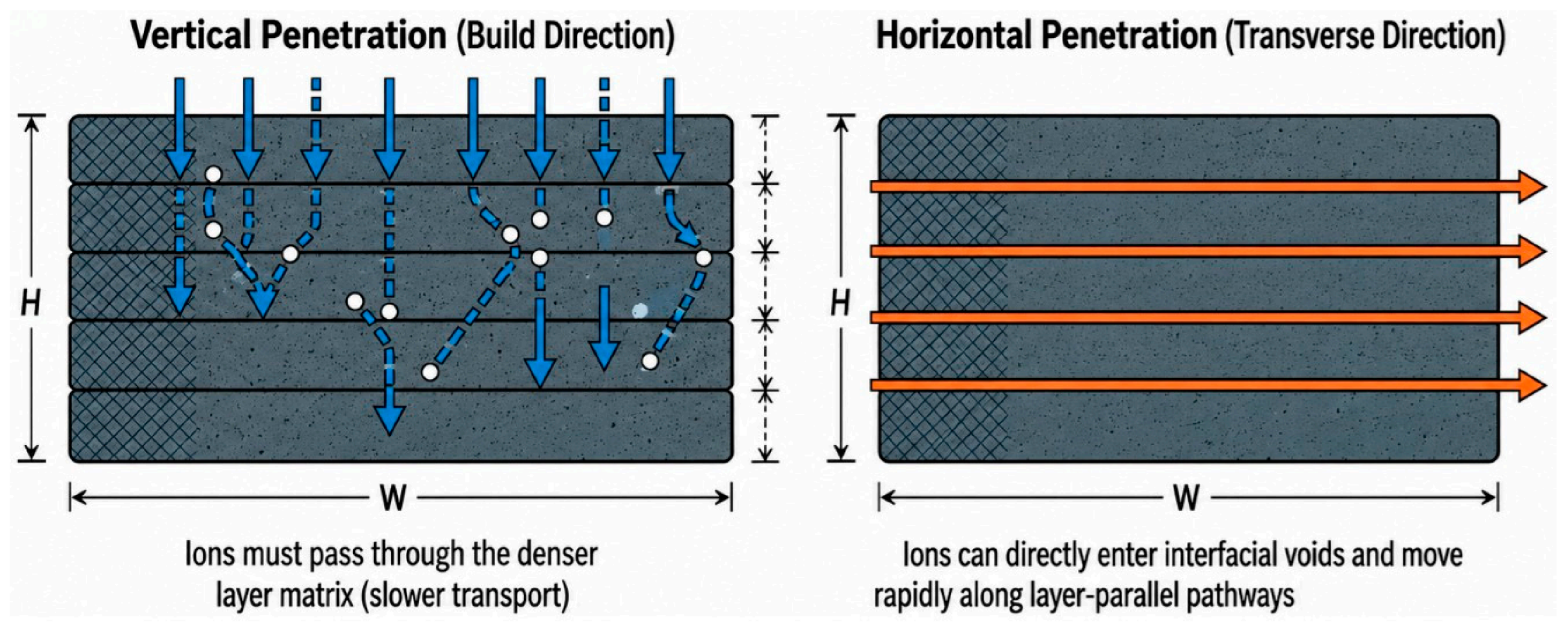
Direction-dependent chloride penetration in layered 3D-printed cementitious systems. The solid arrows indicate the principal directions of chloride penetration, whereas the dashed arrows represent tortuous transport pathways through pores and interlayer defects. The cross-hatched background regions represent the denser printed-layer matrix. Conceptual synthesis based on Min et al. [48].

Direction-dependent chloride transport should also be considered when interpreting test results. Min et al. [48] reported that, in binder-jetting 3D-printed cementitious mortars, the chloride diffusion coefficient measured in the transverse direction was approximately 186.1–407.1% higher than that measured in the build direction. This increase was attributed to gaps between printed layers that facilitated chloride penetration when aligned with the diffusion direction. The directional difference between build-direction and transverse chloride penetration is schematically shown in Figure 9. Although this evidence is based on binder-jetting cementitious systems rather than extrusion-based geopolymer mortars, it highlights the need to clearly report printing direction, specimen extraction orientation, exposure surface, and testing direction in chloride-resistance studies.

Available evidence from broader 3D-printed cementitious systems also confirms that interlayer defects and matrix compactness are important for chloride resistance. Chen et al. [49] showed that defects typically occur between printed layers and can significantly affect durability; in their slag-modified 3D-printed calcium sulphoaluminate cement-based materials, increasing slag powder content up to 10% reduced the chloride migration coefficient by improving compactness and delaying chloride ion transport. Although this evidence is not geopolymer-specific, it supports the general interpretation that minimizing interlayer defects and achieving a dense printed matrix are essential for improving chloride resistance.

Overall, chloride resistance in 3DPGPMs should be evaluated as a coupled function of matrix pore refinement, precursor chemistry, curing regime, printing interval, interlayer quality, pore connectivity, and transport direction. Long-term corrosion performance of reinforced 3D-printed geopolymer elements remains insufficiently documented and should be validated through dedicated chloride migration, carbonation, pore solution alkalinity, chloride binding, and reinforcement corrosion tests rather than inferred only from pore-structure or water-transport indicators.

### 5.4. Carbonation

Carbonation is an important durability mechanism for 3DPGPMs, particularly when printed elements are reinforced or exposed to combined CO_2_, moisture, and chloride actions. In geopolymer and alkali-activated systems, carbonation behavior may differ from OPC-based materials because pore solution chemistry, alkali reserve, calcium availability, reaction products, and pore structure are different. Although a dense geopolymer matrix may limit CO_2_ ingress, the lower availability of portlandite compared with OPC systems may reduce pH-buffering capacity and modify the carbonation mechanism. Therefore, carbonation in 3DPGPMs should be evaluated not only as a matrix-level chemical process but also as a durability mechanism that may affect pore solution alkalinity, reinforcement passivation, efflorescence behavior, and long-term transport resistance.

In 3D-printed systems, carbonation resistance is strongly influenced by printing-induced microstructural features. Bradshaw et al. [28] noted that carbonation resistance in 3D-printed concrete is affected by printing parameters, material composition, curing regime, permeability, interfacial porosity, anisotropic pore networks, and shrinkage-induced cracks at layer interfaces. Similarly, Rehman and Kim [6] emphasized that voids and cracks may occur at printed layer interfaces and that the influence of porosity, shrinkage cracks, printing parameters, and carbonation on durability requires further investigation. These findings suggest that layer interfaces, cold-joint-like regions, connected pores, and interlayer microcracks may act as preferential CO_2_ transport pathways in 3DPGPMs.

Direction-dependent carbonation has been confirmed in recent studies on 3D-printed cement-based materials. Zhang et al. [50] reported that printed specimens may exhibit greater carbonation depth than cast counterparts because layer-wise geometry, anisotropic pore structure, and interlayer defects can facilitate CO_2_ transport. The same study showed that carbonation depth and carbonation rate measured parallel to the printed filaments were generally higher than those measured perpendicular to the filaments or in cast samples, confirming the direction-dependent nature of carbonation in printed systems [50]. In addition, Zhang et al. [50] recommended measuring the maximum carbonation depth near cold-joint regions, because mean carbonation depth alone may not adequately capture localized carbonation ingress along weak interfaces.

Direct carbonation data for 3DPGPMs remain limited; therefore, indirect evidence should be used cautiously. Mishra et al. [13] emphasized that long-term durability data for 3D-printed geopolymer concrete remain insufficient, particularly under aggressive exposure conditions such as salts, acids, freeze–thaw cycles, chloride penetration, and carbonation-related environments. This limitation means that conclusions on carbonation rate, pH-buffering capacity, efflorescence formation, strength evolution, or reinforcement corrosion in 3DPGPMs should be supported by direct geopolymer-specific carbonation tests rather than inferred only from OPC-based printed systems.

Evidence from geopolymer systems produced by other additive manufacturing routes can still provide useful mechanistic insight. Posani et al. [51] investigated a binder-jet 3D-printed MK-based geopolymer composite and reported that one-year-old naturally aged samples reached mass stability rapidly during accelerated carbonation, indicating that they were already close to full carbonation. TGA-MS results confirmed CO_2_ release associated with carbonate products in the geopolymer matrix, while carbonation was reported to consume free alkalis and potentially reduce efflorescence risk [51]. However, the same study also noted that carbonation may decrease moisture-buffering capacity by reducing free alkali activators and modifying the material [51]. Since this evidence comes from binder-jet MK-based geopolymer composites, it should be interpreted as mechanistic support rather than direct evidence for extrusion-based 3DPGPMs.

Overall, the carbonation resistance of 3DPGPMs remains insufficiently investigated. Potential advantages associated with dense geopolymer matrices may be reduced when layer-induced diffusion pathways, interlayer microcracks, connected pores, or cold-joint regions facilitate CO_2_ ingress. Future studies should therefore conduct both accelerated and natural carbonation tests on 3D-printed geopolymer specimens with different printing directions, interlayer time intervals, curing regimes, precursor compositions, and exposure surfaces. Such studies should quantify carbonation depth, maximum carbonation depth near interlayer regions, pore solution alkalinity, carbonate product formation, moisture-buffering capacity, and possible reinforcement corrosion risk. These data are necessary for developing protective design strategies, including surface treatments, coatings, pore-refining admixtures, curing optimization, and interface-improving methods.

### 5.5. Acid Attack

Geopolymer and alkali-activated materials are often considered promising for acidic environments, particularly when their calcium content is relatively low. This behavior is generally associated with the limited availability of portlandite and the formation of aluminosilicate-based gel phases, which may be less vulnerable to acid-induced decalcification than conventional OPC hydration products. Ricciotti et al. [52] noted that geopolymer materials exposed to 5% sulfuric acid or hydrochloric acid solutions can show substantially lower degradation rates than Portland cement systems under comparable conditions. However, under strong acidic exposure, especially at very low pH, geopolymer gels may also dissolve, degrade, or lose mechanical integrity depending on precursor chemistry, pore structure, acid type, concentration, and exposure duration.

In 3D-printed systems, acid resistance should be evaluated not only as a binder-related property but also as a process-dependent durability response. Interlayer regions, connected pores, shrinkage-induced microcracks, and elongated printing-related voids may act as preferential pathways for aggressive solution ingress. Evidence from broader 3D-printed cementitious systems supports this interpretation. Bradshaw et al. [28] noted that chemical resistance under sulfuric acid exposure remains a durability challenge for 3D-printed concrete, while supplementary cementitious materials such as silica fume can improve resistance by increasing microstructural compactness. In the studies reviewed by Bradshaw et al. [28], printed specimens exposed to 3% sulfuric acid showed mass loss values up to 49.1%, whereas a mixture containing 10% silica fume exhibited only 2.7% mass loss after 140 days, highlighting the importance of pore refinement and microstructural control in acid resistance.

Recent geopolymer-specific evidence indicates that nano-additives can improve acid resistance when their dosage is properly optimized. Gardezi et al. [41] evaluated MK–FA-based 3DPGPMs containing 0–0.75% nano-ZnO after 28 days of exposure to 5% HCl. The best post-acid performance was obtained with 0.5% nano-ZnO, where compressive and flexural strengths increased by 33.64% and 42.86%, respectively, compared with the acid-exposed reference mixture. This improvement was attributed to the filler effect of nano-ZnO, pore refinement, reduced void content, and densification of the geopolymer matrix [41]. SEM observations further showed a cracked and deteriorated matrix with unreacted FA particles in the reference specimen, whereas the 0.5% nano-ZnO specimen exhibited a denser matrix, N-A-S-H formation, and no visible internal deterioration after acid exposure [41].

Mass loss alone may not fully represent acid-induced deterioration in 3D-printed specimens. Inaty et al. [53] showed that the acid resistance of 3D-printed concrete should be interpreted together with residual mechanical performance, visual damage, porosity, and internal cracking. Their results indicated that printed specimens initially showed slightly better resistance than cast specimens under 0.5% sulfuric acid exposure, partly due to a denser matrix and lower water porosity; however, when acid penetrated the dense surface and reached elongated printing-induced pores, internal cracking and further deterioration developed [53]. Although this evidence is not geopolymer-specific, it suggests that acid attack in 3DPGPMs may also involve internal weakening and layer-interface-related degradation even when external mass loss appears limited.

Despite these promising findings, acid resistance remains insufficiently investigated for 3DPGPMs, particularly under long-term and field-relevant exposure conditions [13]. The potential acid stability of geopolymer matrices should not be directly generalized to printed systems because interlayer voids, weak interfaces, and direction-dependent transport pathways may reduce resistance under service conditions. Therefore, future studies should evaluate acid resistance under different acid types and concentrations, wetting–drying cycles, interlayer-oriented exposure, biogenic sulfuric acid attack, industrial acidic environments, and acid rain conditions. Combined indicators such as mass loss, residual strength, visual damage, pore structure, SEM-based deterioration, and interlayer integrity should be used to assess acid-induced damage more reliably.

### 5.6. Sulphate Attack

Geopolymer and alkali-activated materials may exhibit improved sulphate resistance compared with OPC-based concretes because their degradation mechanisms differ from those of Portland cement systems. In OPC-based materials, external sulphate attack is commonly associated with the reaction of sulphate ions with calcium hydroxide and calcium aluminate hydrates, leading to the formation of expansive products such as gypsum and ettringite [53]. In contrast, many low-calcium geopolymer systems based on FA or MK contain little or no portlandite and develop aluminosilicate-based gel structures, which may limit ettringite-related expansive cracking under sulphate exposure. Ricciotti et al. [52] noted that geopolymer materials can show superior sulphate corrosion resistance because their hardening process does not typically produce sulphate minerals such as ettringite.

However, this potential advantage should not be generalized to all geopolymer systems or directly transferred to 3DPGPMs without experimental verification. Sulphate resistance can be affected by calcium content, precursor chemistry, pore connectivity, curing regime, and transport pathways. In printed systems, porous interlayer regions, connected voids, shrinkage-induced microcracks, and weak layer interfaces may allow sulphate-bearing solutions to penetrate the structure more easily, even when the bulk geopolymer matrix is relatively resistant. Therefore, sulphate durability in 3DPGPMs should be interpreted as a combined function of binder chemistry, pore refinement, interlayer quality, printing direction, and exposure condition.

Specific studies on the sulphate resistance of 3DPGPMs remain limited. Mishra et al. [13] noted that long-term durability data for extrusion-based 3DPGPC are still insufficient, particularly for acid, sulphate, and salt resistance. The same review also emphasized that durability parameters such as moisture absorption, chemical attack, efflorescence, freeze–thaw resistance, and corrosion-related behavior have not yet been extensively investigated in the 3DPGPC domain [13]. This evidence indicates that clear sulphate-resistance trends cannot yet be established for printed geopolymer systems.

Future studies should directly evaluate sulphate exposure in 3DPGPMs by considering precursor chemistry, calcium content, activator system, curing regime, pore connectivity, interlayer quality, specimen orientation, and wetting–drying effects. Established procedures may provide a useful starting point, but testing protocols should be adapted to account for printing direction, interlayer regions, and direction-dependent transport. Performance should be assessed using combined indicators such as expansion, mass change, residual strength, visual deterioration, microstructural damage, and interlayer integrity. Such data are necessary to predict the long-term behavior of 3D-printed geopolymer elements exposed to sulphate-bearing groundwater, seawater, industrial wastewater, or cyclic wetting–drying environments.

### 5.7. Freeze–Thaw Resistance

Freeze–thaw resistance in 3DPGPMs should be evaluated by considering both matrix-level pore structure and printing-induced layer features. Evidence from 3D-printed concrete studies indicates that the absence of vibration and the sequential deposition of filaments can create weak interfacial zones, increased porosity, anisotropic pore networks, and direction-dependent cracking tendencies under freeze–thaw exposure [54]. These mechanisms are also relevant to 3DPGPMs because connected pores, interlayer voids, and weak layer interfaces may promote moisture ingress, ice formation, internal pressure development, and crack propagation during repeated freezing and thawing cycles.

Specimen orientation is particularly important because the tested direction may determine how many interlayer regions are intersected by moisture and damage pathways. Mousavi and Rangaraju [54] noted that vertically cored specimens may be more vulnerable than horizontally cored specimens because they intersect interlayer interfaces with higher porosity and weaker bonding. Direction-dependent freeze–thaw deterioration was also reported by Wang et al. [55] in 3D-printed desert sand concrete, where printed specimens were more vulnerable in the Y-direction than in the X-direction or cast counterparts after 300 freeze–thaw cycles. Although this evidence is not geopolymer-specific, it highlights the need to report printing direction, specimen extraction orientation, and exposure direction in freeze–thaw studies on 3DPGPMs.

Air-entraining admixtures may improve freeze–thaw resistance in 3D-printed cementitious systems by creating pressure-relief voids that reduce internal stress during freezing. Mousavi and Rangaraju [54] reviewed that air-entraining admixtures can reduce compressive strength loss after freeze–thaw exposure, with reported reductions of approximately 1.4–5.3% compared with control mixtures. They also noted that horizontally cored specimens containing 0.1% air-entraining admixture showed a 15% higher dynamic modulus of elasticity than control specimens and survived up to 300 freeze–thaw cycles without notable degradation [54]. However, the use of air entrainment in printable geopolymer mixtures should be optimized carefully because excessive air content may reduce strength, modify rheology, affect extrudability, and weaken interlayer bond quality.

Direct evidence on 3DPGPMs shows that nano-additives can improve freeze–thaw durability when they refine the pore structure without causing dispersion-related defects. Tanyildizi et al. [42] investigated MK–FA-based 3DPGPMs containing 0–0.75% nano-ZnO and subjected them to freeze–thaw cycles after 28 days of curing at 20 ± 2 °C. The mixture containing 0.5% nano-ZnO showed the highest freeze–thaw resistance, with a compressive strength retention rate of 95.72% after exposure [42]. This improvement was associated with the filler effect of nano-ZnO, reduced internal voids, matrix densification, and improved mechanical strength up to the optimum dosage; however, the lower strength retention at 0.75% nano-ZnO suggests that excessive dosage may cause dispersion problems or agglomeration-related weakening [42].

Broader 3D-printed cementitious literature also confirms the importance of pore refinement and optimized mix design. Bradshaw et al. [28] reviewed that porosity refinement is important for improving freeze–thaw durability, while Mousavi and Rangaraju [54] noted that nano-silica, silica fume, FA, and fibers may improve performance when properly proportioned by refining the pore structure, enhancing matrix densification, or improving crack resistance. However, these strategies should not be transferred directly to 3DPGPMs without validation because their effectiveness depends on liquid-to-binder ratio, printability, air-void stability, pore connectivity, interlayer bond quality, and specimen orientation.

Hybrid concrete–geopolymer systems provide additional but indirect evidence. Marczyk et al. [43] investigated 3D-printed and mold-cast hybrid specimens composed of 95% concrete and 5% FA- or MK-based geopolymer, with and without 0.5 wt% aramid roving. After 150 freeze–thaw cycles, the reduction in compressive strength reached up to 27% in molded specimens, whereas the decrease in 3D-printed specimens did not exceed 12% [43]. These findings suggest that layered production does not necessarily lead to inferior freeze–thaw resistance when mixture design, reinforcement strategy, and printing quality are controlled. However, the results should be interpreted cautiously because the initial compressive strength of the printed specimens was lower than that of the molded specimens, and one reinforced printed hybrid series was damaged during freeze–thaw testing [43].

Overall, satisfactory freeze–thaw resistance in 3DPGPMs may be achievable when connected porosity is limited, water uptake is reduced, pressure-relief voids are properly controlled, and sufficient interlayer integrity is maintained. Freeze–thaw durability should therefore be evaluated using combined indicators, including residual compressive and flexural strength, mass loss, relative dynamic modulus of elasticity, surface scaling, pore-structure evolution, water uptake, interlayer cracking, and layer-interface integrity. Future studies should assess these parameters under standardized cycling conditions while considering precursor chemistry, liquid-to-binder ratio, curing regime, air-void system, nano-additive dosage, printing direction, specimen orientation, interlayer time interval, and coupled exposure conditions such as freezing–thawing with wetting–drying or salt solution exposure.

### 5.8. High-Temperature Performance

**Figure 10:**
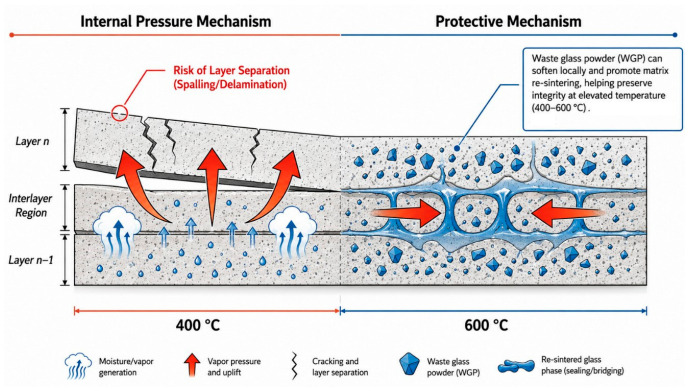
Internal pressure and WGP-assisted protective mechanisms in 3DPGPMs under elevated temperature. Conceptual synthesis based on Ramakrishnan et al. [56].

3DPGPMs may retain satisfactory residual strength at elevated temperatures depending on precursor chemistry, gel composition, pore structure, and moisture condition. Ramakrishnan et al. [56] showed that waste glass powder (WGP)-incorporated 3D-printable geopolymer mixtures exhibited lower strength loss after exposure to 400 °C and 600 °C, despite having lower initial compressive strength at ambient temperature. This improvement was attributed to the sintering-related melting and re-solidification of WGP within the matrix, which helped preserve structural integrity under heat exposure [56]. The competing internal pressure and WGP-assisted protective mechanisms are schematically illustrated in Figure 10. These findings suggest that precursor selection and thermally reactive filler phases may play an important role in improving the high-temperature stability of 3DPGPMs.

Spalling and thermal cracking at elevated temperature are commonly associated with the vaporization of physically bound and free water, which can generate internal pore pressure when vapor cannot escape from the matrix. In 3DPGPMs, this response may be further affected by the layered structure, because interlayer defects, anisotropic pore networks, and weak bonding between adjacent layers may promote localized cracking, delamination, or layer separation during heating. Therefore, high-temperature performance should be evaluated using combined indicators such as residual compressive and flexural strength, mass loss, visual damage, pore connectivity, cracking pattern, and interlayer integrity rather than relying only on residual strength.

### 5.9. Role of Layer Interface and Printing Direction in Durability

The layer-by-layer deposition process in 3DPGPMs creates interlayer regions that should be regarded not only as mechanical weak planes but also as durability-sensitive transport zones. Insufficient bonding between successive layers can increase local porosity, reduce mechanical continuity, and form preferential pathways for water, gases, CO_2_, chlorides, sulphates, and other aggressive agents [13]. This issue is strongly linked to open time and printing interval because surface stiffening, moisture loss, and partial setting of the previously deposited layer may reduce interfacial continuity and promote cold-joint-like regions. Evidence from geopolymer and cementitious 3D printing studies shows that interlayer bond quality and transport behavior are affected by activator-related structuration, printing interval, and interlayer porosity [34,47]. Therefore, durability assessment of 3DPGPMs should explicitly consider printing direction, specimen extraction orientation, exposure direction, transport direction, interlayer time interval, and layer-interface condition rather than relying only on matrix-level durability indicators.

From a practical perspective, the durability risks associated with layer interfaces can be reduced through coordinated optimization of mixture design, rheological evolution, curing regime, and printing parameters. Nozzle height, standoff distance, layer thickness, printing speed, extrusion flow rate, and interlayer interval should be controlled to ensure sufficient layer contact, uniform filament geometry, and limited interfacial void formation. When long open times are unavoidable, surface reactivation strategies such as controlled surface wetting, mechanical roughening, bonding agents, or compatible fresh geopolymer paste may be considered; however, their effectiveness should be directly validated for geopolymer-based printable systems. Table 5 provides a qualitative synthesis of how major durability parameters may differ among 3DPGPMs, cast geopolymers, and OPC-based 3D-printed cementitious systems.

The durability performance of 3DPGPMs can be interpreted through the correlation between microscale gel chemistry, pore structure, interlayer interface quality, and macroscopic transport behavior. A dense geopolymer matrix with refined and disconnected pores can reduce water uptake, permeability, chloride penetration, carbonation progress, and freeze–thaw damage. However, printing-induced interlayer voids, cold-joint-like regions, anisotropic pore networks, and weak interfaces may create preferential transport pathways even when the bulk matrix is relatively dense. Quantitative findings support this interpretation: 0.5% nano-ZnO improved the freeze–thaw resistance of 3DPGPMs, with a reported compressive strength retention rate of 95.72% after freeze–thaw exposure [42], whereas excessive nano-additive dosage may cause dispersion-related defects. In hybrid concrete–geopolymer systems, the compressive strength reduction after 150 freeze–thaw cycles was reported to be up to 27% in molded specimens, while the decrease in 3D-printed specimens did not exceed 12%, although these results should be interpreted cautiously because of differences in initial strength and material configuration [43]. These examples show that durability regulation rules should consider not only exposure type and matrix composition, but also pore connectivity, interlayer interval, specimen orientation, exposure direction, curing regime, and the integrity of layer interfaces.

### 5.10. Coupled and Multi-Exposure Deterioration Mechanisms

Real-world 3DPGPM structures are unlikely to experience only one isolated deterioration mechanism. Chloride penetration, carbonation, wetting–drying, acid or sulphate attack, freeze–thaw cycling, elevated temperature, and mechanical loading may occur simultaneously or sequentially and may accelerate one another. For example, freeze–thaw cycles can generate microcracks and increase pore connectivity, thereby facilitating subsequent water absorption, chloride ingress, carbonation, or acid attack. Similarly, acid or sulphate exposure may weaken the geopolymer matrix and interlayer regions, while elevated temperature can modify gel structure, increase microcracking, and reduce residual mechanical capacity. In printed systems, these coupled effects may be intensified by interlayer voids, weak layer contacts, anisotropic pore networks, and direction-dependent transport pathways. Therefore, future durability studies should move beyond single-exposure tests and evaluate combined exposure scenarios, exposure sequence, specimen orientation, interlayer quality, pore connectivity, residual strength, cracking pattern, and microstructural evolution under service-relevant conditions.

## 6. Sustainability of 3D-Printed Composites: LCA and Use of Local Resources

In this section, the sustainability performance of 3D-printed geopolymer composites is analyzed in comparison with conventional OPC-based systems and cast-in-place geopolymer mixtures, with a particular focus on LCA outcomes and the use of local and waste-derived materials.

### 6.1. CO_2_ Footprint and Cast Geopolymer

**Figure 11:**
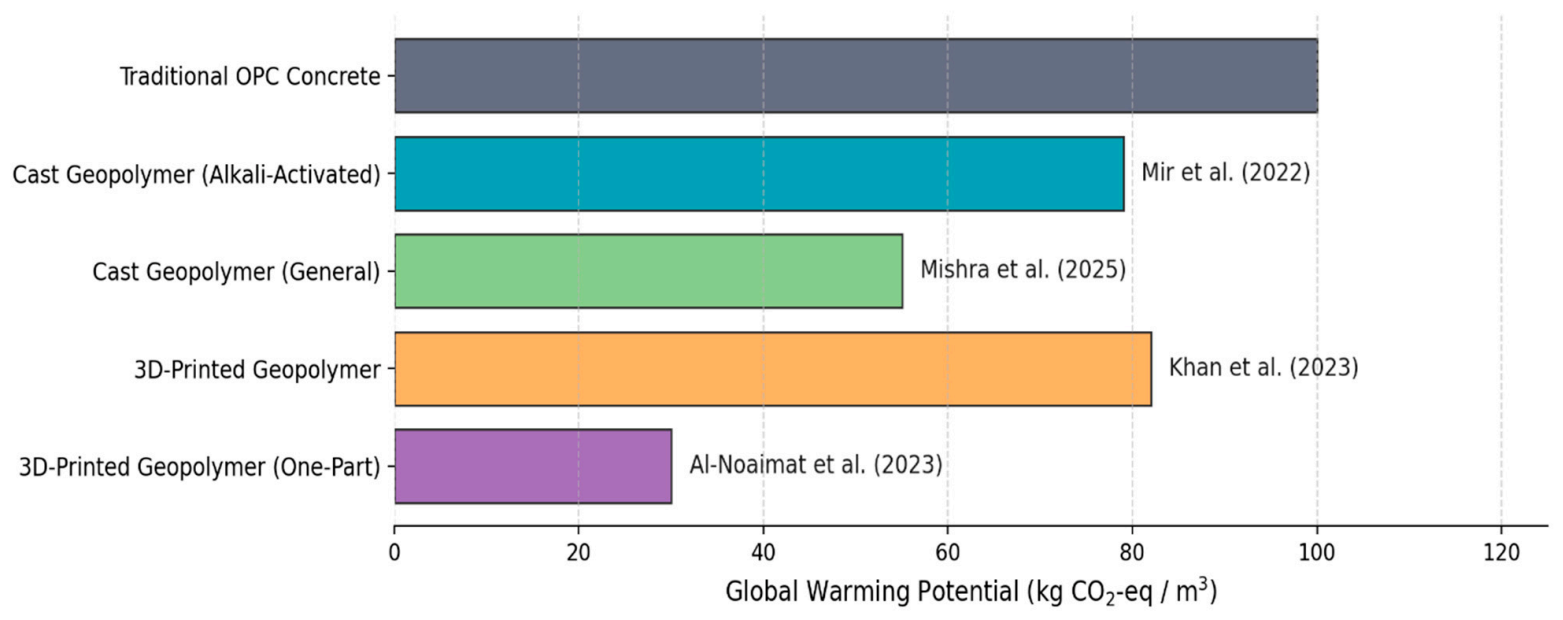
Comparison of GWP of OPC concrete and geopolymer concretes, adapted from Mir et al. [57], Mishra et al. [13], Khan et al. [58], and Al-Noaimat et al. [20].

Previous studies indicate that both cast geopolymer concretes and 3D-printed geopolymer systems can reduce global warming potential (GWP) compared with conventional OPC-based systems. However, this advantage should not be treated as automatic, because the environmental performance of geopolymer mixtures is highly sensitive to mixture design, precursor source, activator content, curing demand, transportation distance, electricity consumption, and LCA boundaries. Mishra et al. [13] reported that geopolymer concrete can exhibit approximately 40–50% lower GWP than conventional cementitious concrete, while Al-Noaimat et al. [20] showed that one-part geopolymers may provide lower total environmental impact and, at equivalent strength levels, substantial reductions in CO_2_ emissions and embodied energy compared with 3D-printed OPC mixtures. Figure 11 summarizes the comparative GWP trends reported for OPC concrete, cast geopolymer systems, and 3D-printed geopolymer systems.

For cast geopolymer binders, the use of local and waste-derived precursors can provide a meaningful CO_2_ advantage when clinker is fully replaced and transport-related impacts are controlled. Mir et al. [57] used construction and demolition waste (CDW) from the Eskişehir region, including perforated brick, red clay brick, roof tile, and glass, as 100% binder precursor and reported a GWP of 635 kg CO_2_-eq for 1 m^3^ of CDW-based binder, corresponding to approximately 21% lower GWP than an OPC 32.5 binder with the same strength level. Similarly, Dai et al. [59] calculated the embodied CO_2_ of alkali-activated slag–MK mixtures suitable for 3D printing and showed that all slag–MK mixtures produced lower emissions than the reference Portland cement mixture. In particular, the mixture containing 20% MK achieved approximately 30–32% lower embodied CO_2_ than Portland cement, indicating that appropriately designed alkali-activated binders can reduce CO_2_ emissions even before printing-related material efficiency is considered [59].

In 3D-printed geopolymer systems, the environmental outcome is even more dependent on process assumptions and formulation choices. Yao et al. [17] reported that an initial laboratory-scale 3D-printed geopolymer concrete scenario could perform worse than OPC panels in several impact categories, mainly because of high sodium silicate content and long-distance transport of raw materials. However, when upscaling, reduced electricity consumption, slag and FA allocation as waste, and lower Na_2_SiO_3_ content were considered, the GWP of the 3D-printed geopolymer system decreased to about 60% of that of the reference OPC concrete [17]. This shows that 3D-printed geopolymer systems can provide climate benefits, but only when activator demand, energy consumption, precursor allocation, and transport distance are carefully optimized.

The use of local CDW can further improve the sustainability of 3D-printed geopolymer systems. Khan et al. [58] compared a CDW–geopolymer 3D-printed wall, an OPC-based 3D-printed wall, and a conventional masonry wall using CDW and slag collected from demolition sites in the Ankara region. For 1 m^3^ of wall, the reported GWP values were approximately 488 kg CO_2_-eq for the CDW–geopolymer 3D-printed wall, 596 kg CO_2_-eq for the OPC-based 3D-printed wall, and 534 kg CO_2_-eq for the masonry wall [58]. These results indicate that locally sourced CDW-based geopolymer printing can reduce GWP by approximately 18% compared with OPC-based 3D printing while also performing better than the conventional masonry alternative.

Foam-geopolymer-based 3D printing also shows potential sustainability benefits when the functional unit reflects thermal performance and material efficiency. Balina et al. [60] conducted an LCA in Latvia using a FA–slag–sand foam geopolymer matrix incorporating local clay brick waste and autoclaved aerated concrete waste. For 1 m^3^ of wall, the GWP decreased from approximately 152.6 kg CO_2_-eq for the reference mixture to approximately 141 kg CO_2_-eq for the waste-incorporated scenario, corresponding to an additional reduction of about 7% [60]. When the functional unit was defined according to thermal performance, the reductions in GWP and several other impact categories increased to approximately 16–18%, suggesting that lightweight or insulation-oriented printed geopolymer systems should be evaluated using performance-based functional units rather than volume alone [60].

**Figure 12:**
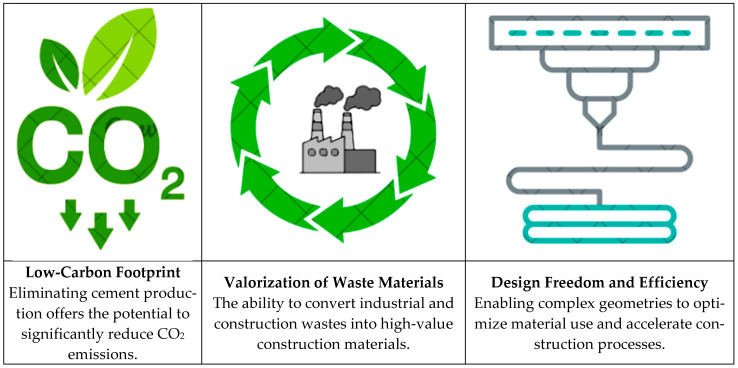
Three components of sustainability gains in 3D-printed geopolymers (CO_2_ reduction, circular economy, and material/process efficiency) (adapted from Khan et al. [58]; Balina et al. [60]; Yao et al. [17]). Figure prepared with Canva (https://www.canva.com/).

The sustainability gains of 3D-printed geopolymers can therefore be grouped into three interacting components: lower CO_2_ footprint through OPC replacement, circular economy benefits through the valorization of industrial and construction wastes, and material/process efficiency enabled by digital fabrication and geometry optimization. Figure 12 schematically summarizes these three components.

Overall, available studies suggest a conditional CO_2_ hierarchy in which OPC concrete generally shows the highest emissions, cast geopolymers can achieve medium to low emissions depending on precursor and activator choices, and optimized 3D-printed geopolymer systems may achieve the lower GWP when local waste-derived binders, reduced activator demand, efficient printing, and appropriate functional units are used. However, this hierarchy remains highly sensitive to alkali activator content, sodium silicate demand, electricity consumption, regional energy mix, transportation distance, allocation assumptions, and service-performance requirements. Table 6 provides a qualitative comparison of OPC concrete, cast geopolymer concrete, and 3D-printed geopolymer concrete from the perspective of CO_2_ emissions, activator impact, mixture design sensitivity, waste material use, and energy consumption.

### 6.2. Activator Impacts and One-Part Geopolymer Potential

**Figure 13:**
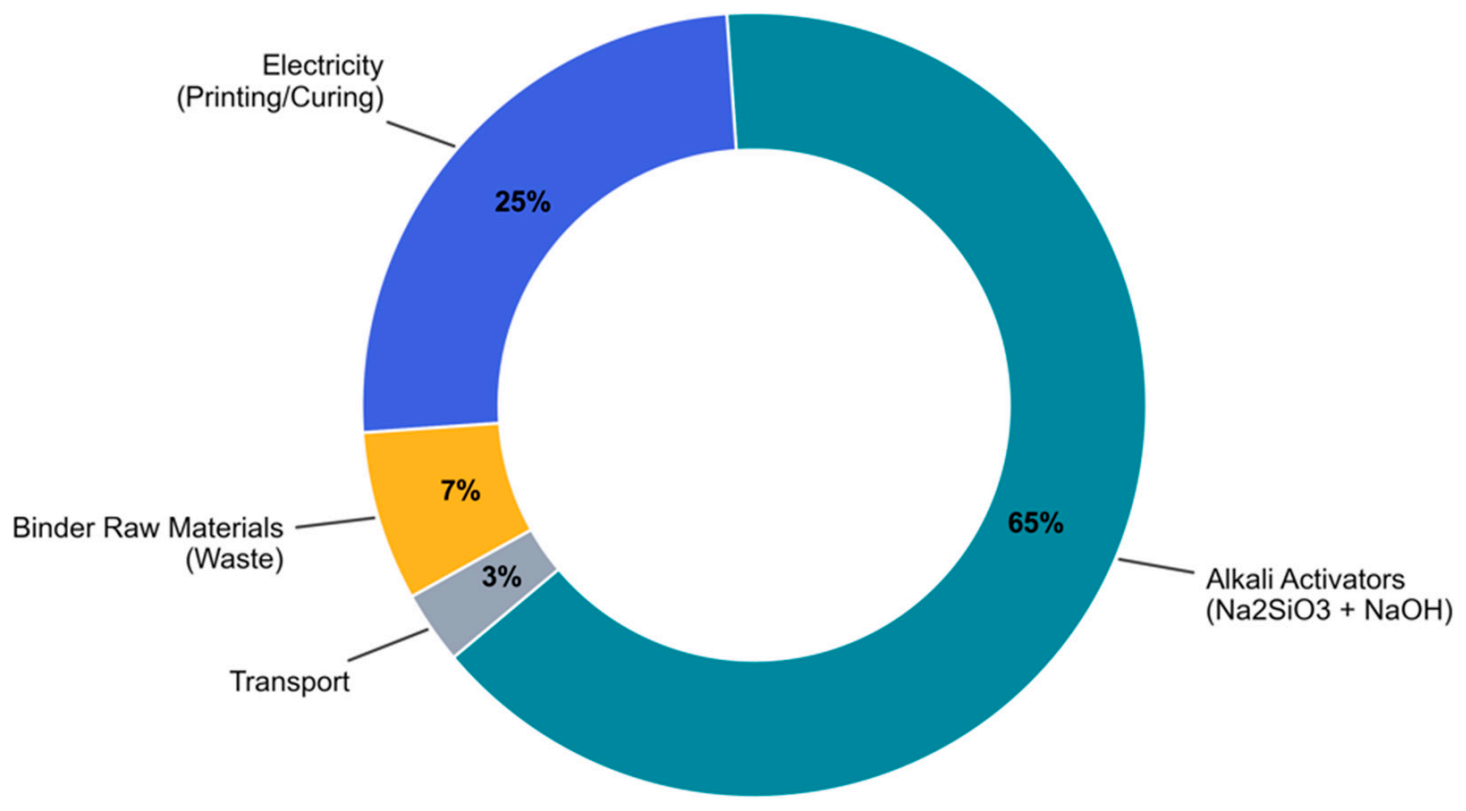
Component-wise distribution of environmental impacts in 3D-printed geopolymer systems, adapted from Yao et al. [17].

LCA studies consistently indicate that alkali activators are among the dominant environmental hotspots in geopolymer and alkali-activated systems. In 3D-printed geopolymer concrete, Yao et al. [17] showed that sodium silicate production was the dominant contributor to GWP and several other impact categories, and that mixtures with high silicate content could perform worse than OPC-based systems under laboratory-scale baseline assumptions. Similarly, Mir et al. [57] found that NaOH and, to a lesser extent, Ca(OH)_2_ production contributed significantly to ozone depletion and toxicity-related categories in CDW-based geopolymer binders. Khan et al. [58] also reported that, in CDW-based 3D-printed geopolymer walls, electricity use for grinding and printing was the largest GWP contributor, followed by NaOH and Ca(OH)_2_ production. These findings show that the environmental advantage gained by replacing OPC can be reduced or even offset when activator demand, activator type, and process energy are not carefully controlled. Figure 13 illustrates the component-wise distribution of environmental impacts in a representative 3D-printed geopolymer system.

Activator-related impacts are not limited to sodium silicate–sodium hydroxide systems. Roux et al. [61] conducted a multi-criteria LCA of a potassium silicate–MK-based 3DPGPM and reported that potassium silicate solution was the main source of environmental impacts, followed by MK and transportation, while electricity made a smaller contribution in that specific scenario. In contrast, Balina et al. [60] showed that electricity consumption, particularly during oven-drying, dominated the environmental burden of 3D-printed foam geopolymer systems, accounting for approximately 62–65% of the total impact, followed by hydrogen peroxide, sodium silicate, and sodium hydroxide. Therefore, the main environmental hotspot may shift depending on activator type, binder chemistry, foaming process, curing/drying regime, and regional energy assumptions.

**Figure 14:**
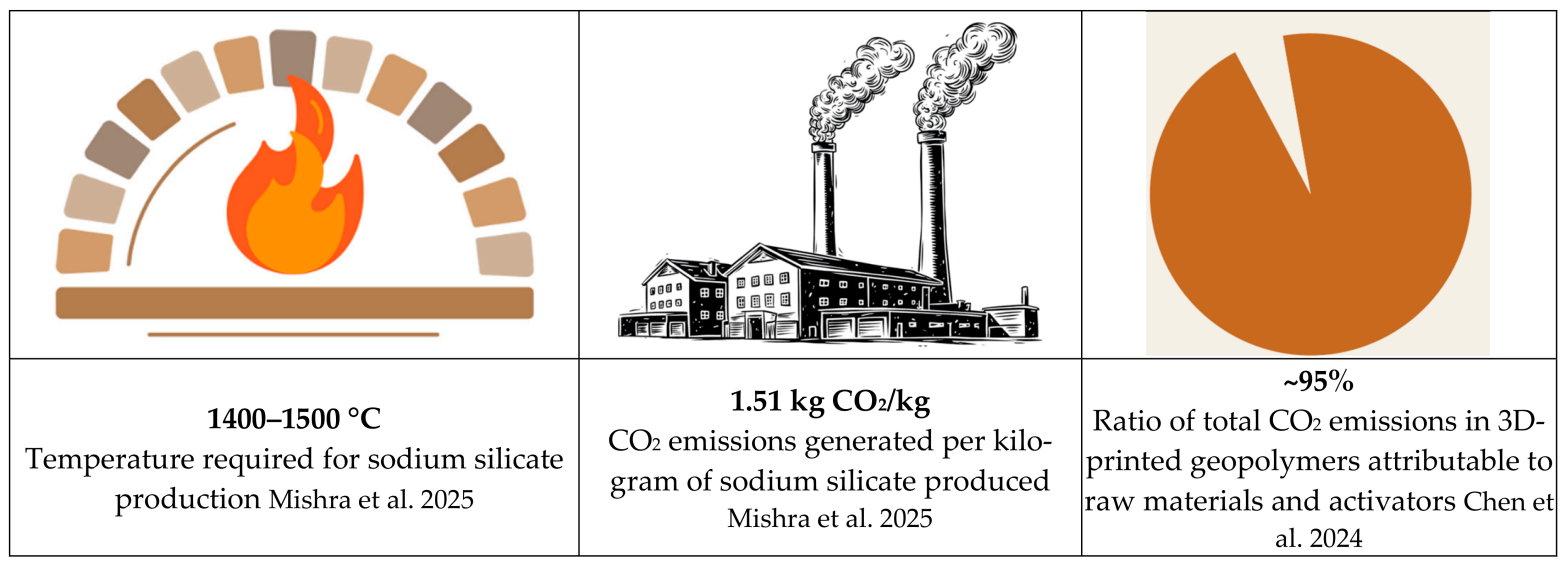
Energy-intensive production of alkali activators, particularly sodium silicate, and their contribution to the environmental burden in 3D-printed geopolymer systems, adapted from Mishra et al. [13] and Chen et al. [3].

The high environmental burden of sodium silicate is closely related to its energy-intensive production route. Chen et al. [3] reported that approximately 95% of total CO_2_ emissions and 98% of energy consumption in 3D-printed geopolymers were associated with raw materials and activators, indicating that sustainable mixture design should begin with activator selection. Sodium silicate production requires furnace temperatures of approximately 1400–1500 °C and generates about 1.51 kg CO_2_ per kilogram of product, while mining and processing further increase its environmental burden [13]. Therefore, sodium silicate should be treated as a critical sustainability hotspot rather than a neutral replacement component. Figure 14 separately summarizes the energy-intensive production of alkali activators, particularly sodium silicate, and their contribution to the environmental burden of 3D-printed geopolymer systems.

**Figure 15:**
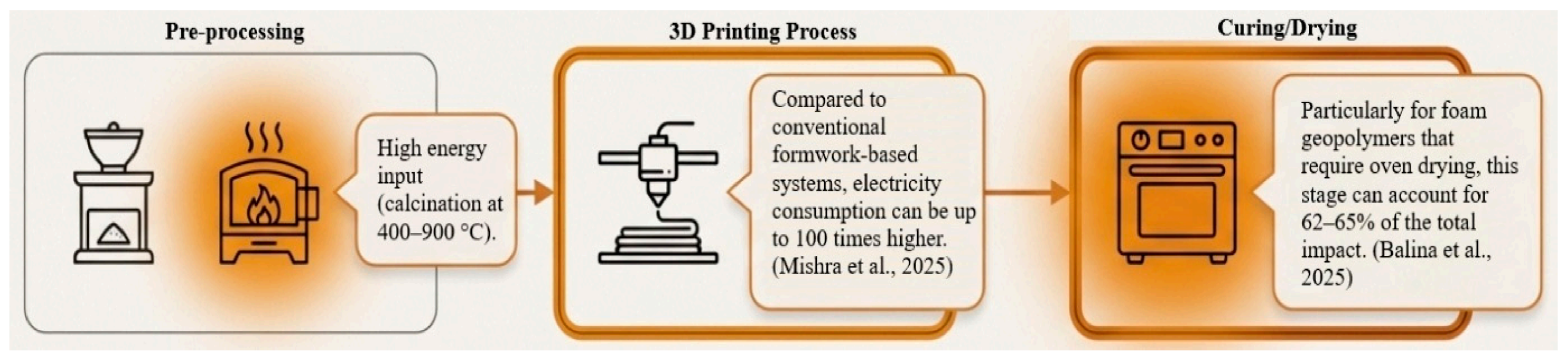
Energy consumption in the production of 3D-printed geopolymers, including pre-processing, 3D printing, and curing/drying stages, adapted from Mishra et al. [13] and Balina et al. [60].

Process energy can also contribute substantially to the environmental profile of 3D-printed geopolymer systems. Mishra et al. [13] noted that 3D printing may consume substantially more electricity than conventional formwork-based systems, while calcination of raw materials such as clay and laterite at 400–900 °C and curing at 40–85 °C can further increase total energy demand and associated CO_2_ emissions. In foam geopolymer systems, Balina et al. [60] showed that oven-drying may dominate the total environmental impact. These findings indicate that activator reduction alone is not sufficient; printing electricity, pre-processing, calcination, curing, and drying requirements should also be minimized. Figure 15 presents the main energy-consuming stages in the production of 3D-printed geopolymers.

One-part or “just-add-water” geopolymer systems have been proposed as a promising route to reduce the practical and environmental drawbacks of conventional two-part liquid activator systems. In two-part systems, highly alkaline and often viscous sodium hydroxide and sodium silicate solutions can create logistical, storage, transport, handling, and occupational safety challenges during mixing, pumping, and extrusion. Single-component systems can reduce direct handling of corrosive liquid activators and improve compatibility with dry-mix production and on-site construction workflows [20,62]. From an environmental perspective, Al-Noaimat et al. [20] reported that one-part geopolymer systems suitable for 3D printing can achieve up to 70% lower CO_2_ emissions and around 15% lower embodied energy than OPC at equivalent strength levels. However, because more than 80% of the total energy in one-part mixtures may still originate from the activator, one-part technology should be interpreted as a promising but not automatically low-impact solution.

Despite their potential, one-part printable geopolymer systems introduce several material and process challenges. The dissolution of solid activators may generate heat and accelerate reaction kinetics, increasing the risk of premature stiffening, unstable flow, nozzle clogging, and loss of open time if the formulation is not properly controlled. Therefore, one-part printable mixtures require careful optimization of dry activator type, precursor reactivity, setting behavior, moisture sensitivity, water demand, particle packing, thixotropic build-up, interlayer bond development, and early-age rigidity after deposition [15,20,62]. In this sense, one-part geopolymer design should be treated as a material–process co-design problem rather than a simple replacement of liquid activators with solid activators.

Low-alkali activation is another potential strategy for reducing activator-related environmental burden and improving practical feasibility. This approach may include Na_2_CO_3_, Ca(OH)_2_, low-dose silicate powders, or hybrid activator systems designed to reduce total alkali content, pH, handling risk, and activator impact. However, low-alkali systems may show different reaction kinetics, early-age strength development, curing requirements, and moisture sensitivity compared with conventional NaOH–sodium silicate-activated systems. More importantly, limited data are available on how low-alkali activation affects rheology, thixotropic build-up, open-time evolution, interlayer bonding, layer stability, and durability in 3D printing applications [20,52,62]. Therefore, low-alkali printable geopolymers should be evaluated not only by environmental impact and compressive strength but also by printability, interlayer bond quality, anisotropy, durability, and field-scale process stability.

Hybrid precursor combinations based on FA, GGBFS, and MK may help balance rheological control, early-age reaction, buildability, and hardened performance in one-part printable geopolymer mixtures. However, the optimum activator type, activator dosage, water-to-binder or liquid-to-binder ratio, and precursor proportion depend on precursor reactivity, calcium content, particle size distribution, and curing condition [1,15,20,63]. Accordingly, one-part geopolymer mixtures should be optimized through performance-based criteria rather than fixed compositional rules, with simultaneous consideration of safety, activator impact, printability, interlayer bond development, mechanical performance, durability, and long-term service requirements.

Overall, the sustainability of 3D-printed geopolymer systems is often limited by the combined burden of alkali activators and process energy. Shifting away from high-silicate two-part systems toward lower-impact activators, waste-derived components, solid/one-part formulations, and low-alkali activation may improve both environmental and practical performance. However, these strategies must be validated together with printing stability, open-time control, interlayer bonding, early-age buildability, durability, and field-scale implementation. From a cautious sustainability perspective, the most effective printable geopolymer system is not necessarily the one with the lowest cement content, but the one that achieves the required mechanical and durability performance with minimized activator demand, optimized process energy, local raw materials, safer handling, and reliable long-term performance.

### 6.3. Waste-Derived Geopolymers and Regional Supply Opportunities

One of the main sustainability advantages of geopolymer and alkali-activated systems is their ability to convert local waste and industrial by-product streams into value-added binders. This approach can reduce CO_2_ emissions, lower natural resource consumption, and support regional circular economy strategies, particularly in areas with abundant CDW, industrial by-products, or locally available aluminosilicate-rich materials. However, the environmental benefit of waste-derived geopolymers depends not only on replacing OPC but also on transportation distance, pre-treatment energy, grinding demand, activator content, local electricity mix, and the consistency of the waste stream.

Regional studies from Türkiye demonstrate the potential of local waste-based geopolymer binders. Mir et al. [57] produced geopolymers using CDW from the Eskişehir region as 100% binder precursor and reported that the CDW-based binder had approximately 21% lower GWP than an OPC 32.5 binder with comparable strength. However, the same study also showed that electricity consumption accounted for more than 60% of the total environmental impact, indicating that local waste utilization should be combined with low-carbon electricity and energy-efficient pre-treatment. Mir et al. [57] further suggested that increasing the renewable share in the Turkish electricity grid could reduce GWP to roughly one-third of its current value. These results show that the sustainability of waste-derived geopolymer binders is strongly controlled by both feedstock localization and process energy.

**Figure 16:**
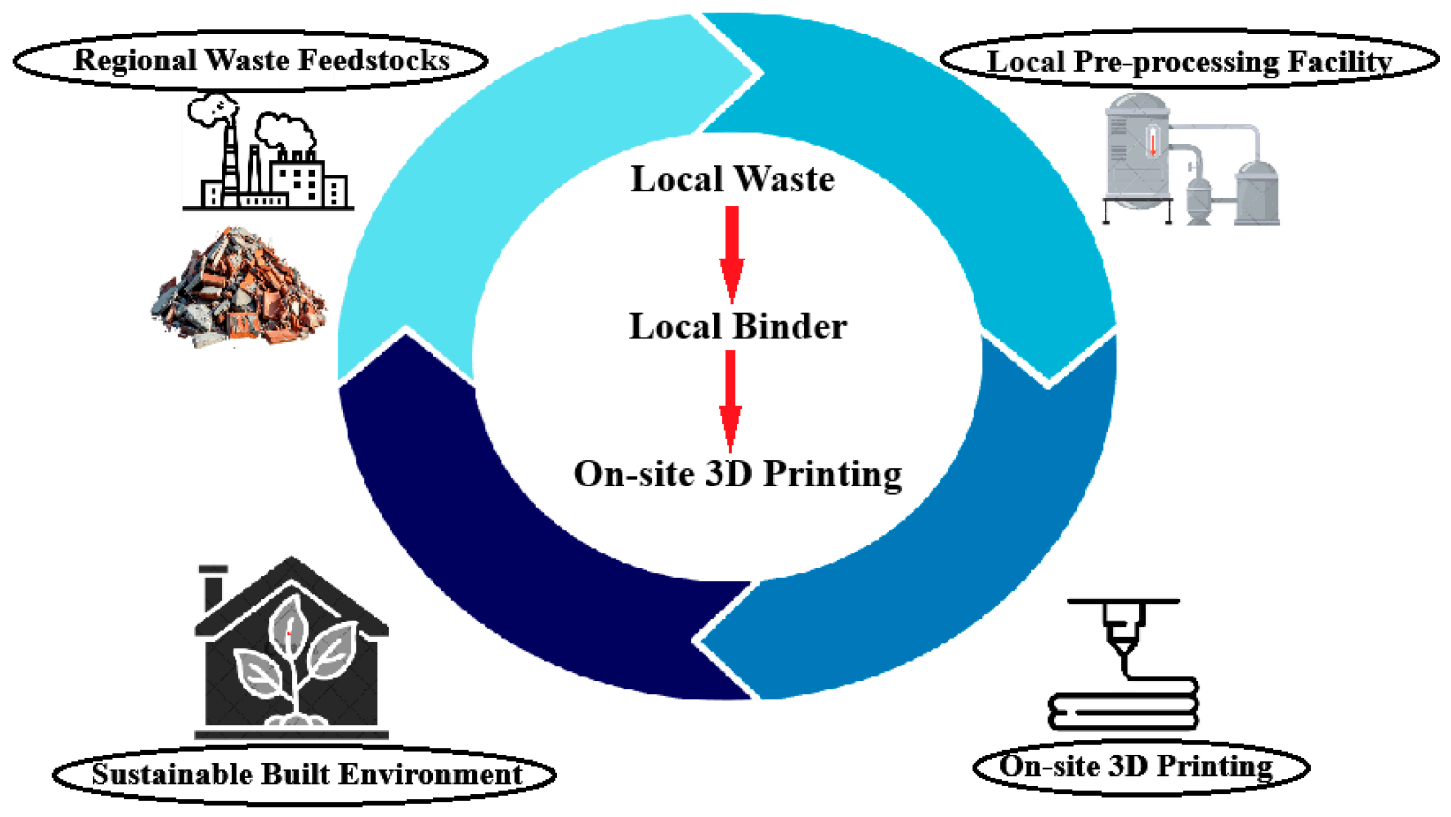
Circular-economy approach in 3D-printed geopolymers based on the “local waste → local binder → on-site 3D printing” model, adapted from selected references [57,58,64].

The regional potential becomes more evident when waste-derived binders are integrated with 3D printing. Khan et al. [58] developed 3D-printed wall systems using CDW streams and GGBFS collected from the Ankara region and reported that the CDW–geopolymer 3D-printed wall had lower GWP than both an OPC-based 3D-printed wall and a conventional masonry wall. Since the CDW was sourced from nearby demolition sites, transportation contributed only about 0.2% of the total GWP [58]. This finding supports the “local waste → local binder → on-site 3D printing” model as a promising pathway for reducing transport-related emissions and improving material efficiency. Figure 16 schematically illustrates this circular-economy approach.

**Figure 17:**
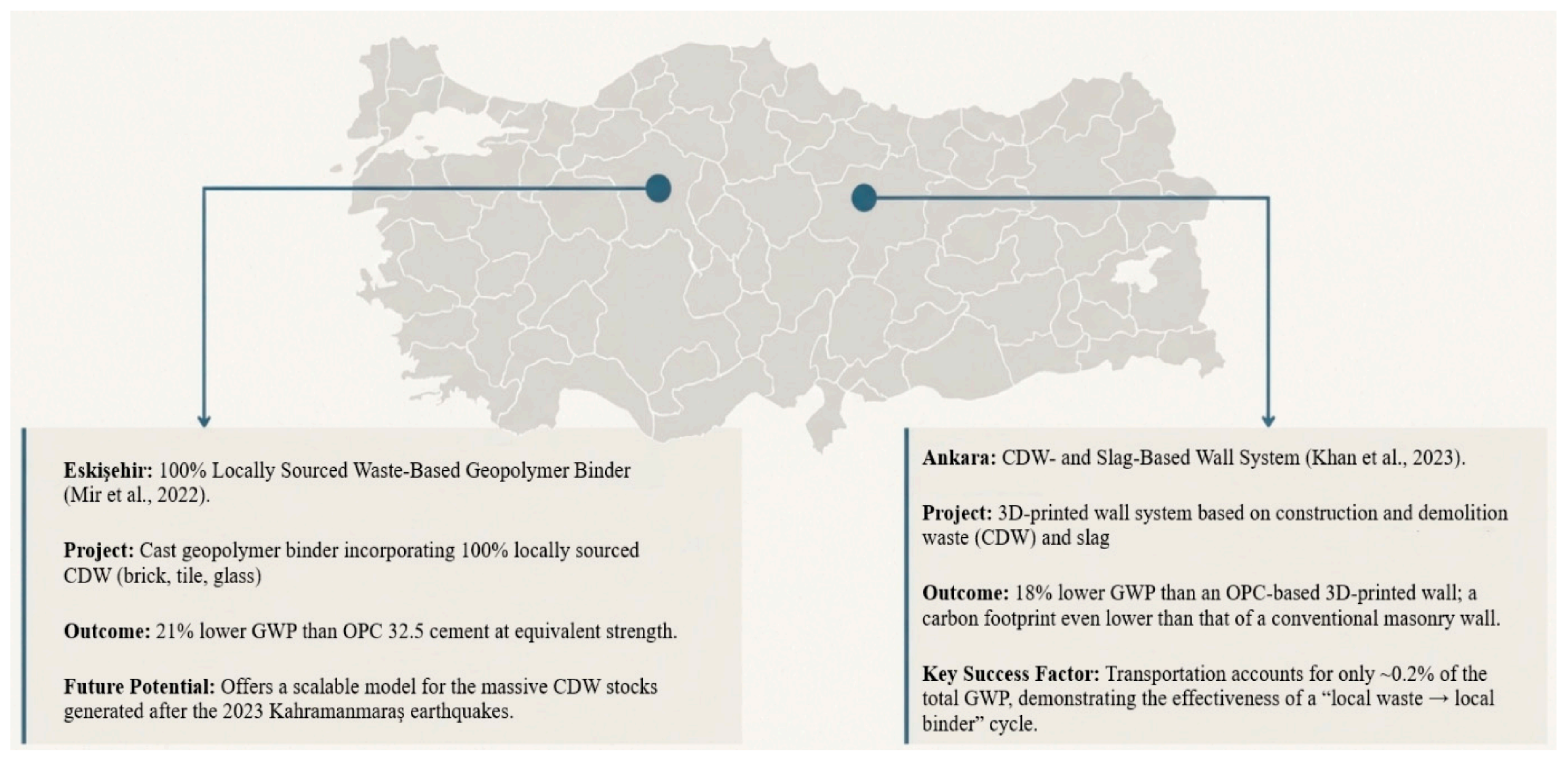
Local waste-based geopolymer examples from Türkiye and GWP reduction findings, adapted from Mir et al. [57] and Khan et al. [58].

The Turkish case is particularly important because large CDW stocks generated after the 2023 Kahramanmaraş earthquakes may create broader opportunities for regional waste-to-binder strategies, provided that material quality, contamination, sorting, grinding, and environmental safety are properly controlled. Figure 17 summarizes local waste-based geopolymer examples from Türkiye, including the Eskişehir CDW-based binder and the Ankara CDW–slag-based 3D-printed wall system.

Evidence from other regional contexts also confirms the importance of local feedstock selection. Balina et al. [60] used clay brick waste from a brick factory and autoclaved aerated concrete waste from demolition sites in 3D-printed foam geopolymer composites and reported lower environmental impacts than the reference mixture in GWP, land use, water consumption, and several other categories. This indicates that CDW-derived materials can be environmentally competitive for 3D-printed geopolymer insulation panels when the functional unit reflects thermal performance and material efficiency. Munir and Kärki [64] evaluated locally available industrial by-products, including green liquor sludge, waste fibers, FA, CDW, and flotation sand, as binder constituents for geopolymer 3D printing concrete. Their cost model showed that transportation costs were secondary, whereas waste pre-treatment processes such as crushing and grinding accounted for approximately 80–90% of total cost and energy consumption. Nevertheless, an on-site 3D-printed geopolymer concrete house was estimated to achieve about 30% lower total cost and roughly 50% lower energy demand than a conventional OPC concrete building [64]. These findings suggest that regional waste-based geopolymer printing can be feasible when local collection, sorting, and pre-treatment infrastructure are systematically planned.

Despite these opportunities, waste-derived geopolymer strategies should not rely only on conventional FA and GGBFS resources. FA availability may decrease as coal-fired power plants are phased out, while GGBFS is already widely consumed by the cement industry [13]. Therefore, a broader portfolio of local precursors should be considered, including CDW, red clay, zeolitic tuffs, agricultural ashes such as rice husk ash, silica-rich residues, and MK derived from local clays. Such diversification can improve supply security and support regional circular economy objectives, but each feedstock should be evaluated in terms of chemical composition, reactivity, variability, contamination risk, grinding demand, activator requirement, printability, and long-term durability.

**Figure 18:**
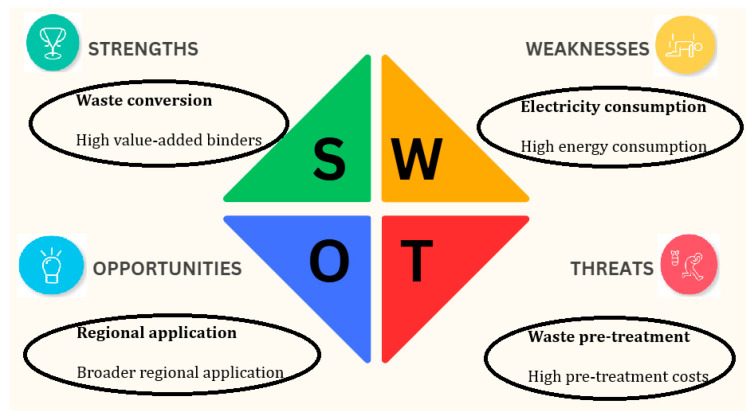
SWOT analysis of waste-derived geopolymer 3D printing systems, adapted from Mir et al. [57], Khan et al. [58], and Munir and Kärki [64].

The strengths, weaknesses, opportunities, and threats (SWOT) of waste-derived 3D-printed geopolymer systems can be summarized through a regional SWOT perspective. The main strengths are waste conversion and high-value binder production, while the main weaknesses are high electricity demand and pre-treatment energy. Regional application and local circular-economy development represent key opportunities, whereas feedstock variability, pre-treatment cost, and supply-chain organization remain important threats. Figure 18 provides a SWOT-based synthesis of these region-specific opportunities and limitations.

The sustainability of 3DPGPMs should be interpreted through operational and region-specific supply-chain conditions rather than through OPC replacement alone. Alkali activator production, particularly sodium silicate and NaOH, can dominate several environmental impact categories, while the benefits of FA, GGBFS, MK, CDW, or other waste-derived precursors depend on local availability, transport distance, pre-treatment energy, material consistency, and local electricity mix [17,57,58]. Therefore, a mixture that appears sustainable in one region may not provide the same benefit elsewhere if activators are imported, precursors are transported over long distances, or industrial by-product streams are chemically variable. Future LCA studies should therefore include regional material inventories, activator supply-chain scenarios, transportation distances, pre-treatment requirements, and service-life performance.

**Figure 19:**
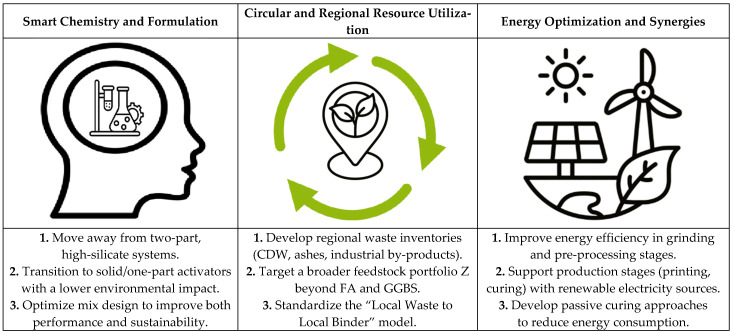
Strategic roadmap for sustainable 3D-printed geopolymers, including smart chemistry, circular regional resource utilization, and energy optimization, adapted from Mishra et al. [13], Al-Noaimat et al. [20], Khan et al. [58], Munir and Kärki [64], and Mir et al. [57].

Overall, 3D-printed geopolymer systems have strong sustainability potential when they are designed around local resource availability, low-impact activators, energy-efficient pre-treatment, and renewable electricity. A practical roadmap should include three parallel actions: smart chemistry and formulation, circular and regional resource utilization, and energy optimization. Smart formulation requires moving away from high-silicate two-part systems, increasing the use of solid or one-part activators with lower environmental impact, and optimizing mix design for both performance and sustainability. Circular resource utilization requires regional waste inventories, broader feedstock portfolios beyond FA and slag, and standardization of the “local waste to local binder” model. Energy optimization requires more efficient grinding and pre-processing, renewable electricity for printing and curing, and passive curing approaches to reduce energy demand. Figure 19 summarizes this strategic roadmap for sustainable 3D-printed geopolymers.

### 6.4. Economic Characteristics and Cost-Related Considerations

The economic feasibility of extrusion-based 3DPGPMs should be evaluated together with their environmental performance because low-carbon emissions alone do not guarantee practical adoption. The main potential economic advantages of 3D printing arise from formwork elimination, reduced labor demand, shorter construction time, digital material placement, and the possibility of producing complex geometries without additional mold-related cost. These advantages are particularly relevant for geometrically complex components, customized architectural elements, and applications where conventional formwork, labor, and construction time represent major cost burdens. However, the extent of cost reduction depends strongly on project scale, printer utilization rate, printing speed, mixture stability, failure rate during printing, curing requirements, and the degree of automation.

For 3DPGPMs, material-related costs may differ substantially from OPC-based 3D-printed systems. The use of FA, GGBFS, CDW-derived powders, MK, or other aluminosilicate precursors can reduce binder-related costs when these materials are locally available and require limited pre-treatment. However, grinding, drying, classification, chemical characterization, quality control, and transport can increase the total cost of waste-derived precursors. In addition, alkaline activators, particularly sodium silicate-based systems, may represent a significant cost component. Therefore, one-part or low-alkali geopolymer systems may improve economic feasibility when they reduce activator handling complexity, storage requirements, dosage-control problems, and on-site safety-related costs [20,64].

Economic performance is also sensitive to the production setting. Factory-based geopolymer 3D printing may benefit from controlled curing, stable material supply, automated quality control, and higher printer utilization, but it may involve additional transport and handling costs for prefabricated elements. On-site printing can reduce transport and assembly costs and may allow more direct use of local materials, but it requires robust control of environmental conditions, activator handling, pumping stability, and real-time process monitoring. Munir and Kärki [64] emphasized that the cost of geopolymer 3D printing depends on multiple factors, including material preparation, printing location, labor, equipment, energy, and production scale. Therefore, economic comparisons should not be based only on binder cost, but should include the full production chain from raw material processing to printing, curing, quality assurance, repair, and end-use performance.

The reviewed literature indicates that economic feasibility remains less mature than environmental assessment for 3DPGPMs. Future cost analyses should therefore combine life-cycle cost assessment with LCA to identify trade-offs between embodied carbon reduction and total production cost. Such analyses should include precursor availability, activator cost, pre-treatment energy, printer investment, maintenance, labor, printing failure rate, curing energy, quality-control requirements, transport distance, repair needs, and service-life assumptions. From a practical perspective, 3DPGPMs are likely to be economically competitive when local low-cost precursors are available, activator demand is minimized, printing is performed at sufficient scale, and the benefits of formwork elimination, labor reduction, and geometric flexibility outweigh equipment and quality-control costs [57,58,64].

## 7. Standards, Testing Protocols, and Practical Implementation

### 7.1. Existing 3DCP-Related Standards and Guidelines

Although extrusion-based additive manufacturing of cementitious materials has advanced rapidly, formalized standards for 3DCP remain largely pre-normative. Existing concrete standards and guidelines were primarily developed for conventionally cast materials and therefore do not fully capture the layer-by-layer, time-dependent, and interface-dominated nature of printed systems [5]. This limitation is particularly important because the performance of 3D-printed elements depends not only on hardened material properties but also on process parameters such as open time, extrusion rate, nozzle geometry, layer height, printing speed, environmental conditions, and interlayer bond formation. Therefore, standardization in 3DCP requires both material-based and process-based performance criteria.

RILEM TC 276-DFC emphasizes that 3D concrete printing should be understood as an integrated material–process–equipment system rather than a purely material-driven technology [5]. From this perspective, print quality and hardened performance are governed by the interaction between mixture rheology, structural build-up, nozzle configuration, extrusion flow rate, printing speed, layer height, interlayer interval, and environmental exposure during deposition. In geopolymer-based 3D printing, this interaction becomes even more critical because rheological evolution, setting behavior, and reaction kinetics are highly sensitive to precursor chemistry, activator composition, moisture condition, and process timing. Consequently, standards for 3DPGPMs should evaluate not only material properties such as strength, durability, and interlayer bond quality, but also the process and equipment conditions under which these properties are achieved.

### 7.2. Adaptation to Geopolymer Binders

Adapted testing and qualification procedures are required when printable geopolymer and alkali-activated binders are used as low-carbon alternatives for 3DCP. These binders differ from Portland cement-based systems in terms of reaction mechanism, rheological evolution, setting behavior, activator sensitivity, curing response, and microstructural development. Therefore, conventional cement-based test indicators alone may not adequately capture printability, buildability, interlayer bond development, durability-related transport behavior, or hardened performance. Archez et al. [25] emphasized that existing standards are not yet well adapted for additively manufactured geopolymer specimens and that comparisons between studies should be made cautiously because printing parameters, specimen dimensions, layer height, nozzle geometry, and testing orientation vary widely.

Fresh-state and early-age testing should be central to geopolymer-specific qualification because rheological build-up and open-time evolution directly control pumpability, extrudability, buildability, layer stability, and interlayer bond formation. RILEM TC 276-DFC noted that standardized test methods for structural build-up, thixotropy, and shape stability in 3D-printed cementitious materials are still under [5]. This issue becomes more critical for geopolymer and alkali-activated binders because precursor reactivity, activator chemistry, alkali concentration, molar ratios, additives, and mixing regime strongly influence yield stress, viscosity, thixotropy, structural build-up, printability, and buildability [8]. Therefore, OPC-based printability thresholds should not be directly transferred to geopolymer-based printable mixtures without binder-specific validation.

**Figure 20:**
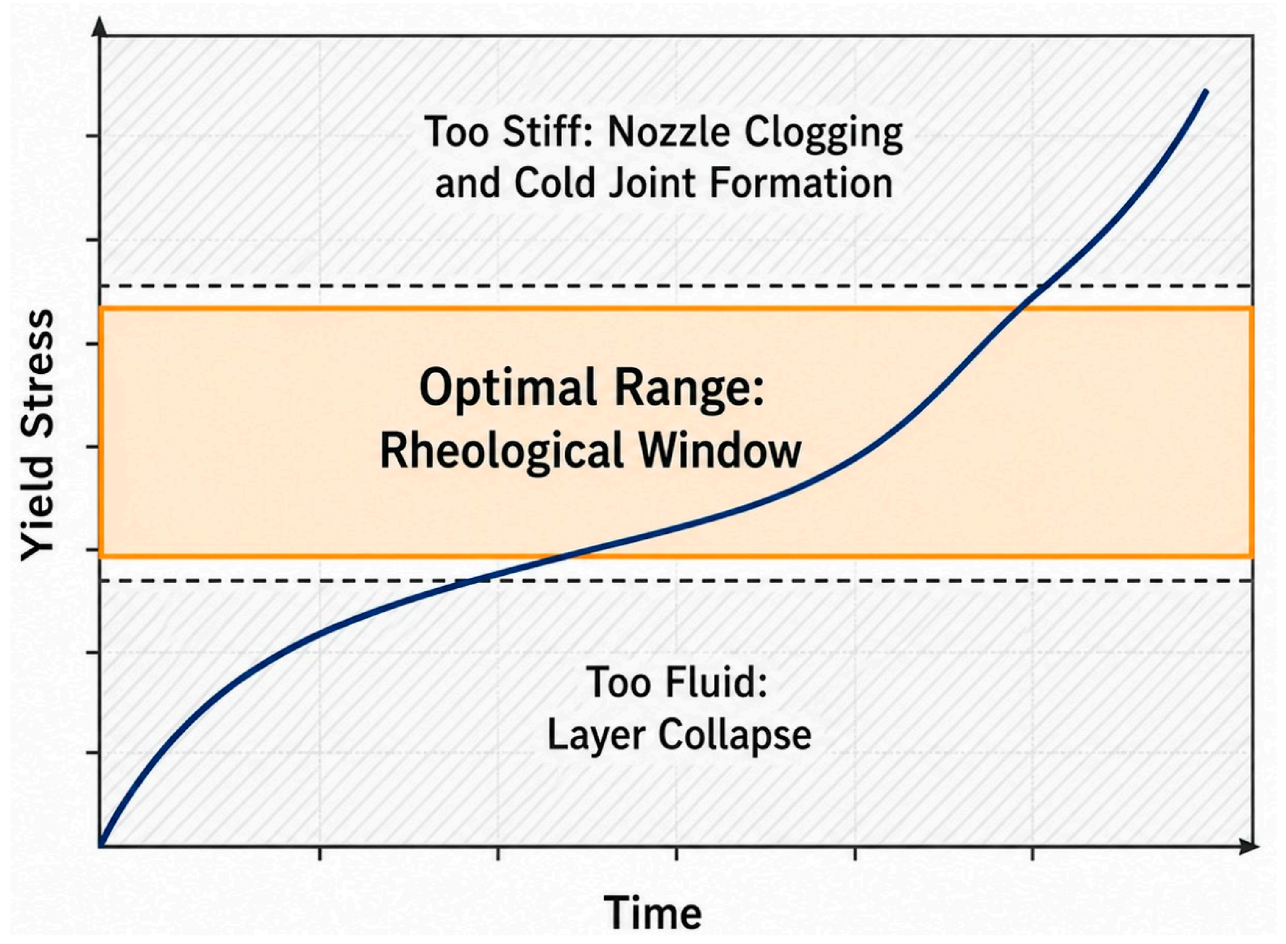
Rheological window required for printable alkali-activated and geopolymer mixtures. Conceptual synthesis based on Panda et al. [10].

The interaction between material behavior and printing equipment should also be tested and reported. Equipment platforms such as gantry systems and robotic arms require printable materials with controlled flow, stable extrusion, and rapid but not excessive structural build-up after deposition. Panda et al. [10] showed that alkali-activated slag mixtures require a narrow yield-stress window: the material must remain fluid enough to avoid extrusion blockage while also developing sufficient build-up to support subsequent layers. This narrow rheological window is schematically represented in Figure 20. Accordingly, residence time, shear history, extrusion flow rate, printing speed, layer interval, nozzle geometry, and environmental conditions should be evaluated together with rheological measurements because unstable flow, delayed deposition, partial stiffening, or poor layer contact may increase interlayer defects, reduce bond quality, and promote anisotropic mechanical and durability behavior.

Because geopolymer binders vary widely in precursor chemistry and activator composition, their qualification should focus on measurable performance indicators rather than fixed mix-design prescriptions. At the material and process level, the most relevant tests include rheological build-up, open-time evolution, shape retention, pumpability, extrudability, buildability, interlayer bond strength, direction-dependent mechanical response, curing sensitivity, pore connectivity, and durability-related transport behavior. In this context, qualification should evaluate not only whether a mixture can be printed, but also whether the printed element maintains sufficient interlayer integrity, mechanical reliability, and durability under realistic curing and service conditions. Safety-related issues associated with alkaline activators should also be considered during practical implementation, as discussed in the following section.

### 7.3. Pilot Applications and Industrial Challenges

Although considerable research has been conducted on geopolymer-based materials for 3D concrete printing, their practical implementation remains limited compared with OPC-based or blended cementitious systems. This limitation is partly associated with the greater familiarity of OPC-based binders in existing regulations, their more established testing and acceptance procedures, and their relatively predictable behavior under site-specific environmental conditions such as temperature, humidity, wind exposure, and curing availability. Therefore, the transition from laboratory-scale printable geopolymer mixtures to field-scale applications requires not only material optimization but also robust quality control, standardized testing protocols, activator safety management, and validation under realistic construction conditions.

Raw material variability remains one of the main challenges for the practical implementation of geopolymer-based 3DCP systems. Aluminosilicate precursors such as FA, slag, MK, and other industrial or construction residues may differ in chemical composition, fineness, particle size distribution, amorphous content, and reactivity, which can influence setting behavior, rheological evolution, early-age buildability, and hardened performance. Archez et al. [25] showed that differences in aluminosilicate source reactivity can affect setting time and reaction kinetics, while formulation and printing parameters strongly influence layer adhesion and mechanical performance. Therefore, reproducible geopolymer-based printing requires precursor characterization, mix-design adjustment protocols, process control, and performance-based qualification criteria rather than fixed binder recipes.

Occupational health and safety issues associated with alkaline activators represent another important barrier to industrial implementation. Liquid activators, particularly sodium hydroxide and sodium silicate solutions, require careful handling, storage, transport, and dosage control because of their high alkalinity and, in some cases, corrosive nature. Provis [65] emphasized that appropriate personal protective equipment is required when alkali-activated binders are used on site or in precast facilities, especially when caustic alkaline reagents are handled. These requirements may create additional practical challenges for on-site geopolymer-based printing, where continuous mixing, pumping, extrusion, and activator dosing must be controlled under variable construction-site conditions. Therefore, safer activator systems, automated dosing units, closed delivery systems, and dry or one-part formulations are important for improving the practical feasibility of geopolymer-based 3D printing.

One-part geopolymer systems may reduce some of the handling and safety barriers associated with two-component liquid activator systems, but their field-scale use still requires careful validation. Barve et al. [8] showed that the performance of 3D-printed geopolymer concrete is highly sensitive to precursor type, activator composition, molar ratios, additives, and mixing regime, all of which influence rheology, printability, buildability, and hardened behavior. Therefore, one-part systems should be assessed not only for safer handling but also for dry-mix quality control, moisture sensitivity, open-time stability, pumping and extrusion behavior, interlayer bond development, curing response, and long-term durability under realistic printing conditions.

Mineral-only geopolymer mix designs may also support practical implementation by reducing reliance on organic additives and improving regulatory compatibility. Archez et al. [25] demonstrated that geopolymer composites could be shaped by 3D printing using inorganic components such as wollastonite, glass fibers, and non-reactive aluminosilicate fillers, while printing speed and formulation parameters were optimized to improve slump control, layer adhesion, and flexural performance. This approach may be relevant from an implementation perspective because Archez et al. [25] linked the reduction in organic additives to the substitution of hazardous chemicals and the limitation of volatile organic compounds. However, mineral-only formulations should still be evaluated for long-term durability, printability robustness, interlayer bond quality, and compatibility with industrial-scale equipment.

From an industrial perspective, clear certification pathways, economic viability, and predictable performance are essential for the adoption of geopolymer-based 3DCP. Although geopolymer binders can reduce the embodied carbon associated with the binder component, environmental benefit alone is unlikely to ensure widespread adoption unless it is supported by reproducible material properties, standardized testing, reliable quality control, and demonstrated performance under practical construction conditions. Future pilot applications should therefore include field-scale printing trials, process monitoring, activator safety assessment, curing-condition control, durability validation, cost analysis, and documentation procedures that link mixture design, printing parameters, and final structural performance.

### 7.4. Need for Performance-Based Acceptance Criteria

Performance-based acceptance criteria are essential for the reliable implementation of 3D-printed geopolymer mortars (3DPGPMs). Conventional compressive strength tests on cubes or cylinders cannot adequately represent the process-dependent behavior of layer-by-layer fabrication, because the performance of printed elements is also governed by interlayer bond quality, anisotropy, pore connectivity, open-time evolution, and printing-induced defects. Although RILEM TC 276-DFC provides an important pre-normative framework for 3D concrete printing, its application to geopolymer and alkali-activated binders requires explicit consideration of their alkali-activation chemistry, fresh-state kinetics, curing sensitivity, and interface-controlled behavior [5]. Therefore, acceptance criteria for geopolymer-based 3DCP should be defined not only by strength, but also by whether the printed material and element achieve reliable interlayer integrity, process stability, and durability-related performance.

Current testing approaches remain insufficient for 3DPGPMs because most conventional concrete tests were developed for homogeneous cast specimens rather than layered, time-dependent, and interface-controlled materials. Cube or cylinder compressive strength tests may represent matrix strength, but they do not directly capture interlayer bond integrity, anisotropy, filament continuity, pore connectivity, or printing-induced weak planes. Similarly, durability tests for chloride penetration, carbonation, freeze–thaw resistance, acid attack, or elevated-temperature performance may be incomplete if specimen extraction direction, layer orientation, exposure surface, interlayer interval, curing regime, and printing parameters are not explicitly controlled. Future standards should therefore require orientation-dependent mechanical testing, direct interlayer tensile/shear/fracture tests, clearly defined anisotropy indices, pore-defect characterization, durability testing parallel and perpendicular to layer interfaces, and mandatory reporting of printing speed, extrusion rate, nozzle geometry, layer height, standoff distance, interlayer time interval, temperature, humidity, and curing regime.

**Figure 21:**
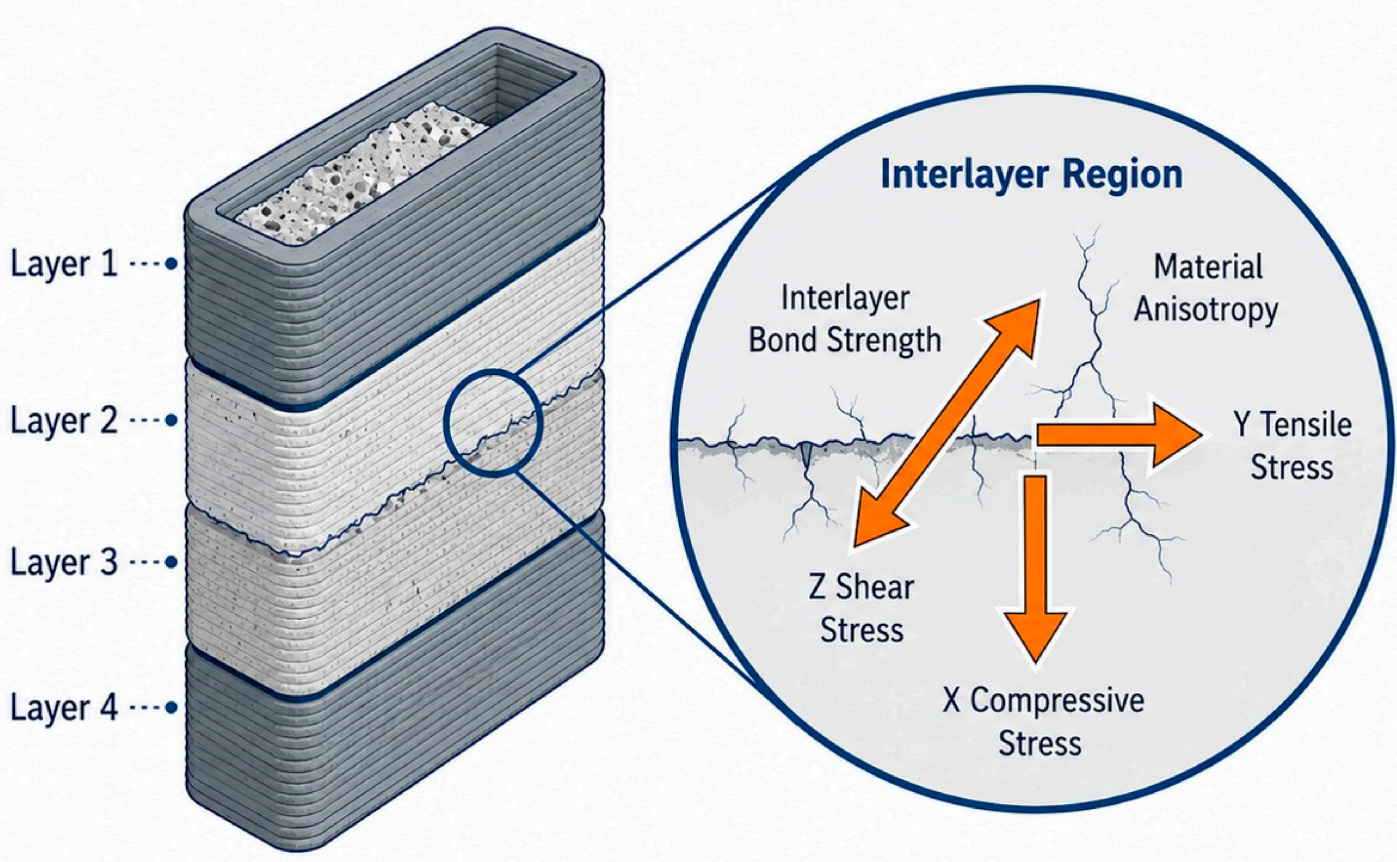
Effect of interlayer interval on bond quality and anisotropic mechanical vulnerability. Conceptual synthesis based on Archez et al. [25] and Panda et al. [32].

Interlayer bond quality and mechanical anisotropy should be among the primary acceptance parameters. Archez et al. [25] showed that short interlayer intervals can produce nearly imperceptible interfaces, whereas longer intervals lead to more visible interfacial zones and reduced layer interaction. Likewise, Panda et al. [32] reported that tensile bond strength in 3DPGPMs is strongly influenced by printing time gap, nozzle standoff distance, and, to a lesser extent, nozzle speed. These observations indicate that interlayer bond strength should not be treated as an isolated material property; rather, it should be accepted in relation to the printing conditions under which it is achieved. The effect of interlayer interval on interface quality and bond-related vulnerability is schematically summarized in Figure 21.

Acceptance criteria should also include process-reliability indicators that are directly linked to the quality of the final printed element. Panda et al. [10] showed that alkali-activated slag mixtures must satisfy a narrow rheological window that balances extrudability with sufficient structural build-up after deposition. Barve et al. [8] similarly demonstrated that the performance of 3D-printed geopolymer concrete depends on material composition, activator chemistry, mixing regime, rheological evolution, and printing parameters. For this reason, qualification should rely on minimum or target performance levels for parameters such as extrudability, buildability, dimensional stability, open-time evolution, interlayer cohesion, direction-dependent mechanical behavior, pore connectivity, and durability-related transport behavior, rather than on fixed mix-design limits alone.

To ensure comparability across studies and transferability to practical implementation, acceptance criteria should be accompanied by standardized reporting requirements. At a minimum, studies should report precursor type, activator composition, mixing regime, residence time, extrusion flow rate, nozzle geometry, layer height, printing speed, interlayer time interval, curing condition, specimen extraction orientation, loading direction, exposure direction, and dominant failure mode. Such reporting is particularly important for 3DPGPMs because surface stiffening, moisture loss, and setting progress directly affect interface quality and long-term transport performance. Table 7 summarizes the proposed performance-based acceptance criteria for 3DPGPMs based on the RILEM TC 276-DFC framework and selected studies on geopolymer-based 3D printing.

From an implementation perspective, performance-based acceptance criteria should support certification and regulatory approval without imposing overly rigid mix-design prescriptions. This is important because geopolymer mixtures may be produced using different precursor chemistries, activator systems, aggregate gradations, additives, and curing regimes. The current dominance of OPC-based 3DCP in commercial applications may partly reflect the absence of geopolymer-specific acceptance criteria, standardized qualification procedures, and predictable field-scale performance data. Therefore, successful industrial adoption of geopolymer-based 3D concrete printing requires the integration of RILEM’s pre-normative digital-fabrication framework with alkali-activated material science, process control, quality assurance, durability validation, and certification pathways. Without standardized and comparable testing protocols adapted to geopolymer-based 3DCP, regulatory acceptance and wider industrial implementation are likely to remain limited.

## 8. Research Gaps and Future Directions

Several research priorities emerge for one-part and low-alkali 3D-printed geopolymer systems. First, rheological design principles should be defined more clearly by comparing one-part systems with conventional two-part liquid-activated systems in terms of open time, structural build-up, extrusion stability, and interlayer bond development [20]. This comparison is necessary because solid activator dissolution, heat release, thixotropic build-up, and early-age reaction kinetics may jointly control printability, layer stability, and bond formation in printable geopolymer mixtures [20]. Second, the effects of low-alkali activation on long-term durability should be investigated through interface-focused experiments, particularly for chloride penetration, carbonation, and sulphate resistance, because reduced alkalinity and modified gel chemistry may alter both matrix resistance and interlayer transport behavior. Third, practical quality-control issues associated with powder activators, including moisture absorption, caking, dosage sensitivity, storage stability, and mixing uniformity, should be addressed before field-scale implementation. Finally, LCA and cost analysis should be directly linked to realistic 3D printing scenarios at both mixture and process levels, including activator type, precursor source, curing condition, energy demand, material waste, printing productivity, and structural optimization [17,18,19,20,21]. These priorities indicate that future research should evaluate one-part and low-alkali printable geopolymer systems through integrated material–process–durability–sustainability criteria rather than through compressive strength or printability alone.

### 8.1. Material–Process–Performance Relationships

Future research should move beyond isolated printability, strength, or durability tests and establish integrated material–process–microstructure–performance relationships for 3DPGPMs. The performance of these systems is governed by the coupled interaction between fresh-state rheology, printing parameters, microstructural development, mechanical response, and long-term durability. Existing reviews commonly discuss rheology through pumpability, extrudability, buildability, structural build-up, and open time, while microstructure is generally examined through imaging, phase analysis, or porosity measurements [1,6,15]. However, durability is often evaluated separately through independent transport or exposure tests, and studies that connect rheology, microstructure, mechanical performance, and durability within the same mixture family, curing regime, and experimental timeline remain limited [15,52]. This lack of integrated datasets restricts the development of predictive material–process–microstructure–durability models for 3D-printed geopolymer systems.

**Figure 22:**
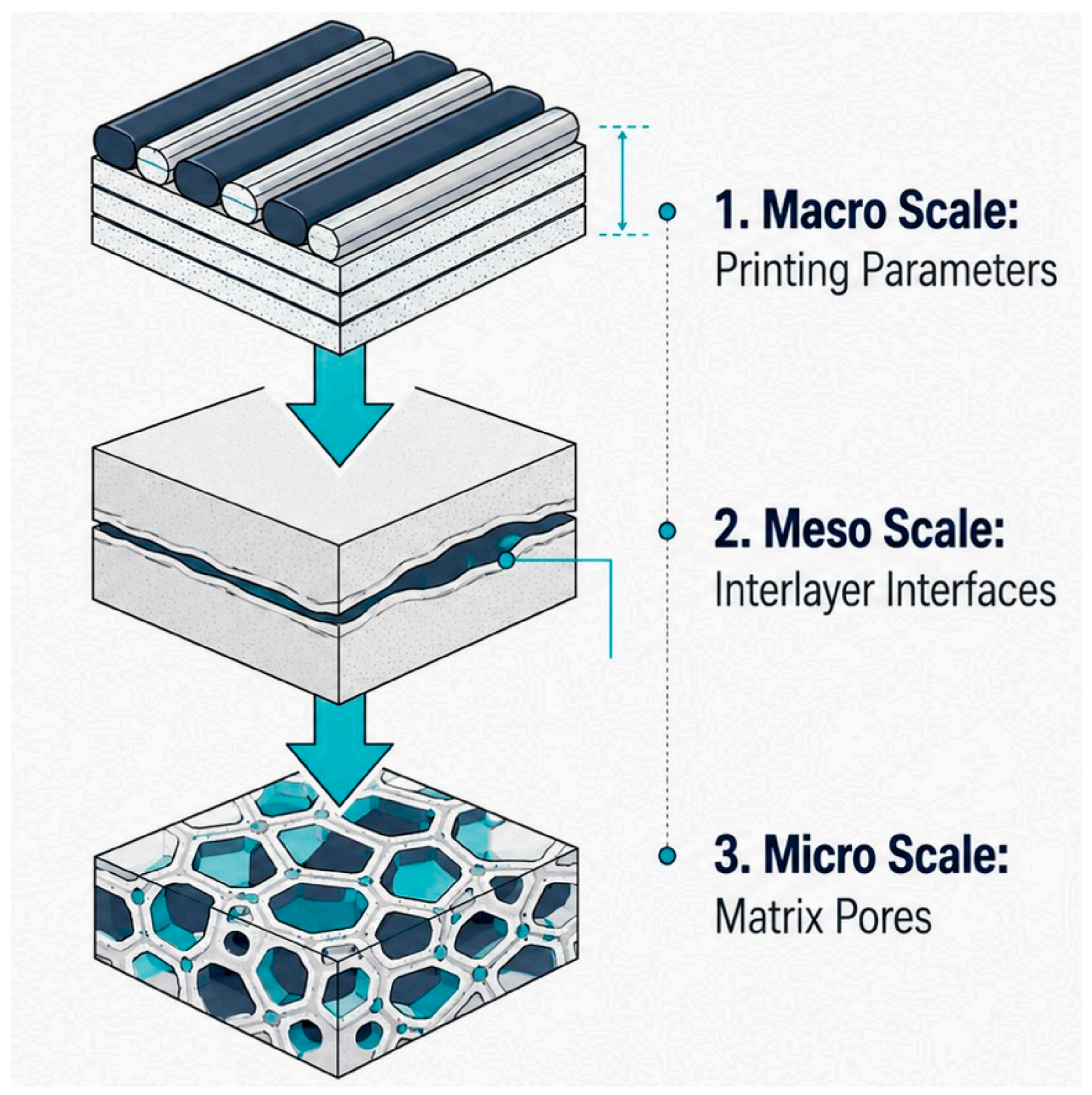
Multi-scale porosity and degradation pathways in 3DPGPMs. Conceptual synthesis based on Zhang et al. [66], Zhong and Zhang [15], Barve et al. [1], and Jaji et al. [16].

A key future need is the simultaneous characterization of macro-scale printing-induced voids and micro-/meso-scale pore structures. Macro-scale features such as filament geometry, path spacing, layer height, nozzle offset, interlayer voids, and printing direction may create preferential transport pathways, whereas gel pores, capillary pores, and pore connectivity control matrix-level permeability and durability. Cement-based 3D printing studies have shown that printing-induced void orientation and connectivity can strongly affect anisotropic mechanical and durability performance [66]. Similar relationships are beginning to be clarified for 3D-printed geopolymer systems; for example, Jaji et al. [16] linked pore structure, specimen orientation, permeability-related behavior, strength development, and microstructural evolution in slag-modified MK-based 3D-printed geopolymer concrete. Nevertheless, datasets that simultaneously connect printing parameters, multi-scale porosity, interlayer quality, mechanical performance, and durability indicators remain insufficient. The role of macro-, meso-, and micro-scale porosity in forming degradation pathways is schematically illustrated in Figure 22.

Future optimization should treat printability and long-term service performance as a single multi-objective problem rather than as separate design stages. Rheological parameters such as static and dynamic yield stress, plastic viscosity, thixotropy, and structural build-up rate control printability, layer stability, and interlayer contact. Panda et al. [32] showed that the tensile bond strength of 3DPGPMs is strongly affected by printing time gap, nozzle speed, and nozzle standoff distance, indicating that interlayer bond development is governed by both material state and process parameters. The time-dependent increase in static yield stress creates a fundamental trade-off: it improves shape retention and buildability after deposition, but it may reduce bonding between successive layers and increase cold-joint-like defects when the interlayer interval becomes too long. Therefore, fresh-state requirements such as pumpability, extrudability, buildability, shape stability, and open time should be optimized together with hardened-state indicators such as interlayer bond strength, direction-dependent mechanical behavior, pore connectivity, permeability, chloride penetration, carbonation resistance, freeze–thaw resistance, and residual mechanical performance under exposure conditions [1,2,15,67].

To support this optimization, future studies should generate time-resolved datasets covering the evolution of printable geopolymer mixtures from minutes to hours and days after mixing. Such datasets should link rheometer and ram-extruder measurements with actual printing behavior, monitor early-age heat flow and setting evolution, characterize porosity development in both bulk and interlayer regions, and combine imaging or pore-structure techniques such as X-ray CT, MIP, and N_2_ adsorption with mechanical and durability testing [1,16]. Recent work on slag-modified MK-based 3D-printed geopolymer concrete confirms that mixture design can simultaneously influence open time, interlayer bond strength, anisotropic mechanical performance, gel formation, and pore refinement [16]. From a cautious interpretation, future predictive models should therefore integrate time-dependent rheology, interlayer bond development, multi-scale pore structure, direction-dependent mechanical response, and durability under standardized exposure conditions, rather than relying only on compressive strength or printability indices.

### 8.2. Long-Term Durability and Field Exposure

Long-term durability remains one of the most critical research gaps for 3DPGPMs. In printed cementitious systems, durability performance can be strongly influenced by printing-induced void orientation, connected interlayer pores, and specimen direction, particularly for chloride penetration, carbonation, and freeze–thaw damage [66]. Interlayer bond quality is also a durability-sensitive feature because weak interfaces, cold-joint formation, layer time gaps, and interlayer voids can affect both mechanical continuity and transport pathways [2,68]. In 3D-printed geopolymer systems, these effects become more complex because precursor chemistry, activator type, curing condition, rheological evolution, printing configuration, and microstructural development jointly control durability [1,15]. Therefore, future studies should evaluate durability as a coupled material–process–interface problem rather than assuming that the intrinsic chemical resistance of cast geopolymers will directly transfer to printed systems.

Long-term carbonation studies are particularly needed because current data on 3DPGPMs remain limited, especially with respect to printing direction, specimen orientation, interlayer defects, curing humidity, and realistic exposure conditions. Future studies should combine accelerated and natural carbonation tests under different relative humidity levels, CO_2_ concentrations, curing regimes, precursor compositions, and exposure durations to clarify how interlayer porosity and connected transport pathways influence carbonation progression [13]. Such studies should also consider carbonation-related changes in pore solution alkalinity, reinforcement passivation, and the combined effects of carbonation and chloride ingress in reinforced printed elements.

Long-term chemical exposure studies are also required for acid and sulphate environments. Mishra et al. [13] emphasized that durability data for 3D-printed geopolymer systems remain insufficient under aggressive exposure conditions, including acid, sulphate, and salt environments. Future studies should therefore evaluate field-relevant scenarios such as acid rain, industrial acidic exposure, sewage-related biogenic sulfuric acid attack, sulphate-bearing groundwater, seawater, industrial wastewater, and wetting–drying cycles. These investigations should explicitly consider precursor chemistry, calcium content, interlayer time interval, interlayer porosity, pore connectivity, printing direction, and specimen orientation because these variables may strongly influence acid and sulphate ingress, damage development, and residual mechanical performance.

Freeze–thaw durability should be assessed through long-term field exposure as well as standardized laboratory cycling. Laboratory freeze–thaw results may not fully represent realistic service conditions involving combined freezing–thawing, wetting–drying, de-icing salts, variable humidity, and interlayer-oriented moisture transport. Therefore, future studies should evaluate whether laboratory-scale freeze–thaw performance can be maintained under coupled environmental actions, particularly when connected pores, weak interlayer regions, and printing-direction-dependent moisture pathways are present.

Future durability research should combine accelerated laboratory tests with long-term field exposure programs. To enable meaningful comparison between studies, experimental programs should report printing direction, specimen extraction orientation, interlayer time interval, curing regime, exposure surface, porosity distribution, transport direction, and failure mode. From a cautious interpretation, long-term field validation should not rely on a single durability indicator; instead, it should combine residual strength, mass change, dimensional stability, visual deterioration, transport properties, microstructural damage, interlayer integrity, and environmental exposure history. Such integrated datasets are necessary to develop reliable durability models and service-life prediction tools for 3DPGPMs.

### 8.3. Structural-Scale Validation

The limited number of field-scale geopolymer 3DCP applications highlights the need for structural-scale validation under realistic construction and service conditions. Most existing studies on 3D-printed geopolymer composites are still based on mortar-scale mixtures, fine-aggregate systems, or small laboratory specimens, which are useful for understanding printability, interlayer bond formation, anisotropy, and hardened material properties [1,15]. However, these material-level investigations are not sufficient to validate the performance of load-bearing structural elements. Therefore, future research should move toward element-scale testing of beams, wall panels, slabs, columns, and joints under realistic loading, curing, environmental exposure, and construction conditions.

**Figure 23:**
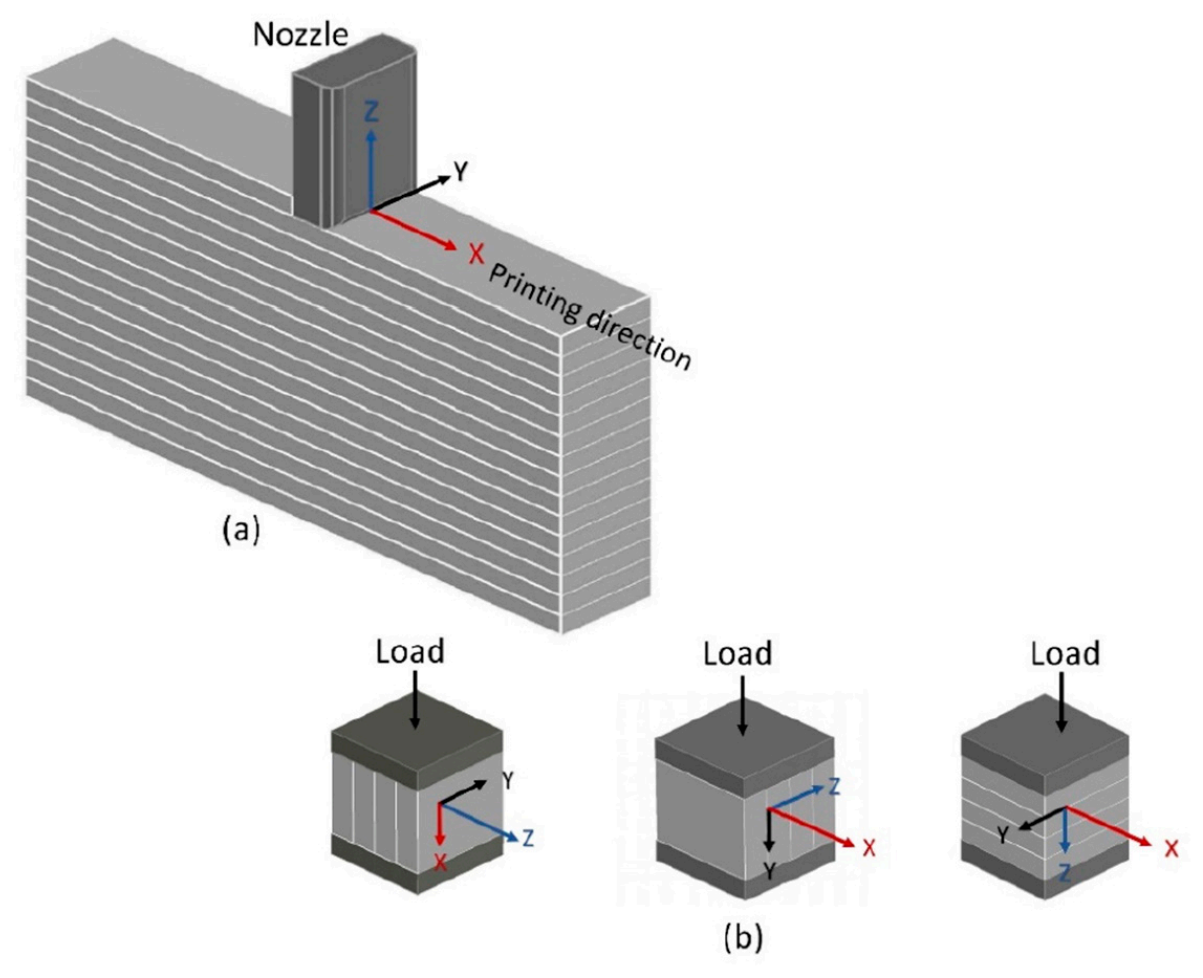
(**a**) Definition of the X, Y, and Z directions relative to the printing direction and layer orientation; (**b**) compressive loading configurations applied along the X, Y, and Z directions for evaluating anisotropic mechanical behavior in 3D-printed cementitious materials. Reprinted from Rehman and Kim [6].

**Figure 24:**
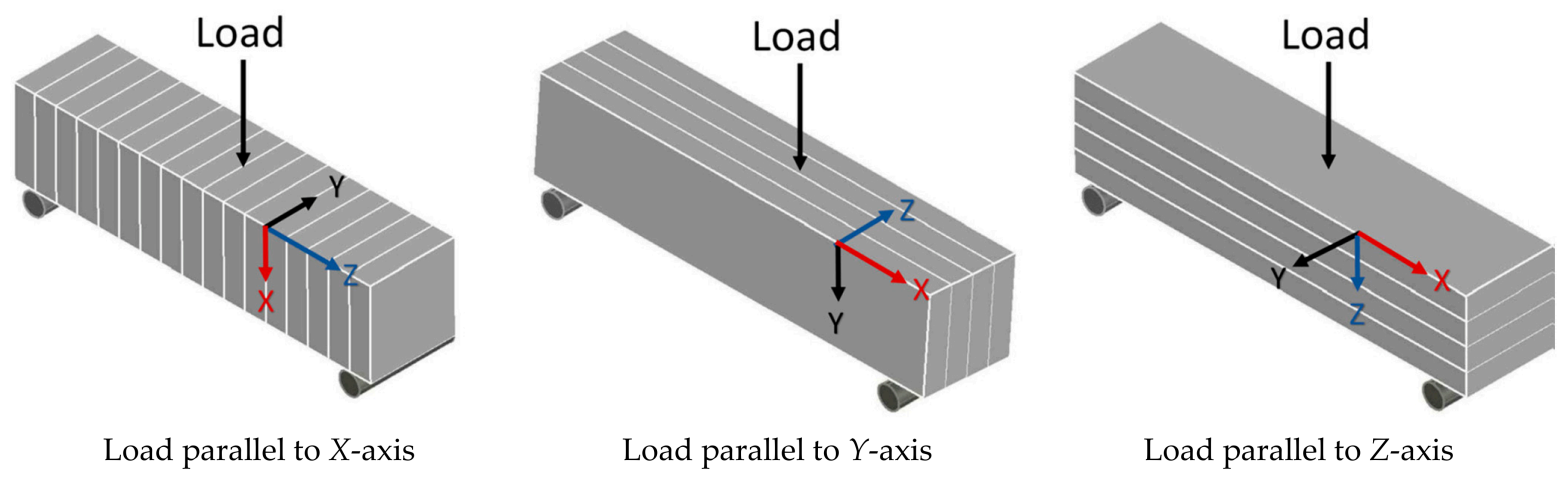
Designation of X, Y, and Z directions for evaluating anisotropy in flexural strength of printed cementitious elements, Reprinted from Rehman and Kim [6].

Mechanical anisotropy is one of the main issues that must be addressed in structural-scale validation. In 3D-printed geopolymer elements, anisotropy is strongly related to interlayer bond quality, cold-joint formation, layer orientation, and the loading direction relative to the printed layers [2,67]. This means that structural design approaches should not rely only on conventional material strength parameters. Instead, design and verification procedures should explicitly consider the compatibility between printing direction and load direction, interlayer interface quality, reinforcement strategy, and structural-scale load transfer mechanisms [7]. Figure 23 and Figure 24 illustrate how printing direction and loading orientation should be defined when evaluating anisotropic compressive and flexural behavior.

Structural-scale validation should also include reinforcement integration, because unreinforced printed geopolymer mortars are unlikely to satisfy the tensile, flexural, crack-control, and ductility requirements of many load-bearing applications. Reviews on structural 3D-printed concrete indicate that steel bars, meshes, cable or wire systems, fiber-hybrid strategies, and printed permanent formwork approaches can improve tensile resistance, flexural capacity, crack control, ductility, and constructability [7,45]. Recent beam-scale studies have also shown that in-process reinforcement integration can improve load transfer and flexural performance in 3D-printed concrete elements [68]. However, for geopolymer-based printed elements, standardized procedures for reinforcement design, detailing, placement, bonding, and production are still not mature. Further studies are therefore needed to validate reinforced 3D-printed geopolymer elements under realistic loading, curing, environmental exposure, and long-term durability conditions.

From a long-term durability perspective, structural-scale validation should not be limited to short-term strength testing. Shrinkage, creep, thermal cracking, moisture cycling, chloride exposure, carbonation, freeze–thaw action, and marine exposure may become concentrated around interlayer regions in printed elements. Large-scale studies on cement-based 3D printing have shown that degradation mechanisms can be strongly influenced by layer geometry, interlayer voids, pore orientation, and pore connectivity, indicating that element-scale durability cannot be predicted from bulk matrix properties alone [66]. For 3D-printed geopolymer systems, available studies have mainly focused on fresh behavior, mechanical performance, interlayer bond strength, microstructure, and pore structure, while comparative long-term field data on carbonation, chloride migration, coupled environmental exposure, and low-alkali or one-part mixtures remain scarce [16,20].

This lack of long-term and structural-scale data creates uncertainty in design, performance monitoring, and code development for 3D-printed geopolymer systems. Therefore, strength models that account for mechanical anisotropy, standardized interlayer bond testing, reinforcement integration protocols, and structural-scale validation procedures should be considered key components of the transition from laboratory research to practical construction [7,20]. In addition, combined accelerated and natural exposure programs are needed to evaluate structural and durability performance under realistic service environments, while explicitly considering printing direction, interlayer quality, reinforcement placement, and environmental actions.

**Figure 25:**
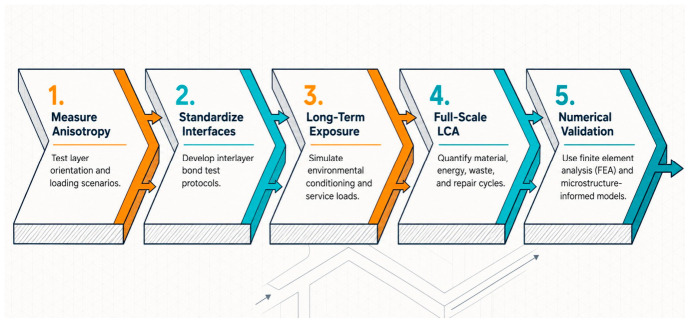
Five-step roadmap for structural-scale validation of 3D-printed geopolymer systems. The arrows indicate the sequential progression and interconnection of the five proposed research and validation steps. Conceptual synthesis based on Raphael et al. [7], Yao et al. [17], Khan et al. [18], Tinoco et al. [19], and Motalebi et al. [21].

A structural-scale validation roadmap for 3D-printed geopolymer systems should include five interconnected steps. This roadmap is schematically summarized in Figure 25. First, printing direction, layer orientation, and loading direction should be systematically varied in element-scale specimens to quantify anisotropy-sensitive behavior. Second, common test protocols should be developed for interlayer bond strength, interface fracture, mechanical anisotropy, reinforcement integration, and structural load transfer, supported by interlaboratory comparisons to improve reproducibility [7]. Third, accelerated and natural exposure programs should be conducted on structural elements to monitor long-term performance under moisture cycling, chloride exposure, carbonation, freeze–thaw action, and thermal effects. Fourth, LCA should be extended beyond binder- or mixture-level comparisons to full 3D-printed building scenarios, including printing energy, material waste, reinforcement strategy, curing regime, transport, repair, and end-of-life assumptions [17,18,19,21]. Finally, experimental results should be validated using numerical approaches such as finite element analysis, interface models, microstructure-informed durability models, and process–structure–performance simulations [7].

Overall, structural-scale validation is essential for moving 3D-printed geopolymer systems from laboratory-scale demonstrations toward routine construction practice. From a cautious interpretation, this transition requires not only stronger and more printable mixtures, but also element-level testing, reinforcement detailing, anisotropy-aware design, durability validation, numerical modeling, quality assurance, and field-scale monitoring under realistic construction and service conditions.

### 8.4. Digital Twins, Sensing, and Closed-Loop Quality Control

Digital quality control and digital-twin-assisted process optimization are emerging research directions for improving the reliability of 3DPGPMs. Since the hardened performance of printed geopolymers is governed by the coupled effects of rheology, printing parameters, interlayer quality, pore structure, reaction kinetics, and curing conditions, future studies should move beyond isolated laboratory testing toward integrated sensing, real-time monitoring, predictive modeling, and feedback control. Inline or near-line rheology monitoring, machine vision, thermal imaging, acoustic sensing, electrical resistivity, laser scanning, extrusion pressure monitoring, and non-destructive testing may support real-time assessment of print quality, dimensional stability, interlayer consistency, and defect formation. However, these methods remain largely at the laboratory scale and are not yet standardized for geopolymer-based printable systems.

Table 8 summarizes the main research gaps, methodological integrations, expected outcomes, and measurable indicators for digital quality control and digital-twin-assisted optimization of 3DPGPMs. This roadmap highlights the need to connect robotic motion planning, geopolymer thixotropy, open-time evolution, defect detection, interlayer bond assessment, sensor-based monitoring, sustainability assessment, and life-cycle-level decision-making within a unified quality-control framework.

Robotic motion control should be co-designed with the time-dependent rheology and early-age structural build-up of geopolymer mixtures. In extrusion-based printing, dynamic yield stress and plastic viscosity govern pumping and extrusion, whereas static yield stress, thixotropy, and structuration rate control shape retention and buildability after deposition [6]. For geopolymer-based systems, this coupling is even more critical because precursor composition, activator chemistry, curing condition, and printing configuration strongly affect fresh-state evolution and hardened performance [15]. Therefore, robotic path planning should be optimized not only for geometric requirements but also for material age-dependent constraints such as open time, surface stiffening, interlayer time interval, nozzle speed, extrusion flow rate, and interlayer bond development [1].

The industrial-scale applicability of 3D-printed geopolymer and cementitious systems also depends on the controllability of the printing platform. Key process parameters include nozzle travel speed, extrusion flow rate, layer height, layer width, nozzle geometry, standoff distance, trajectory planning, and synchronization between material extrusion and nozzle movement [69]. Gantry-based systems may offer advantages in repeatability, process stability, and large-scale layer deposition, while multi-axis robotic arms can provide greater geometric freedom for curved paths, complex elements, and non-planar deposition strategies. However, this additional flexibility also increases the need for real-time monitoring, adaptive process control, and automatic correction mechanisms to maintain filament geometry, interlayer contact, dimensional accuracy, and structural reliability [7,69].

A significant part of quality assurance in 3D-printed geopolymer systems should focus on process-induced defects formed during deposition. Geometric deviations, filament spreading or sagging, nozzle clogging, inconsistent extrusion, poor interlayer contact, and bonding defects may affect pore connectivity, dimensional accuracy, mechanical anisotropy, and long-term durability. Real-time extrusion monitoring methods such as motor power consumption, extrusion pressure, electrical resistivity, and computer vision have been proposed to detect material and process variations before unacceptable layers are deposited [70]. Therefore, early fault detection should be considered essential for structural-scale 3D-printed geopolymer applications, because deposition-stage defects may not be fully captured by conventional post-printing strength tests [69].

Computer vision and other non-contact monitoring techniques can provide online or near-real-time information on filament geometry, layer height, surface texture, curvature, and geometric deviations. Surface-texture-based decision-support metrics have been proposed for detecting layer-level deformities in 3D-printed concrete using computer vision and entropy-based texture analysis [71]. More recently, AI-assisted computer vision methods have been used to quantify geometrical quality indicators such as layer height, layer angle, and curvature, while also relating these indicators to rheological properties [72]. These approaches are particularly relevant for 3D-printed geopolymer systems, where time-dependent rheology, rapid structural build-up, and open-time sensitivity can strongly affect filament stability, dimensional accuracy, and interlayer quality.

Fresh-state evaluation of interlayer quality without damaging the printed structure remains a major challenge. Electrical resistivity-based methods have been proposed for detecting unbonded interlayer regions in freshly 3D-printed concrete and have shown correlations with flexural strength loss [73]. However, their application to geopolymer mixtures requires careful calibration because activator chemistry, pore solution conductivity, moisture distribution, setting state, and interlayer porosity may strongly affect sensor response. Comparative NDT studies on idealized layered concrete specimens, including X-ray radiography and ultrasonic-based methods, provide an initial basis for interface monitoring, but these approaches still require validation under realistic geopolymer printing conditions [74].

Closed-loop quality control should also move beyond geometric monitoring and include reactivity-related sensing. In 3D-printed geopolymer production, reaction kinetics, early-age moisture and ion transport, temperature, relative humidity, extrusion pressure, flow rate, and curing condition can influence rheological stability, open-time evolution, shrinkage risk, and interlayer bond development. These data may support adaptive control of printing speed, extrusion flow rate, nozzle trajectory, standoff distance, interlayer timing, and curing conditions, particularly for mixtures with rapid structural build-up and high sensitivity to activator chemistry [1,15].

Digital twins can provide a broader framework for integrating these sensing and control strategies. In 3D concrete printing, digital twins can support data exchange, process monitoring, quality control, sustainability assessment, and life-cycle-level optimization across design, manufacturing, and service stages [75]. For 3D-printed geopolymer systems, this concept could be extended to connect material preparation, activator-controlled reaction kinetics, robotic printing, interlayer quality, curing conditions, mechanical performance, durability, and service-life assessment within a single information flow. However, important challenges remain, including real-time data processing, sensor–simulation integration, material behavior modeling, data management, and the lack of standardized datasets and reference frameworks for calibration and validation [76,77,78].

A key requirement for digital twins in geopolymer 3D printing is the development of material–process submodels that couple reaction kinetics, thixotropy, open-time evolution, early-age structural build-up, and printing parameters. Such models should link activator chemistry, precursor reactivity, temperature, humidity, extrusion flow rate, printing speed, and interlayer time interval to predict buildability, interlayer bond development, pore formation, and early-age performance [1,6,15]. Within a digital-twin framework, these relationships could be continuously updated using real-time monitoring data, thereby supporting adaptive process control and life-cycle-level quality optimization [75].

Future work should integrate camera-based monitoring, pressure or force signals, environmental sensors, and NDT data through machine-learning-supported fault diagnosis and prediction. These data streams can support feedback-based adjustment of printing speed, extrusion flow rate, nozzle trajectory, standoff distance, and interlayer timing, which are key variables for controlling print quality, buildability, extrudability, and interlayer performance [69]. For one-part and low-alkali geopolymer systems, this closed-loop approach is especially important because their workability–stability balance may be highly sensitive to open time, thixotropy, activator dissolution, moisture condition, and early-age stiffening. From a cautious perspective, digital twins should therefore be developed not only as process visualization tools but also as predictive quality-control systems linking material behavior, robotic manufacturing, defect detection, sustainability, and long-term performance.

## 9. Conclusions

This review examined the hardened performance of extrusion-based 3DPGPMs by integrating mechanical properties, durability, sustainability, standardization, and practical implementation. The main conclusion is that 3DPGPMs should not be evaluated only as printable low-carbon mixtures or as printed versions of cast geopolymers. They should instead be considered as layered, process-dependent composites whose performance is governed by the coupled effects of binder chemistry, fresh-state rheology, printing parameters, curing regime, pore structure, interlayer bonding, and loading or exposure direction.

The most critical finding is the central role of the layer interface. Interlayer regions may act as weak planes under tensile and flexural loading, while also serving as preferential transport zones for water, chlorides, CO_2_, acids, sulphates, and other aggressive agents. Therefore, mechanical anisotropy and durability degradation are closely connected through interlayer bond quality, pore connectivity, printing interval, surface moisture condition, and specimen orientation.

The review also shows that material design and manufacturing parameters must be optimized together. Precursor type, activator composition, aggregate/binder ratio, additives, fibers, reinforcement strategy, and curing regime influence hardened performance, but their effects are strongly mediated by pumpability, extrudability, buildability, structural build-up, and interlayer contact. Consequently, compressive strength alone is not sufficient for performance evaluation; direction-dependent mechanical testing, interlayer bond assessment, pore-structure characterization, and durability-related transport tests are also required.

From a sustainability and implementation perspective, the benefits of 3DPGPMs are promising but conditional. The use of waste-derived or locally available aluminosilicate precursors and formwork-free digital fabrication can reduce environmental impact, but activator production, sodium silicate demand, curing energy, precursor processing, transport distance, equipment cost, quality-control requirements, and field-scale reliability must also be considered. Thus, environmental and economic feasibility should be evaluated through combined life-cycle and cost-related assessments.

Future research should focus on integrated datasets that connect time-dependent rheology, geopolymer reaction products, multi-scale pore structure, interlayer interface quality, mechanical anisotropy, and durability performance under standardized testing conditions. Long-term field exposure studies, structural-scale validation, performance-based acceptance criteria, digital monitoring, and predictive material–process–microstructure–durability models are particularly needed to support the transition of 3DPGPMs from laboratory-scale mixtures to reliable, durable, and sustainable construction applications.

## Figures and Tables

**Table 1:** Key material, curing, and additive-related parameters influencing the printability and hardened performance of 3DPGPMs.

Parameter	Primary Area of Influence	Main Effect on Hardened Performance	Sources
GGBFS/FA ratio	Precursor reactivity, setting behavior, rheology, thixotropy, printability, early-age strength development	Partial replacement of FA with GGBFS can improve rheological stability, thixotropy, yield stress, and early structural build-up of 3DPGPMs. The higher CaO and amorphous content of GGBFS can promote faster geopolymerization and denser early-age microstructure; however, excessive GGBFS shortens setting/open time and may reduce the printable time window.	Guo et al. [22]; Panda et al. [23]
Curing/early-age thermal activation condition	Open time, buildability, early-age structural build-up, geopolymerization rate, interlayer bond development, directional mechanical performance	Ambient-temperature curing is more practical for large-scale/on-site 3D printing of one-part geopolymers, provided that sufficient open time, extrudability, buildability, and strength are achieved. Microwave-assisted set-on-demand treatment can accelerate polycondensation and structural build-up, improving buildability and interlayer bond strength; however, excessive heating may increase surface moisture loss.	Bong et al. [26]; Muthukrishnan et al. [9]
Fiber reinforcement	Tensile/flexural strength, anisotropy, crack control, post-cracking response, fiber–matrix interaction	Short fibers can improve tensile/flexural strength and crack control in 3DPGPMs, but their effect on compressive strength may be limited and direction-dependent. In 3D-printable engineered cementitious composite (ECC)-type composites, optimized fiber reinforcement can enable strain-hardening, multiple cracking, and higher energy absorption.	Panda et al. [29]; Zhu et al. [30]
Nano-additives	Matrix densification, pore refinement, rheology, thixotropic build-up	Nano-additives can improve strength by densifying the matrix and reducing porosity, while also improving printability through rheological and thixotropic control.	Chougan et al. [24]

**Table 2:** Direction-dependent failure mechanisms and typical mechanical response of 3DPGPMs.

Loading Direction	Definition	Dominant Failure Mechanism	Typical Mechanical Response	Sources
X–Y plane	Loading parallel to the printed layer plane	Matrix cracking, fiber rupture, or fiber pull-out within printed filaments	Relatively higher tensile/flexural resistance when filament continuity and fiber distribution along the printed path are effective	Panda et al. [29,33]; Barve et al. [1]
Z direction	Loading perpendicular to the printed layers	Delamination or separation along weak interlayer interfaces	Relatively lower tensile/flexural resistance when failure is governed by weak interlayer bonding	Al-Qutaifi et al. [31]; Panda et al. [29,33]; Barve et al. [1]

**Table 3:** Main short fiber types used in 3DPGPMs and their expected mechanical contributions.

Reinforcement Type	Main Contribution to Mechanical Performance	Potential Limitation	Sources
Synthetic polymer fibers: Polypropylene (PP), polyvinyl alcohol (PVA), Polyethylene (PE)	Can improve crack control, ductility, toughness, shrinkage control, and post-cracking behavior. PE and PVA fibers are especially effective in broader 3D-printable ECC systems with high tensile ductility	Excessive dosage may reduce workability, increase extrusion resistance, cause nozzle blockage, or lead to non-uniform fiber distribution. Evidence from Zhu et al. [30] should be interpreted as ECC/cementitious-system evidence, not direct geopolymer evidence	Lv et al. [35]; Zhu et al. [30]
Glass fibers	Improve flexural and tensile performance of 3DPGPMs and contribute to direction-dependent mechanical response depending on fiber content, fiber length, and loading direction	Fiber effectiveness depends on dispersion, fiber–matrix bond, and interlayer bond quality. Some glass fibers may be vulnerable in highly alkaline matrices unless alkali-resistant fibers or suitable surface treatments are used	Panda et al. [29]; Lv et al. [35]
Basalt and other mineral/natural fibers	Provide potential benefits in terms of crack control, toughness, sustainability, and thermal stability depending on fiber type and matrix compatibility	Long-term durability, alkali resistance, fiber dispersion, and matrix compatibility must be verified for each geopolymer system	Lv et al. [35]

**Table 4:** Selected studies on durability-related performance of 3DPGPMs and hybrid geopolymer systems.

Study	Binder System	Printing/Processing Details	Exposure or Tested Property	Main Findings
Marczyk et al. [43]	Hybrid system: 95% concrete + 5% geopolymer binder based on FA or MK	3D printing and conventional casting; aramid roving reinforcement	Freeze–thaw resistance, UV resistance, and thermal conductivity	3D-printed concrete–geopolymer hybrid specimens generally showed a smaller reduction in compressive strength after freeze–thaw exposure than cast specimens. However, their absolute compressive strength remained lower than that of cast specimens, probably due to weaker interlayer connections.
Jaji et al. [16]	MK + 0–30% slag	Extrusion-based 3D printing; optimized aggregate/binder ratio of 2.0; sodium phosphate retarder	Mechanical performance, interlayer bond strength, and pore structure	Increasing slag content improved compressive, flexural, interlayer bond, and splitting tensile strength. Microstructural results showed increased formation of C-S-H, N-A-S-H, and C-A-S-H gels, denser morphology, pore refinement, and enhanced BET surface area.
Tanyildizi et al. [42]	MK + FA + nano-ZnO	Extrusion-based 3D printing; nano-ZnO dosages of 0–0.75%	Freeze–thaw resistance	The optimum nano-ZnO dosage was 0.5%. This mixture showed the highest freeze–thaw resistance, with 95.72% compressive strength retention after freeze–thaw exposure. The improvement was associated with pore-filling, matrix densification, and improved microstructural stability.
Gardezi et al. [41]	MK + FA + nano-ZnO	Extrusion-based 3D printing; nano-ZnO dosages of 0–0.75%	Acid attack resistance under 5% HCl exposure	The 0.5% nano-ZnO mixture showed the best acid resistance. Compared with the reference mixture, the acid-exposed 0.5% nano-ZnO mixture exhibited 33.64% higher compressive strength and 42.86% higher flexural strength. SEM-based observations indicated a denser matrix, lower porosity, and improved gel-related pore refinement.

**Table 5:** Qualitative comparison of durability-related performance of 3DPGPMs, cast geopolymers, and OPC-based 3D-printed cementitious systems based on selected literature.

Durability Parameter	3DPGPMs	Cast Geopolymers	OPC-Based 3D-Printed Systems	Scientific Commentary
Shrinkage and cracking	Medium to high risk, especially under open extrusion and rapid drying	Low to medium, depending on curing and moisture control	Medium to high	Open extrusion increases the exposed surface area and may accelerate early-age moisture loss; fibers, nanocellulose, and shrinkage-reducing strategies can reduce cracking [13,27,28,44]
Water absorption and permeability	Direction-dependent; may be low in dense matrices but higher across weak interfaces	Generally low when dense and well cured	Direction-dependent	Layer interfaces and connected pores may act as preferential transport pathways if interlayer compaction and bonding are insufficient [13,16,43]
Chloride penetration	Direction-dependent; potentially low in dense matrices but vulnerable along interlayers	Low to medium, depending on pore structure and binder chemistry	Direction-dependent	Interlayer voids, cold joints, printing interval, and specimen orientation can strongly influence chloride ingress in printed systems [13,28,43,47,48]
Carbonation	Medium; direct data remain limited for printed geopolymers	Variable, depending on Ca content, alkali reserve, and pore structure	Medium to high	CO_2_ ingress may be accelerated by interlayer microcracks, cold-joint regions, and connected pores; direct carbonation data 3DPGPMs remain limited [6,13,15,28,51]
Acid attack	Potentially high resistance, especially in low-Ca systems; weak interfaces may reduce performance	Generally high, depending on precursor chemistry	Low to medium	Geopolymer chemistry may improve acid resistance, but acid ingress through weak layer interfaces, elongated pores, or microcracks can cause internal weakening in printed systems [28,43,52,53]
Sulphate attack	Potentially high resistance, but insufficiently verified for printed systems	Generally high in low-Ca systems	Medium to low	Reduced portlandite content and limited ettringite-related expansion may improve sulphate resistance in geopolymer systems, but interlayer porosity can promote sulphate ingress in printed elements [6,13,52]
Freeze–thaw resistance	Medium to high when pore structure, air-void system, and interlayer integrity are controlled	Generally high when dense and properly cured	Medium; often requires air entrainment	Directional pores and weak interlayers may accumulate water and promote ice-induced damage; air-entraining admixtures and nano-additives may improve performance when properly optimized [,42,43,54,55]
High-temperature performance	Potentially high, depending on precursor chemistry and moisture content	High in many geopolymer systems	Medium; spalling risk may occur in dense systems	Some geopolymer matrices may show favorable residual strength at elevated temperature, while printed layers may be vulnerable to thermal cracking, moisture-pressure damage, or delamination depending on pore structure and interlayer quality [6]

**Table 6:** Qualitative comparison of OPC concrete, cast geopolymer concrete, and 3D-printed geopolymer concrete in terms of CO_2_ emissions, activator impact, mixture design sensitivity, waste material use, energy consumption, and overall GWP, adapted from Mir et al. [57], Khan et al. [58], Yao et al. [17], Chen et al. [44], and Mishra et al. [13].

Characteristic	OPC Concrete	Cast Geopolymer Concrete	3D-Printed Geopolymer Concrete
CO_2_ Emissions	Highest	Medium	Lowest
Activator Impact	Not applicable	Significant	Significant
Mixture Design Sensitivity	Low	Medium	High
Waste Material Use	Low	Medium	High
Energy Consumption	Medium	Medium	High
Overall GWP	High	Medium	Low

**Table 7:** Proposed performance-based acceptance criteria for 3DPGPMs, synthesized based on the RILEM TC 276-DFC framework and selected studies on geopolymer-based 3D printing [5,8,10,23,25].

Acceptance Category	Key Parameters to Be Evaluated	Purpose of Evaluation
Printability	Pumpability, extrudability, filament continuity, nozzle blockage risk	To verify stable material delivery and continuous extrusion during printing
Buildability and shape stability	Structural build-up, yield stress development, layer deformation, dimensional stability	To ensure that printed layers can support subsequent layers without excessive deformation
Open-time control	Interlayer time interval, surface stiffening, moisture condition, setting progress	To define the printable time window and prevent cold-joint formation
Interlayer bond quality	Tensile/shear bond strength, interlayer cohesion, failure mode, layer-interface defects	To evaluate mechanical continuity between successive printed layers
Mechanical anisotropy	Compressive, tensile, and flexural strength in different loading and printing directions	To quantify direction-dependent mechanical behavior caused by layer-by-layer deposition
Durability-related transport	Water absorption, sorptivity, permeability, chloride ingress, carbonation depth, pore connectivity	To assess long-term resistance against moisture, gas, and aggressive ion transport
Curing sensitivity	Temperature, humidity, curing duration, moisture retention, early-age strength development	To verify whether laboratory performance can be maintained under realistic site conditions
Process quality control	Nozzle geometry, layer height, printing speed, extrusion flow rate, residence time, environmental conditions	To link material performance with actual printing parameters and equipment conditions
Field implementation	Batch-to-batch consistency, activator handling safety, dry-mix stability, documentation, certification pathway	To support reproducibility, regulatory acceptance, and industrial-scale use

**Table 8:** Research roadmap for digital quality control, sensing, and digital-twin-assisted optimization of 3DPGPMs.

Research Gap	Proposed Methodological Integration	Expected Outcome	Measurable KPI/Illustrative Target Examples
Lack of quantified rules for co-designing robotic motion planning with geopolymer thixotropy and setting window	Joint optimization of robot speed, acceleration profile, rheometer-based structural build-up rate, open time, and multi-axis path planning	Material-aware robotic printing strategies	Filament width and layer height deviation within project-defined tolerance limits; reduced nozzle clogging frequency; stable printable time window
Lack of geopolymer-specific reference datasets and defect classes for real-time geometric monitoring	AI-assisted computer vision, automated defect calibration, and openly labeled image/video datasets	Standardized visual quality inspection protocols	Defect detection accuracy > 90% under validated datasets; element-scale geometry tolerance typically within 3–5 mm, depending on project requirements
Limited sensor/non-destructive testing (NDT) integration for detecting fresh-state interlayer defects	Electrical resistivity, ultrasonic, acoustic, or multimodal NDT sensors placed near/behind the nozzle and correlated with mechanical interlayer bond tests	Early detection of interlayer defects and bond-related risk reduction	Prediction error for interlayer bond strength loss ≤ 10–15%; project-specific threshold for unbonded or poorly bonded interface area
Lack of integrated reaction–rheology–microstructure models and unified sensor streams within digital twins	Multi-sensor data fusion and machine-learning-assisted model updating for in-process prediction of rheology, buildability, early-age stiffness, and hardened performance	Closed-loop quality control and performance prediction	Digital-twin prediction error ≤ 10–15% for early-age stiffness, yield stress evolution, compressive/flexural strength, or interlayer bond strength
Limited application of digital twins for sustainability and cost optimization	Integration of energy consumption, material waste, reprinting, maintenance, repair, and LCA data into digital-twin scenarios	Environmentally and economically optimized printing strategies	Reduction in printing energy demand or material waste; reprinting rate < 2%; reduced kg CO_2_e/m^3^ or kg CO_2_e per printed element

## Data Availability

No new data were created or analyzed in this study. Data sharing is not applicable to this article.

## Nomenclature

**Abbreviation**	**Definition**
3D	Three-dimensional
3DCP	Three-dimensional concrete printing
3DPGPC	Three-dimensional printed geopolymer concrete
3DPGPM	Three-dimensional printed geopolymer mortar
ASTM	American Society for Testing and Materials/ASTM International
C-(A)-S-H	Calcium-(alumino)-silicate hydrate
C-A-S-H	Calcium aluminosilicate hydrate
C-S-H	Calcium silicate hydrate
CDW	Construction and demolition waste
ECC	Engineered cementitious composite
FA	Fly ash
GGBFS/GGBS	Ground granulated blast-furnace slag
GWP	Global warming potential
ISO	International Organization for Standardization
LCA	Life-cycle assessment
MK	Metakaolin
N-A-S-H	Sodium aluminosilicate hydrate
OPC	Ordinary Portland cement
PE	Polyethylene
PP	Polypropylene
PVA	Polyvinyl alcohol
RILEM	International Union of Laboratories and Experts in Construction Materials, Systems and Structures
SWOT	Strengths, weaknesses, opportunities, and threats
TS EN	Turkish Standard adopting a European Standard
TÜBİTAK	Scientific and Technological Research Council of Türkiye
TÜBİTAK-BİDEB	TÜBİTAK Directorate of Science Fellowships and Grant Programmes
WGP	Waste glass powder
